# Supplementary figures and images for: The cytidine deaminase APOBEC3A regulates nucleolar function to promote cell growth and ribosome biogenesis
Source: PLoS Biol. 2024 Jul 8;22(7):e3002718. doi: 10.1371/journal.pbio.3002718 (PMC11257408; doi:10.1371/journal.pbio.3002718)

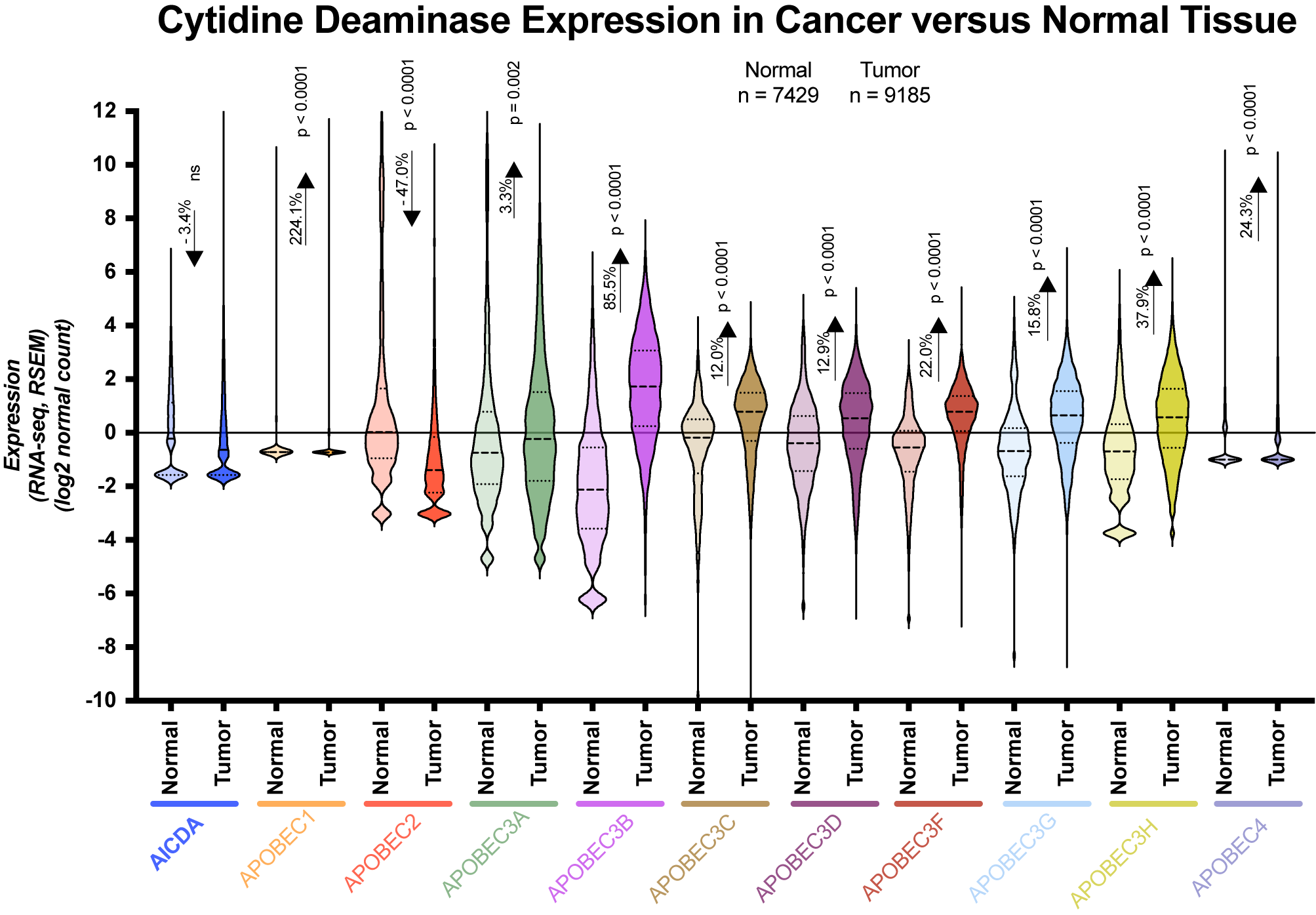

Supplement: S1 Fig — Violin plots from Genotype-Tissue Expression (GTEx) unmatched normal and The Cancer Genome Atlas (TCGA) matched normal and tumor RNA-seq by Expectation-Maximization (RSEM) [51] Log2 fold expression levels for human cytidine deaminases subtracted from the mean. Mean of tumor (N = 9,185) and normal (N = 7,429) cytidine deaminase expression set at 0 (horizontal line). For each indicated transcript, median (heavy dashed line), quartiles (light dashed line), normal data (light shading), and tumor data (darker shading). Percent change in expression between normal versus tumor indicated for each cytidine deaminase transcript. Data were analyzed by Student’s t test, significant p-values are reported on the graph. All underlying numerical values for figure found in S2 Data. (TIF) [file pbio.3002718.s001.tif]

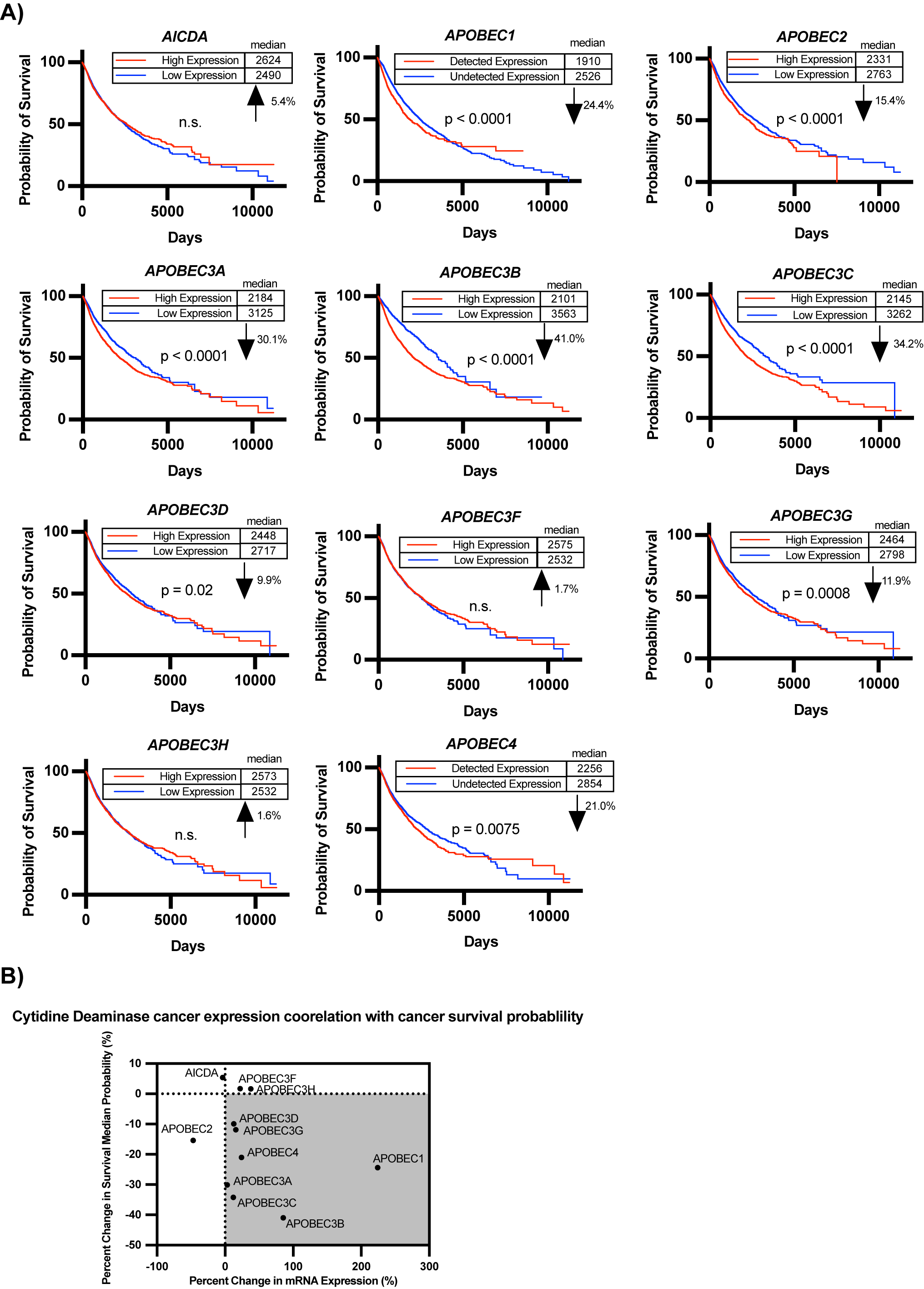

Supplement: S2 Fig — (A) Higher expression of 8 out of the 11 human cytidine deaminases correlates with decreased survival probability. Survival data was obtained from Genotype-Tissue Expression (GTEx) unmatched normal samples and The Cancer Genome Atlas (TCGA) matched normal and tumor samples using the Xena platform [51]. Samples were stratified by either high or low cytidine deaminase expression and survival curves were generated. For cytidine deaminases where the median expression was 0, data was stratified by either detected or undetected levels of expression. Median survival (days) and the percent difference between low and high expression groups were reported. Data were analyzed by Kaplan–Meier survival analysis, significant p-values reported on respective graphs. (B) Seven out of the 11 human cytidine deaminases exhibit higher expression in tumor versus normal tissue and higher expression is associated with lower survival probability. Percent change in mRNA expression from (S1) (x-axis) and percent change in median survival from (A) (y-axis) was plotted for each cytidine deaminase. An increase in expression and a decrease in survival quadrant are indicated by the gray background. All underlying numerical values for figure found in S2 Data. (TIF) [file pbio.3002718.s002.tif]

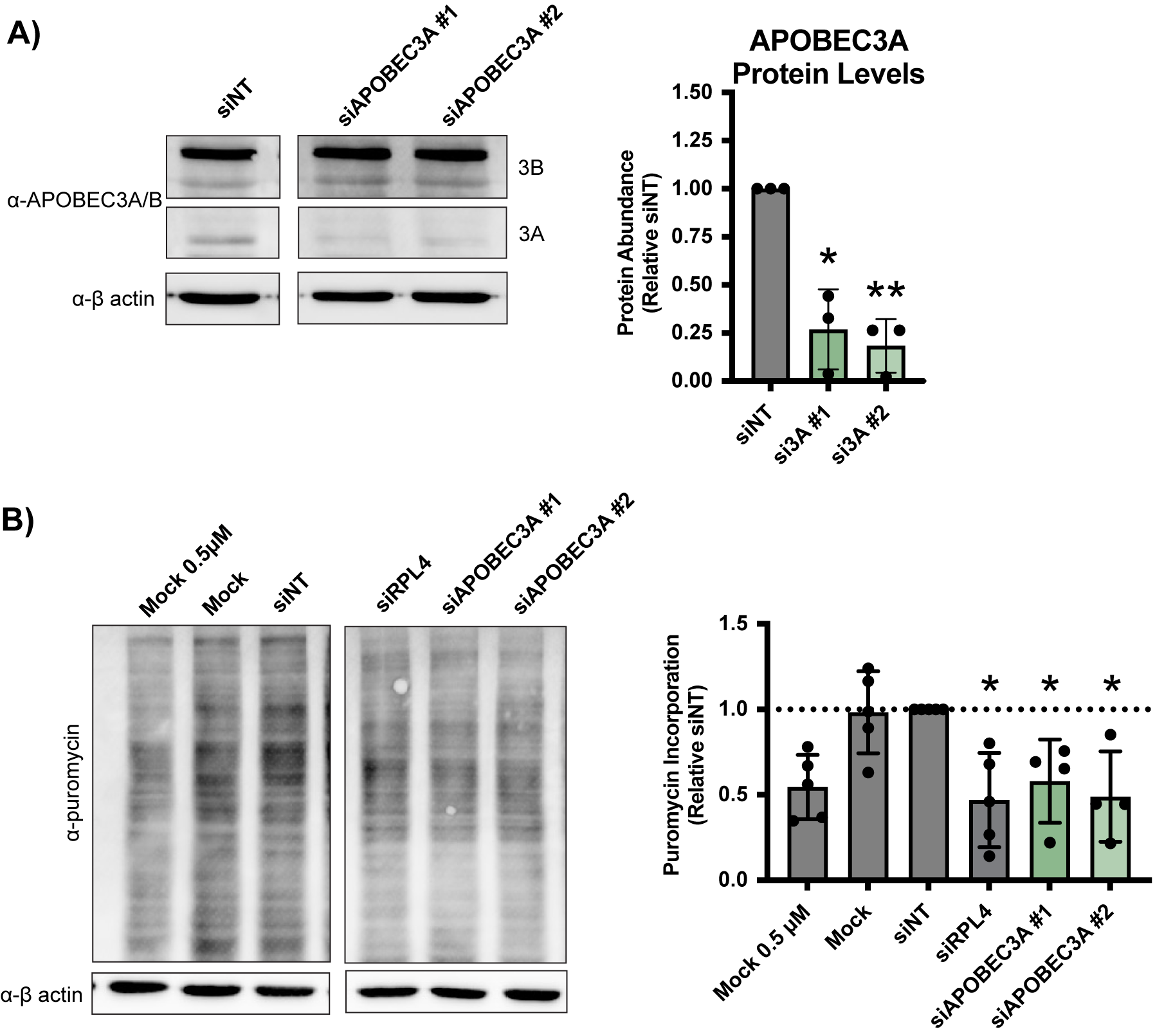

Supplement: S3 Fig — (A) Validation of APOBEC3A protein depletion after siAPOBEC3A individual siRNA #1 and #2 treatment. (Left) Representative western blot using an α-APOBEC3A/B antibody. siNT is a negative control. α-β-actin is a loading control. (Right) Quantification of APOBEC3A protein levels normalized to β-actin signal and relative to siNT negative control. Three biological replicates plotted mean ± SD. Data were analyzed by Student’s t test, ** p ≤ 0.01, * p ≤ 0.05. (B) Depletion with individual siAPOBEC3A #1 and #2 reduces global protein synthesis in MCF10A cells. After 72 h siRNA depletion, 1 μM puromycin was added for 1 h to measure translation. (Left) Representative western blot using an α-puromycin antibody. Mock and siNT are negative controls, siRPL4 is a positive control, and Mock 0.5 μM is a control to indicate robust quantification. α-β-actin is shown as a loading control. (Right) Quantification of puromycin signal normalized to β-actin signal and relative to siNT negative control. Four biological replicates were plotted mean ± SD. Data were analyzed by one-way ANOVA with Dunnett’s multiple comparisons test, * p ≤ 0.05. All underlying numerical values for figure found in S2 Data. (TIF) [file pbio.3002718.s003.tif]

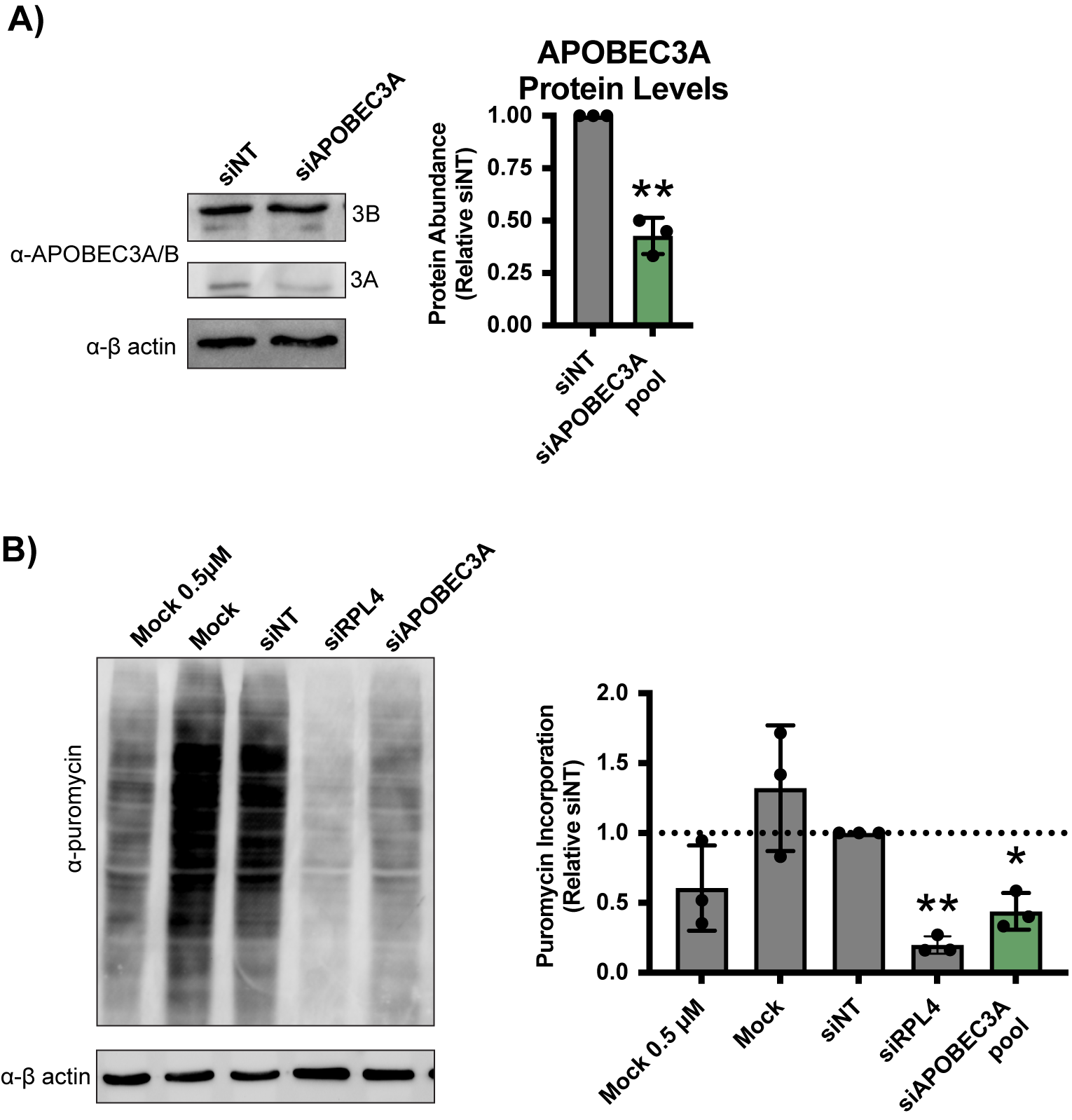

Supplement: S4 Fig — (A) Validation of APOBEC3A protein depletion after siAPOBEC3A pool treatment in HeLa cells. (Left) Representative western blot using an α-APOBEC3A/B antibody. siNT is a negative control. α-β-actin is shown as a loading control. (Right) Quantification of APOBEC3A protein levels normalized to β-actin signal and relative to siNT negative control. Three biological replicates plotted mean ± SD. Data were analyzed by Student’s t test, ** p ≤ 0.01. (B) siAPOBEC3A pool treatment reduces global protein synthesis in HeLa cells. After 72 h siRNA depletion, 1 μM puromycin was added for 1 h to measure translation. (Left) Representative western blot using an α-puromycin antibody. Mock and siNT are negative controls, siRPL4 is a positive control, and Mock 0.5 μM is a control to indicate robust quantification. α-β-actin is shown as a loading control. (Right) Quantification of puromycin signal normalized to β-actin signal and relative to siNT negative control. Three biological replicates plotted mean ± SD. Data were analyzed by one-way ANOVA with Dunnett’s multiple comparisons test, ** p ≤ 0.01, * p ≤ 0.05. All underlying numerical values for figure found in S2 Data. (TIF) [file pbio.3002718.s004.tif]

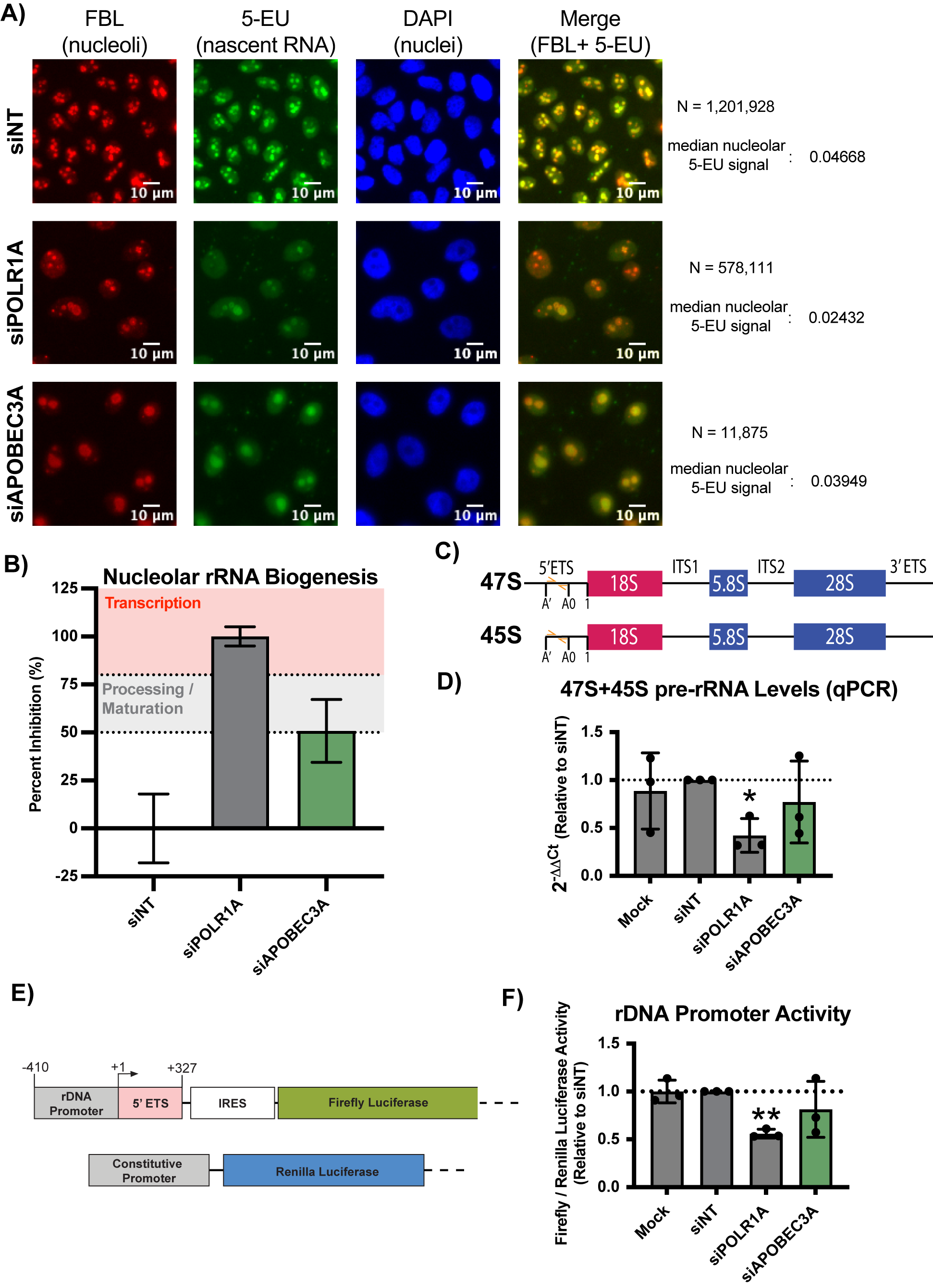

Supplement: S5 Fig — (A, B) siAPOBEC3A depletion (pool) modestly reduces nucleolar rRNA biogenesis in MCF10A cells. (A) After 72 h siRNA depletion, 1 mM 5-EU was added for 1 h to measure nucleolar rRNA biogenesis. Representative images of nucleoli stained with α-fibrillarin (FBL, red), 5-ethynl uridine (5-EU) visualized by click-chemistry attached AF488 azide (green), nuclei stained with DAPI (blue), and FBL and 5-EU merged (yellow). siNT is a negative control and siPOLR1A (large subunit of RNA Polymerase 1) is a positive control. N = number of nuclei (cells) analyzed in all 3 biological replicates and median nucleolar 5-EU signal (nucleolar rRNA biogenesis) reported. (B) Quantification of nucleolar rRNA biogenesis percent inhibition. siNT negative control is set to 0% and siPOLR1A positive control is set to 100%. Based on results in Bryant and colleagues [71], factors required for pre-rRNA transcription have a percent inhibition > ~80% (red background) and factors only required for pre-rRNA processing/maturation have a percent inhibition approximately 50% to 80% (gray background). Three biological replicates are plotted mean ± SD. (C) Schematic of 47S+45S pre-rRNA transcript measured by qRT-PCR in (D) using the indicated primers (orange). (D) siAPOBEC3A depletion (pool) does not significantly reduce 47S+45S pre-rRNA transcript levels. qRT-PCR was performed to measure primary 47+45S pre-rRNA transcript levels. Mock and siNT are negative controls and siPOLR1A is a positive control; 2-ΔΔCT measured relative to 7SL internal control and siNT negative control. Three technical replicates of 3 biological replicates plotted mean ± SD. Data were analyzed by Student’s t test, * p ≤ 0.05. (E) Schematic of luciferase reporter plasmids to measure rDNA promoter activity [74]. (Top) Firefly pHrD-IRES-Luc rDNA promoter reporter plasmid. (Bottom) Renilla luciferase constitutive promoter reporter plasmid, transfection control. (F) siAPOBEC3A depletion (pool) does not significantly reduce rDNA promoter ac [file pbio.3002718.s005.tif]

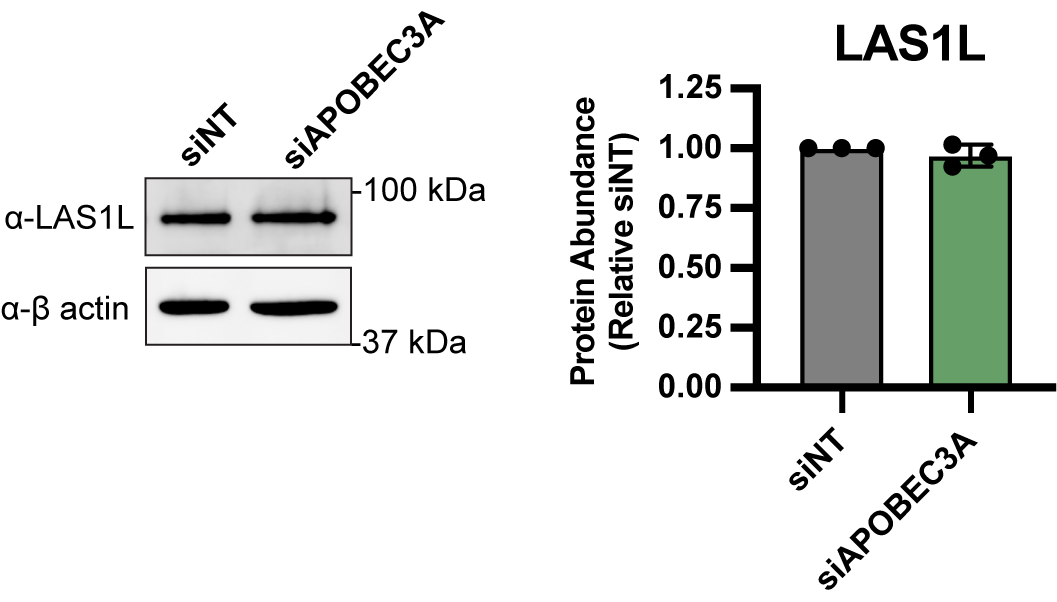

Supplement: S6 Fig — (Left) Representative western blot using α-LAS1L antibody. siNT is a negative control. α-β-actin is shown as a loading control. (Right) Quantification of LAS1L protein levels normalized to β-actin signal and relative to siNT negative control. Three biological replicates plotted mean ± SD. Data were analyzed by Student’s t test. All underlying numerical values for figure found in S2 Data. (TIF) [file pbio.3002718.s006.tif]

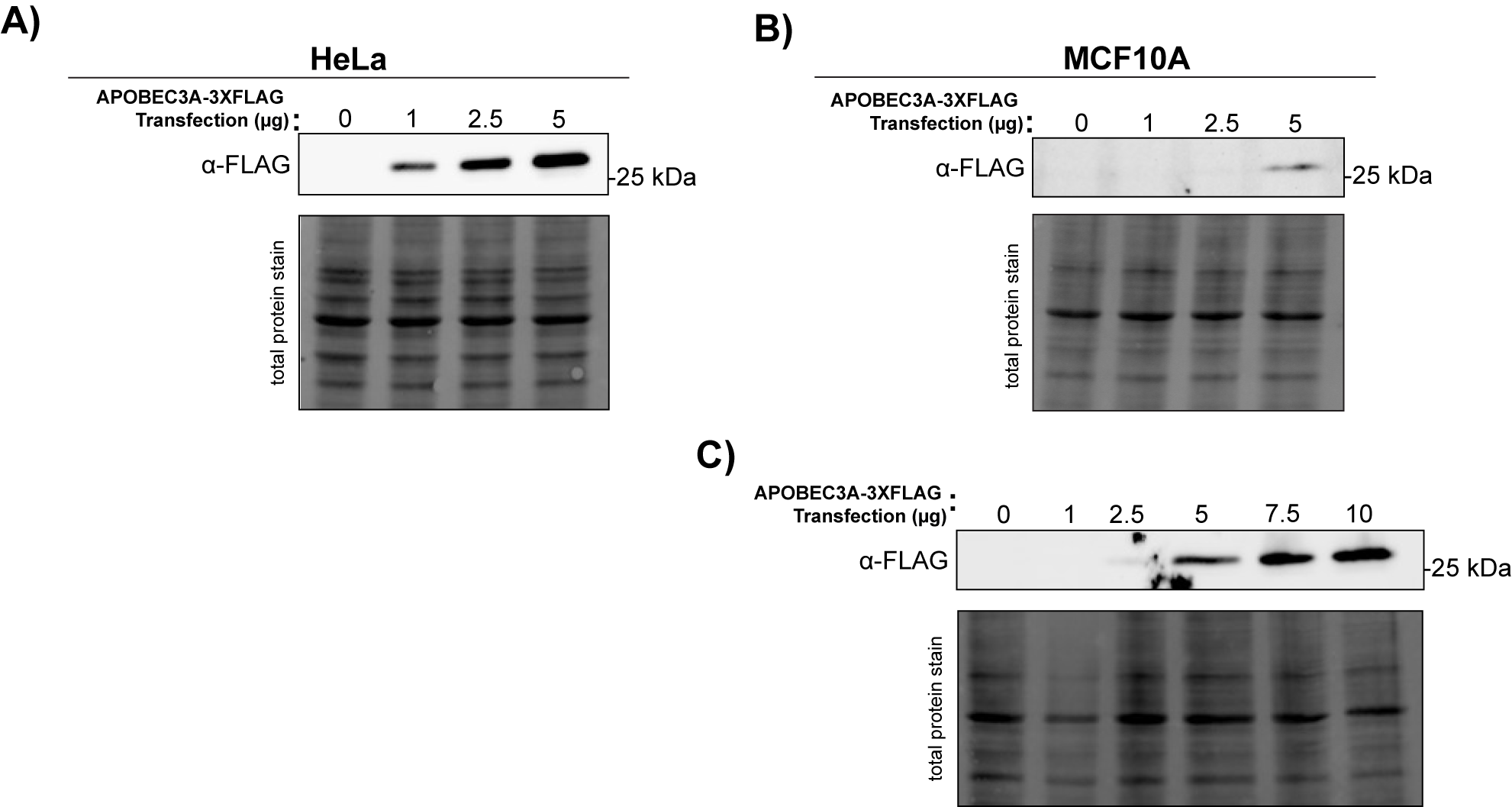

Supplement: S7 Fig — (A) Representative western blot using α-FLAG antibody on HeLa whole cell lysate. Increasing amounts of APOBEC3A-3XFLAG plasmid transfected for 24 h in 10 cm dishes (10 ml media volume). Stain-free total protein is shown as a loading control. One biological replicate. (B) Same as in (A) except using MCF10A whole cell lysate. (C) Same as in (B) except including higher amounts of plasmid. (TIF) [file pbio.3002718.s007.tif]

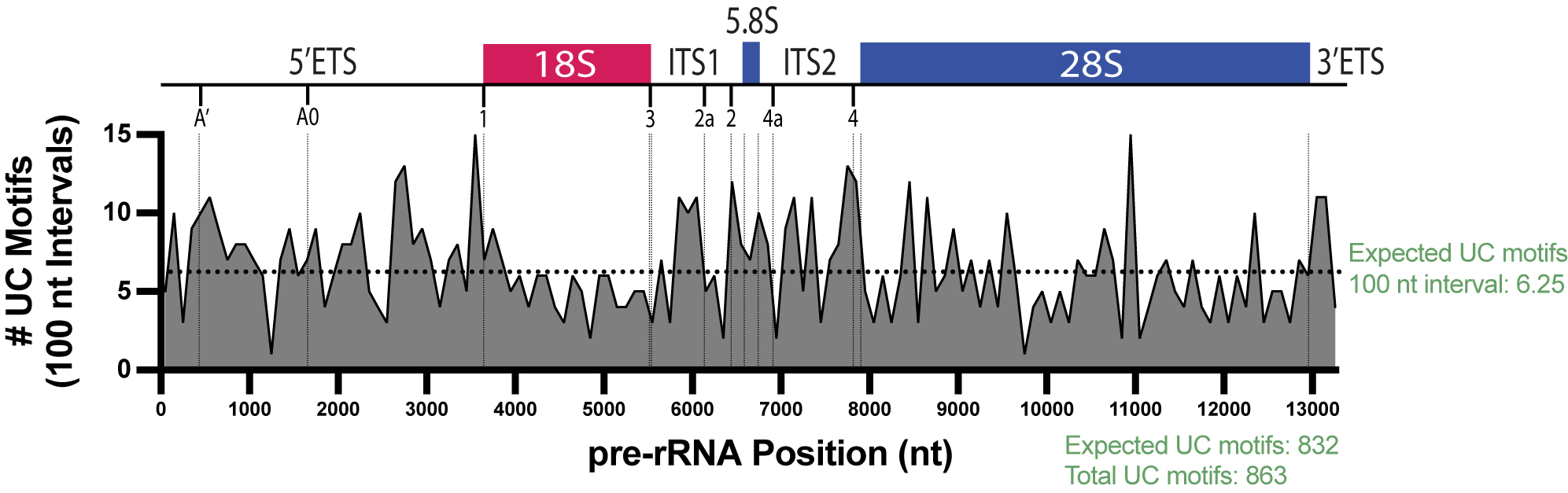

Supplement: S8 Fig — The occurrence of UC sequence motifs was calculated for every 100 nucleotide (nt) interval across the pre-rRNA and graphed. The occurrence of UC sequence motif at random over 100 nt is predicted to be 6.25, indicated by the dotted horizontal line. Pre-rRNA cleavage sites are indicated with vertical solid lines. All underlying numerical values for figure found in S2 Data. (TIF) [file pbio.3002718.s008.tif]

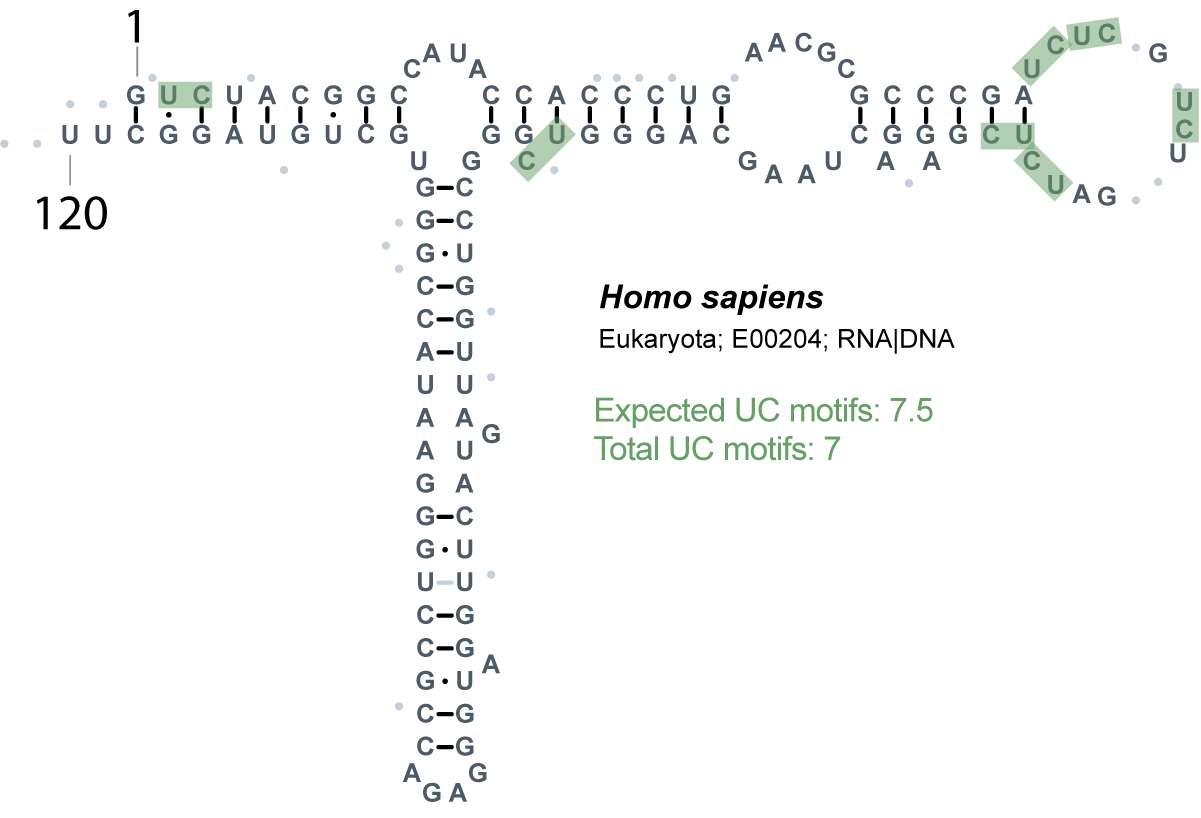

Supplement: S9 Fig — Secondary structure of the human 5S rRNA (E00204) obtained from 5SrRNAdb [87]. UC sequence motifs highlighted in green. Expected number of UC sequence motifs reported based on occurrence at random of 6.25 per 100 nucleotides. (TIF) [file pbio.3002718.s009.tif]

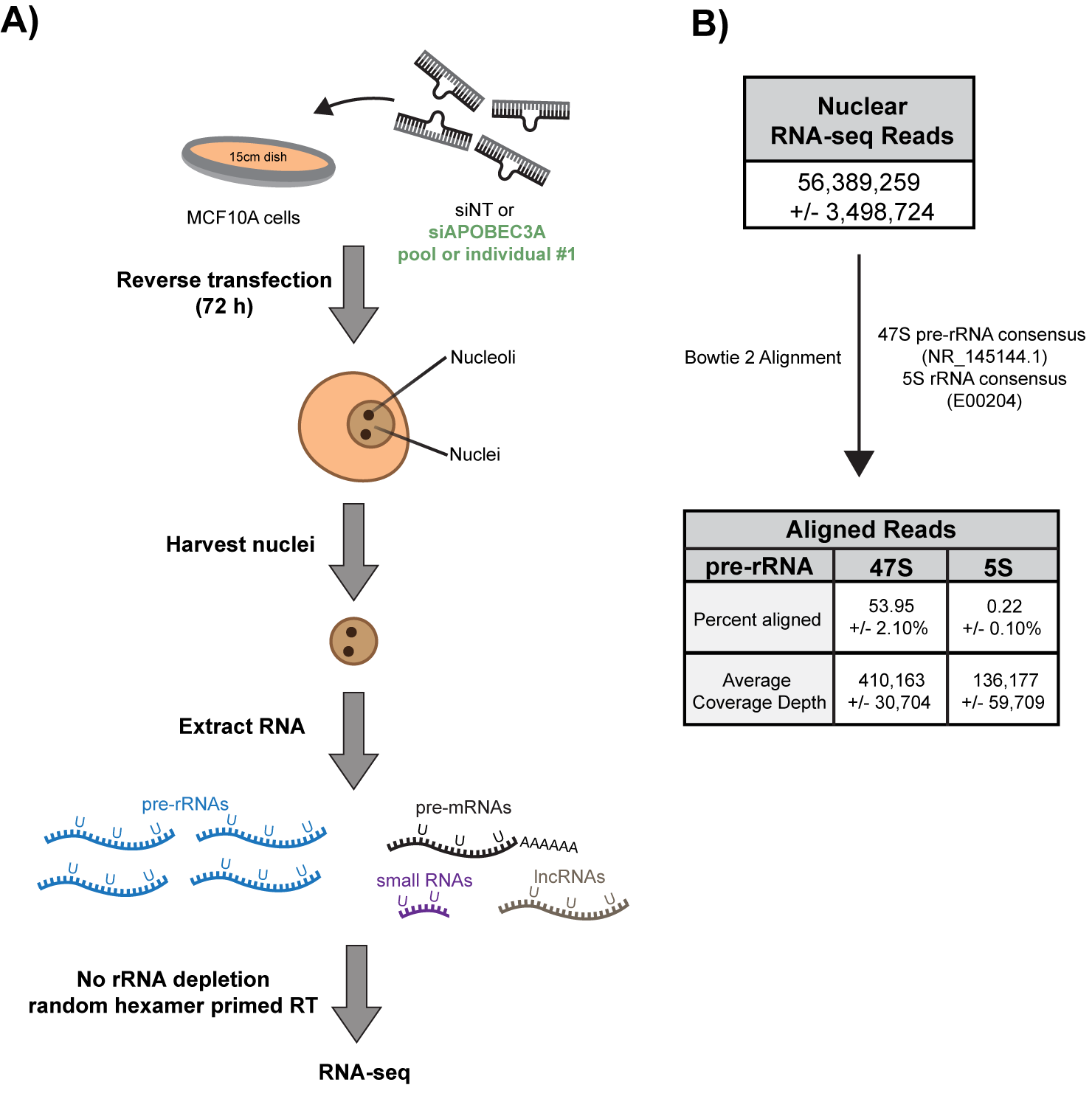

Supplement: S10 Fig — (A) Schematic of nuclear RNA-seq experiment. MCF10A cells were treated with siNT negative control, siAPOBEC3A pool, or siAPOBEC3A individual #1 for 72 h. Nuclear RNA was extracted and submitted for sequencing with no rRNA depletion step and primed with random hexamers for reverse transcription (RT). (B) Over half of the nuclear RNA-seq reads aligned to the 47S pre-rRNA and to the 5S rRNA. Average reads, percent alignment, and coverage depth of all nuclear RNA-sequencing runs using Bowtie 2 alignment to either the 47S pre-rRNA (NR_145144.1) or 5S rRNA (E00204, [87]). Average of 6 biological replicates (2x siNT, 2x siAPOBEC3A pool, 2x siAPOBEC3A #1) ± SD. (TIF) [file pbio.3002718.s010.tif]

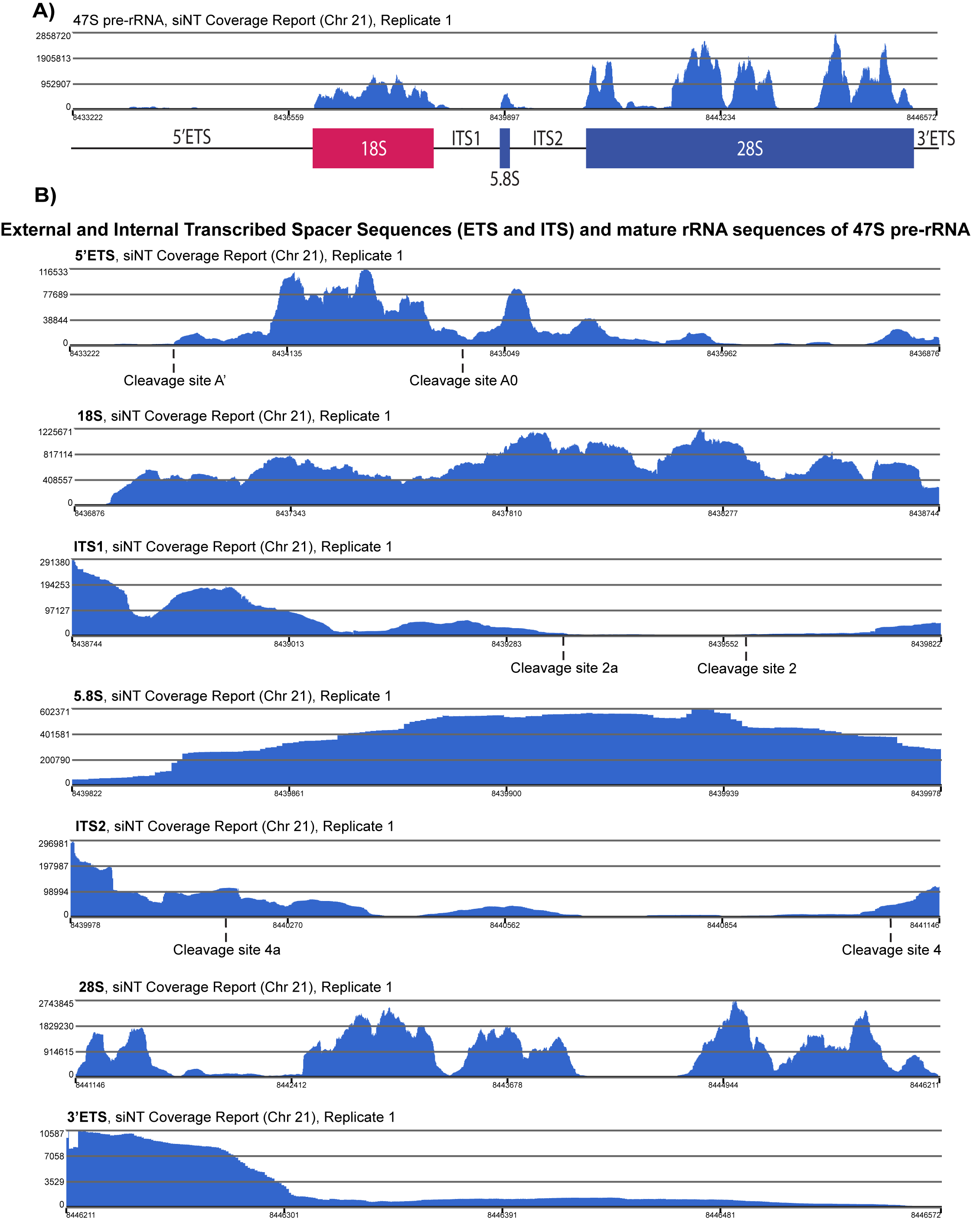

Supplement: S11 Fig — (A) Coverage report across the 47S pre-rRNA sequence (hg38, chromosome 21) after STAR (Version 2.7.8a) alignment to hg38. Y-axis is average read depth; x-axis is genomic coordinates on chromosome 21 corresponding to the location of the 47S pre-rRNA (RNA45SN1). Data is for nuclear RNA-seq of siNT negative control treated MCF10A cells replicate #1. Image was generated using Partek Flow chromosome viewer. (B) Same as in (A) except zoomed into the different portions of the 47S pre-rRNA transcript including the 5′ and 3′ external transcribed spacers (ETSs), internal transcribed spacers (ITSs) 1 and 2, and the mature (18S, 5.8S, and 28S) rRNAs. Cleavage sites indicated below the x-axis. (TIF) [file pbio.3002718.s011.tif]

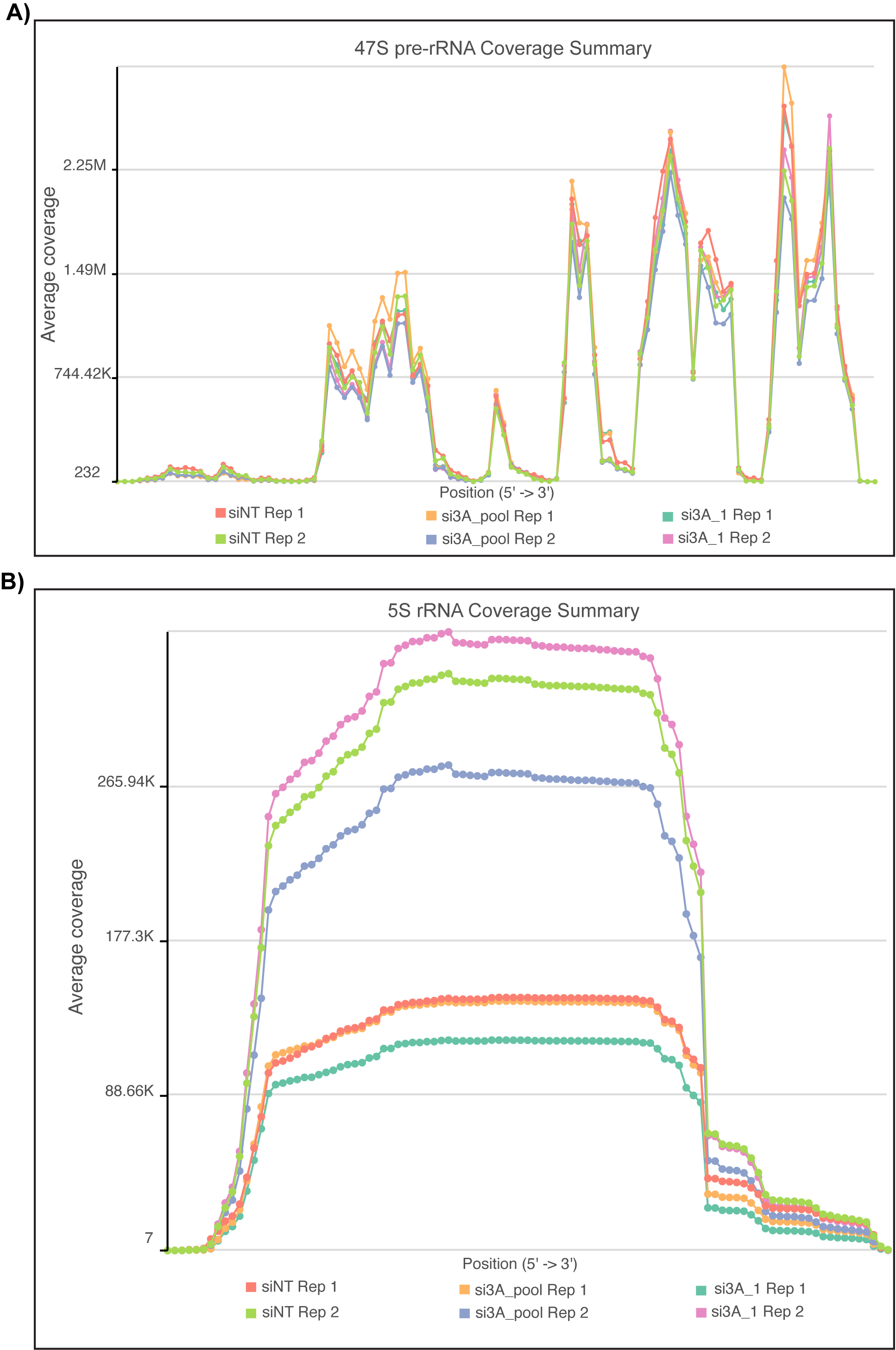

Supplement: S12 Fig — (A) Coverage summary of MCF10A cell nuclear RNA-seq reads aligned to a 47S pre-rRNA consensus sequence (NR_145144.1) for all replicates indicated by color. Y-axis is average coverage depth; x-axis is the entire 47S pre-rRNA sequence. Image was generated using Partek Flow chromosome viewer. (B) Same as in (A) except for the 5S rRNA (E00204 [87]) sequence. (TIF) [file pbio.3002718.s012.tif]

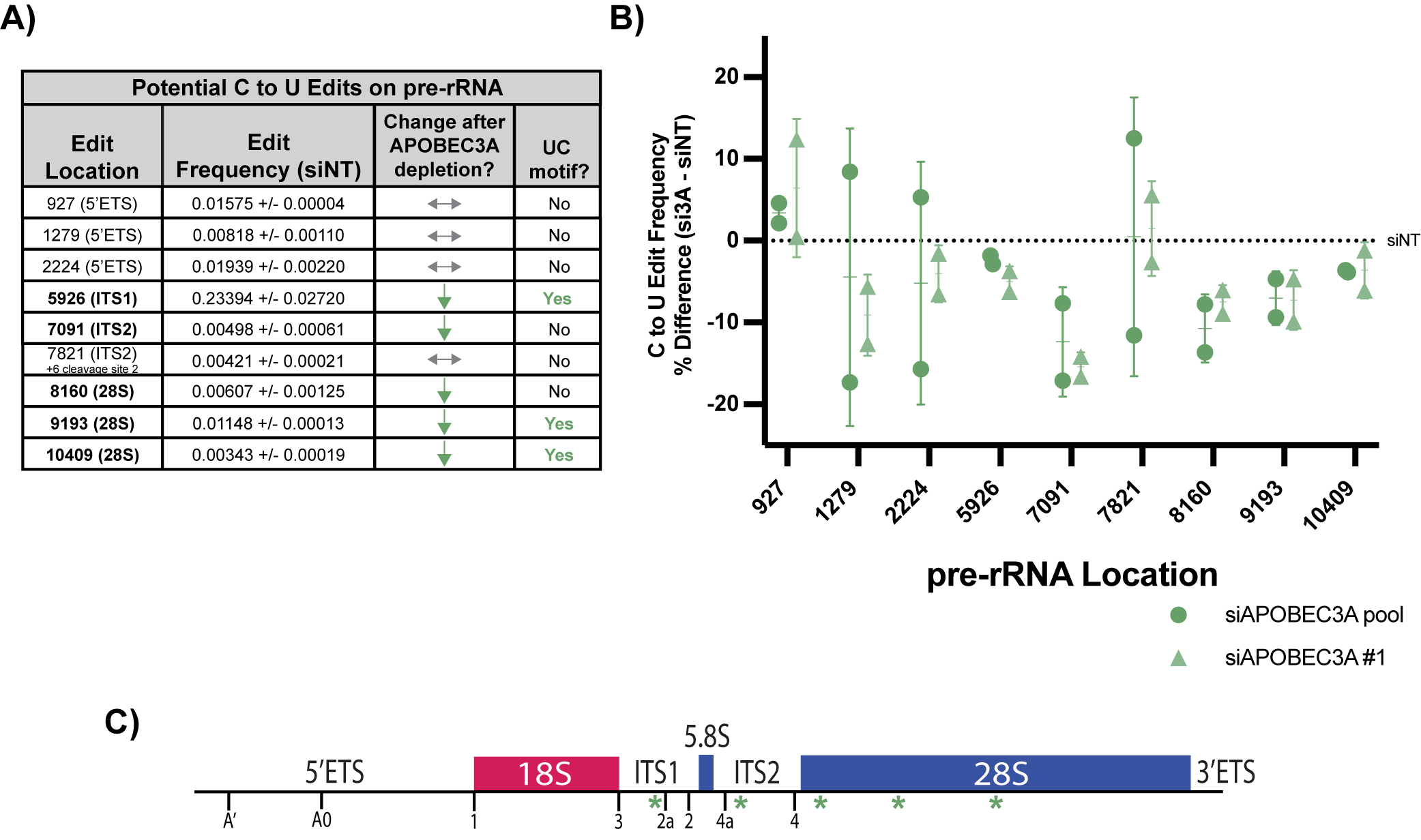

Supplement: S13 Fig — (A) LoFreq variant analysis of the nuclear RNA-seq datasets identified 5 predicted C-to-U APOBEC3A target sites in the pre-rRNA (green down arrows). Three of these occur at a UC sequence motif (green highlighted squares). Table of 9 C-to-U edit locations on the pre-rRNA detected by LoFreq (see Methods) in siNT negative control. Editing frequencies are the mean ± SD of 2 biological replicates. A change in the edit frequency after siAPOBEC3A depletion was called if there was a decrease in editing after siAPOBEC3A pool and siAPOBEC3A #1 depletion in both replicates for each (down green arrow). If not, it was indicated as a non-APOBEC3A target site (horizontal gray arrows). (B) siAPOBEC3A depletion leads to modest but reproducible decreases in editing frequency at 5 predicted C-to-U variant sites on the pre-rRNA. Percent differences between siAPOBEC3A edit frequency and siNT negative control edit frequency (dotted horizontal line) at C-to-U predicted target sites in (A) are graphed. Two biological replicates of siAPOBEC3A pool (circle data points) and siAPOBEC3A #1 (triangle data points) are plotted as the mean (horizontal line) ± SD. Locations on the pre-rRNA in nucleotides are indicated on x-axis. (C) Schematic of 47S pre-rRNA, 5 green stars indicate locations of predicted APOBEC3A C to U target sites from (A, B). All underlying numerical values for figure found in S2 Data. (TIF) [file pbio.3002718.s013.tif]

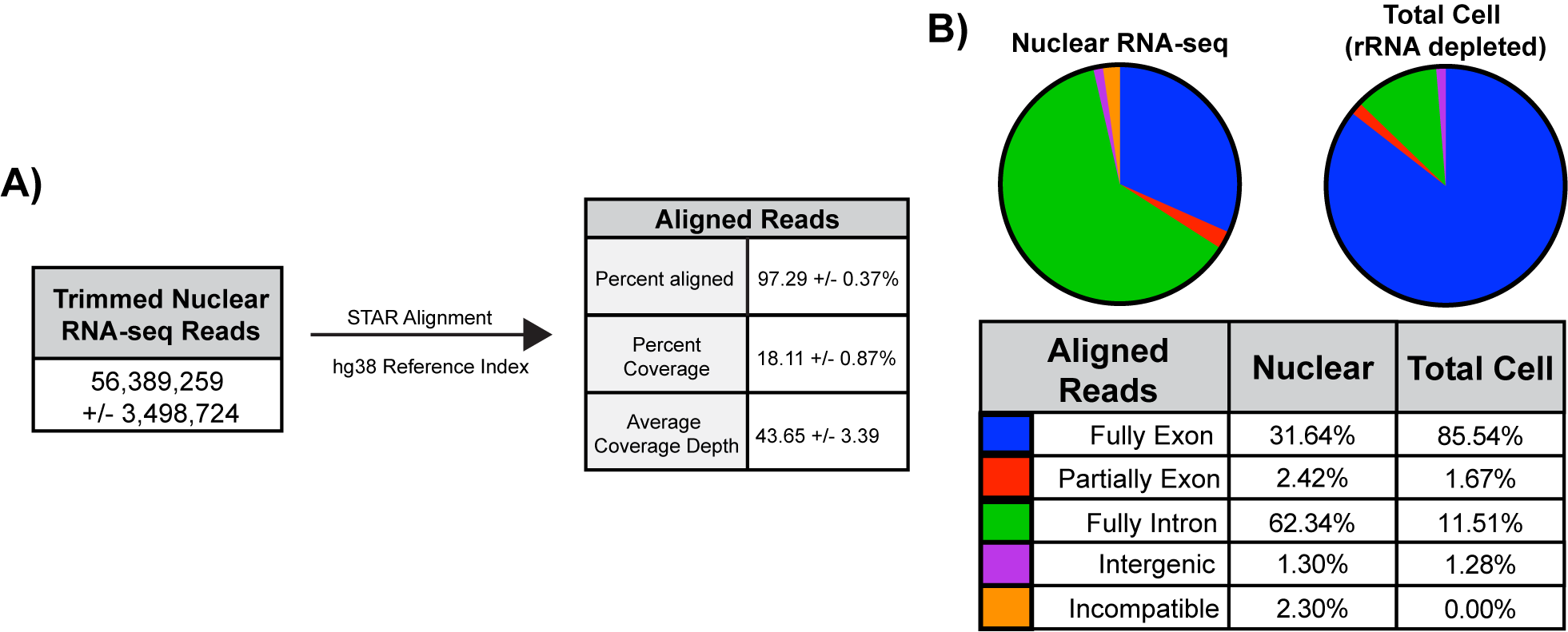

Supplement: S14 Fig — (A) Average reads, percent alignment, percent coverage percentage, and coverage depth of all nuclear RNA-sequencing runs using STAR alignment to the hg38 reference index. Average of 6 biological replicates (2x siNT negative control, 2x siAPOBEC3A pool, 2x siAPOBEC3A #1) ± SD. (B) Nuclear RNA-seq reads largely align to introns of pre-mRNAs. Exon and intron distribution of MCF10A nuclear RNA-seq read alignments (this experiment) vs. total RNA-seq (rRNA depleted) read alignment (GEO accession GSE154764) [6]. Percentage of total for each location type is shown in a pie chart and in a table based on color. (TIF) [file pbio.3002718.s014.tif]

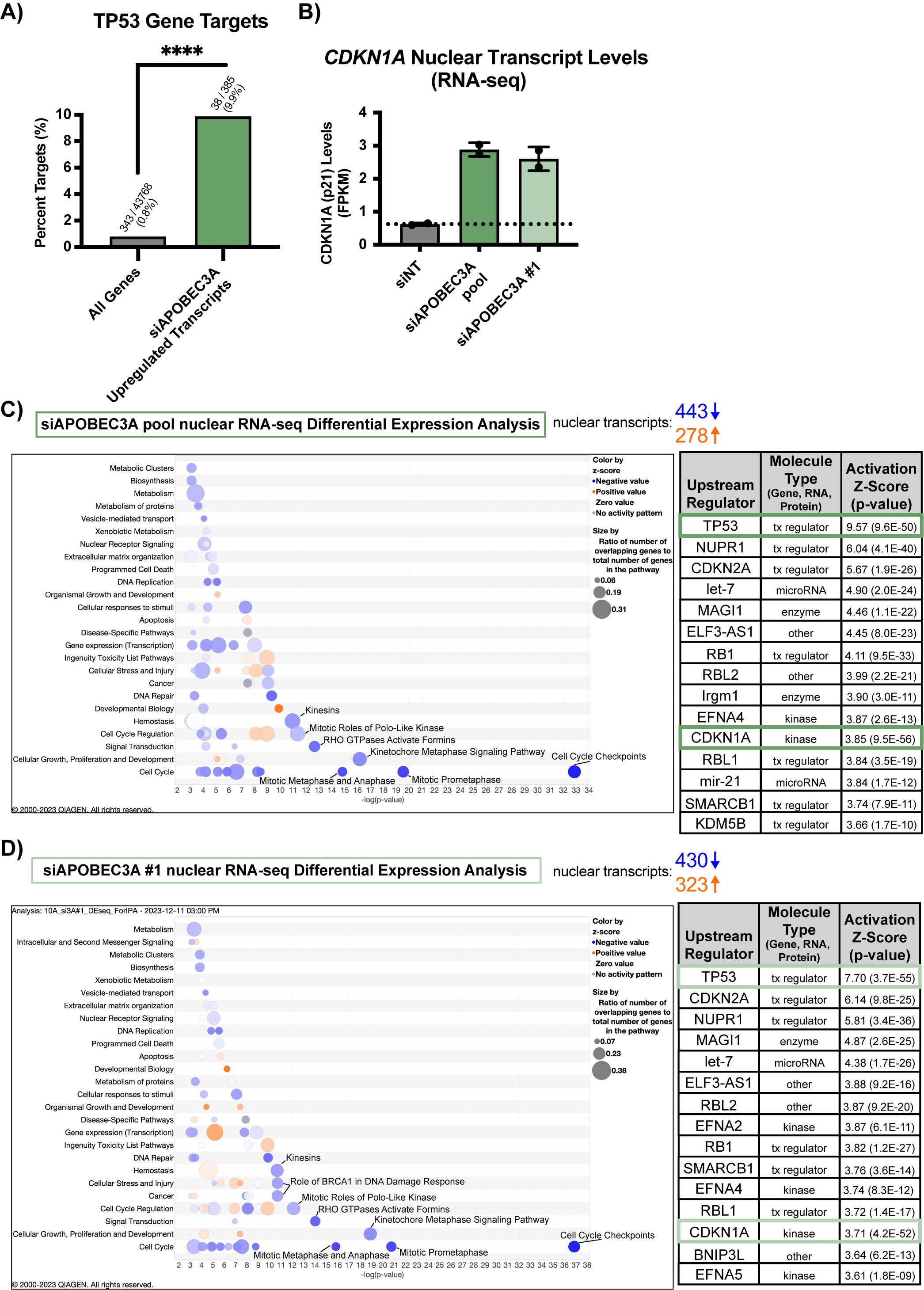

Supplement: S15 Fig — (A) siAPOBEC3A pool and siAPOBEC3A #1 treatment induced nuclear transcripts are enriched for TP53 gene target transcripts by RNA-seq, and 343 genes were determined to be high confidence targets of TP53 through analysis of several datasets [90] out of 43,768 HGNC approved human genes [113], 0.8%. siAPOBEC3A pool treatment up-regulated 29 TP53 target nuclear transcripts (>2-fold up-regulated, p ≤ 0.05, FDR ≤0.05) out of 278 total up-regulated transcripts. siAPOBEC3A #1 treatment up-regulated 41 TP53 target transcripts (>2-fold up-regulated, p ≤ 0.05, FDR ≤0.05) out of 323 total up-regulated nuclear transcripts. Percent of TP53 targets present in genome, siAPOBEC3A pool up-regulated nuclear transcripts and siAPOBEC3A #1 up-regulated nuclear transcripts are graphed. Data were analyzed by Fisher’s exact test, **** p ≤ 0.0001. (B) siAPOBEC3A treatment increases CDKN1A nuclear transcript levels in MCF10A cells by RNA-seq. CDKN1A FPKM for 2 biological replicates of negative control siNT, siAPOBEC3A pool, and siAPOBEC3A #1. (C) Qiagen Ingenuity Pathway Analysis of siAPOBEC3A pool differentially expressed nuclear transcripts, and 443 down-regulated transcripts (>2-fold, p ≤ 0.05, FDR ≤0.05, blue) and 278 up-regulated transcripts (>2-fold, p ≤ 0.05, FDR ≤0.05, orange). (Left) Chart of enriched pathways where -log(p-value) > 3.0. Top 7 pathways are labeled. Size of circle represents ratio of overlapping genes represented in dataset. Up-regulated pathways are in orange and down-regulated pathways are in blue where darkness of shade indicates the extent to which pathway is regulated. (Right) Table of top 15 positive upstream regulators enriched in up-regulated transcript list. TP53 and CDKN1A are bolded in green. Molecule type and (positive) Z-score shown. (D) Same as in (C) except for siAPOBEC3A #1 treatment, and 440 down-regulated transcripts (>2-fold, p ≤ 0.05, FDR ≤0.05, blue) and 323 up-regulated transcripts (>2-fold, p ≤ 0.05, FDR ≤0.05, orange). All underlying numerical va [file pbio.3002718.s015.tif]

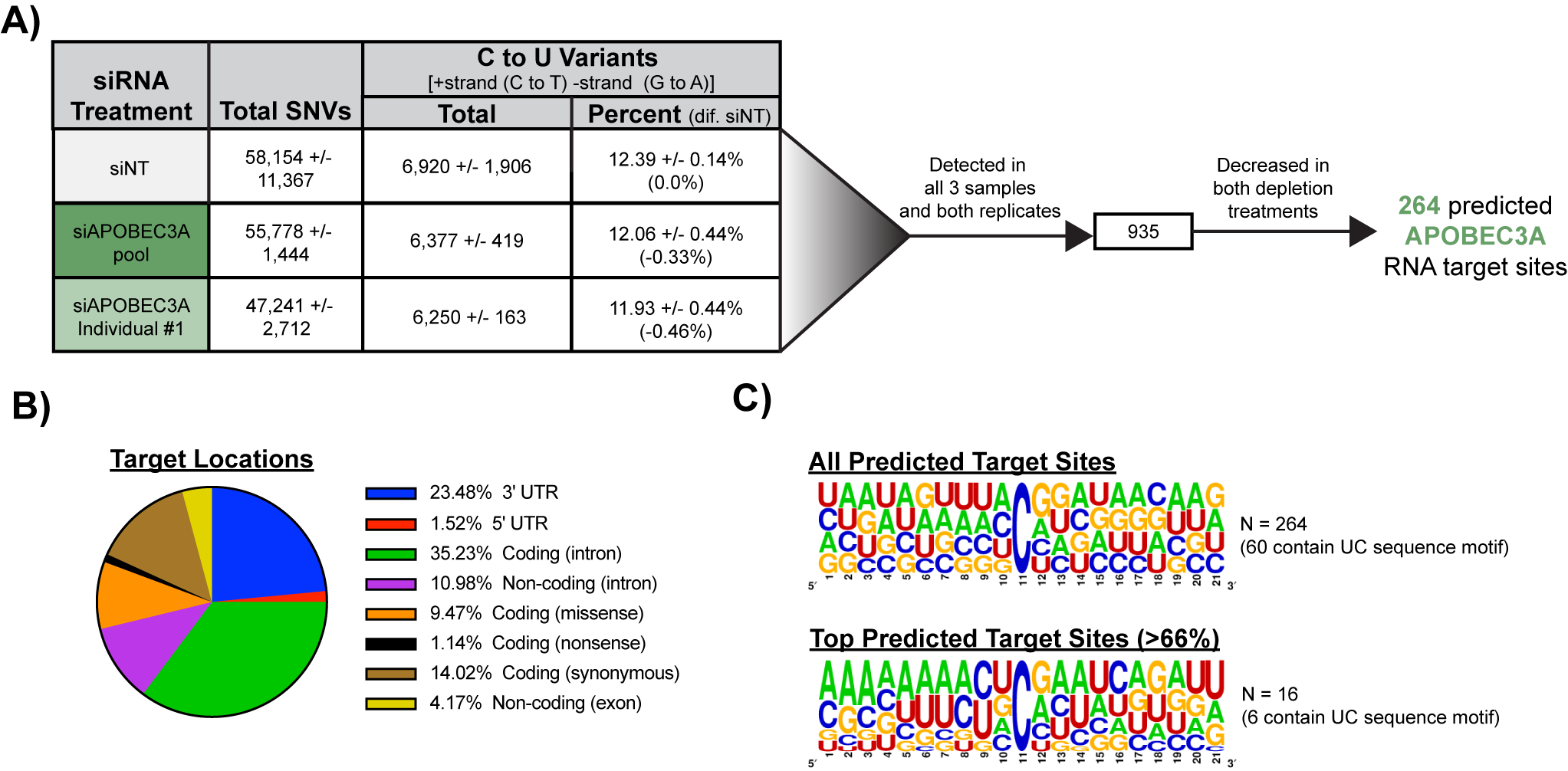

Supplement: S16 Fig — (A) There are 264 predicted APOBEC3A C-to-U edit sites that were revealed by LoFreq variant analysis of nuclear RNAs. The total number of all single-nucleotide variants (SNVs) reported by LoFreq was reported as an average of 2 biological replicates ± SD. Total and percent of total C-to-U variants were reported as an average of 2 biological replicates ± SD (percent difference from siNT negative control). C-to-U variants were filtered for those that were detected in both replicates of all 3 samples (i.e., all 6 biological replicates) and those that decreased on average for both siAPOBEC3A pool and siAPOBEC3A #1 depletions. (B) APOBEC3A predicted target sites on nuclear RNAs are found mostly within intronic regions. The distribution of the 264 predicted APOBEC3A target sites in (A) location types are reported as a percentage of total in a pie chart. Locations which include 5′ or 3′ untranslated regions (UTRs), transcript types (coding or non-coding), and edit type in parentheses (intron, missense, nonsense, synonymous, non-coding exon) are indicated by color. (C) The APOBEC3A UC sequence motif is not significantly enriched in the top predicted target sites. (Top) sequence logos for all 264 predicted target sites in (A) including 10 nucleotides upstream and downstream of the edited C. (Bottom) Same as (Top) except indicating only the top 16 predicted target sites (exhibiting a greater than 66% decrease in siNT editing frequency compared to the average of the siAPOBEC3A pool and siAPOBEC3A #1 treatments) were used for logo creation. (TIF) [file pbio.3002718.s016.tif]

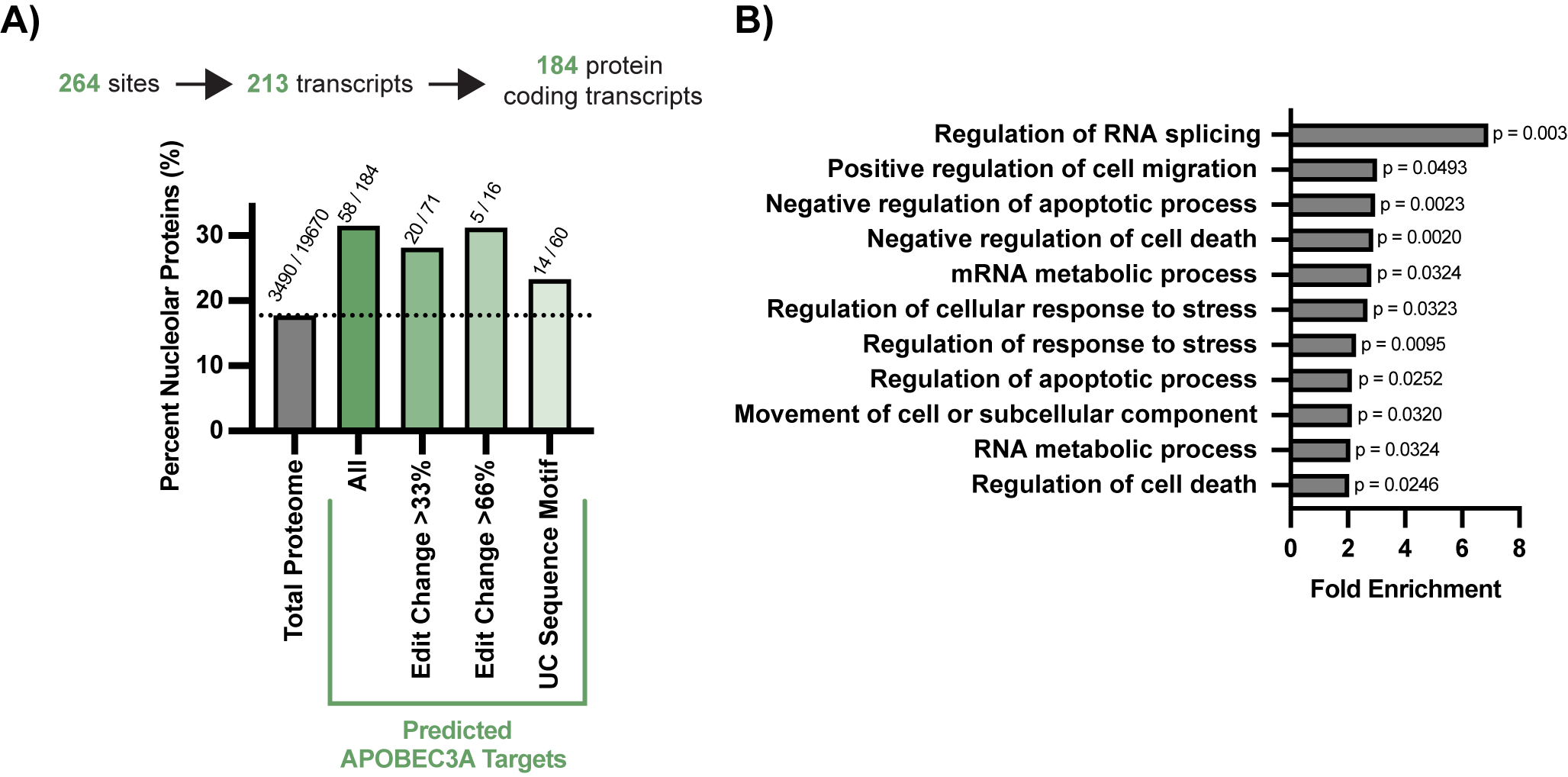

Supplement: S17 Fig — (A) The APOBEC3A predicted target sites are enriched on pre-mRNAs encoding nucleolar proteins. The 264 predicted target sites were located on 213 transcripts of which 184 are protein coding. An estimate of human nucleolar proteins were determined based on the presence of a protein in at least 1 of 3 proteomic datasets (N = 3,490) [91–94]. The total number of human proteins was reported based on Thul and colleagues (N = 19,670) [94]. Proteins that were encoded by transcripts with APOBEC3A predicted targets and subgroups within were tested for enrichment of nucleolar protein coding transcripts by comparison to an estimate of percentage of the proteome that is nucleolar (17.7%, dotted horizontal line) and graphed. (B) The APOBEC3A predicted target sites are enriched on pre-mRNAs involved in RNA metabolism, positive regulators of cell migration, and negative regulators of cell apoptosis/death. All 213 transcripts that contain APOBEC3A predicted target sites were analyzed for overrepresentation of biological function gene ontology categories using the STRING database [95]. Categories were reported where fold-enrichment > 2.0 and p < 0.05, and p-values indicated on graph. All underlying numerical values for figure found in S2 Data. (TIF) [file pbio.3002718.s017.tif]

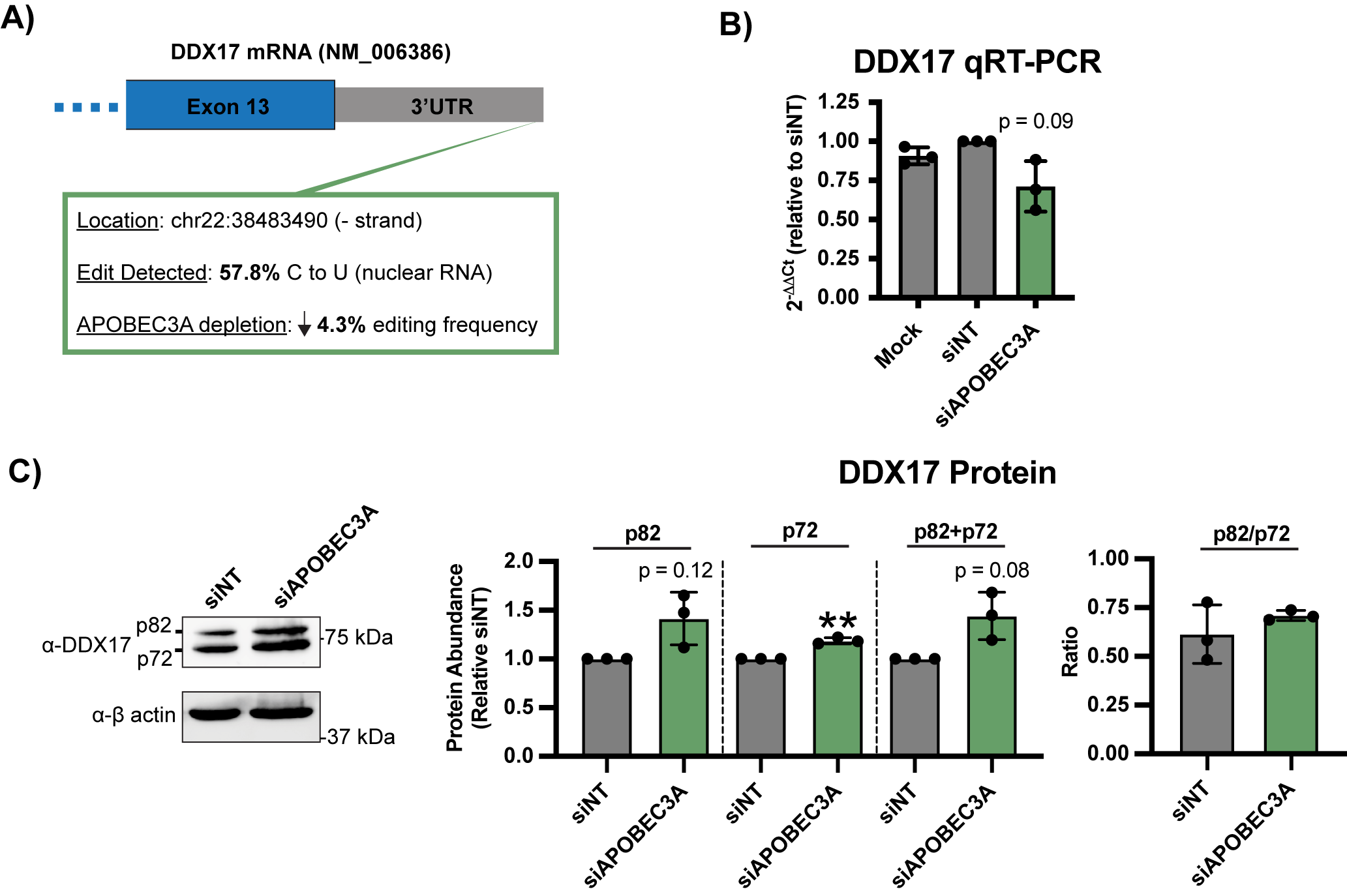

Supplement: S18 Fig — (A) DDX17 (NM_006386) contains an APOBEC3A predicted target site within its 3′ UTR. (B) siAPOBEC3A depletion (pool) does not significantly reduce DDX17 mRNA levels. qRT-PCR was performed to measure primary DDX17 mRNA levels. Mock and siNT are negative controls; 2-ΔΔCT measured relative to 7SL internal control and siNT negative control. Three technical replicates of 3 biological replicates plotted mean ± SD. Data were analyzed by Student’s t test, p-value indicated on graph. (C) siAPOBEC3A depletion (pool) does not reduce DDX17 protein levels or change its isoform ratio. (Left) Representative western blot using an α-DDX17 antibody. α-β-actin is shown as a loading control. (Right) Quantification of DDX17 (p72 and p82 isoforms) protein levels normalized to β-actin signal and relative to siNT and quantification of p82/p72 isoform ratio. Three biological replicates plotted mean ± SD. Data were analyzed by Student’s t test, ** p ≤ 0.01. All underlying numerical values for figure found in S2 Data. (TIF) [file pbio.3002718.s018.tif]
