# Supplementary material for: The cytidine deaminase APOBEC3A regulates nucleolar function to promote cell growth and ribosome biogenesis
Source: PLoS Biol. 2024 Jul 8;22(7):e3002718. doi: 10.1371/journal.pbio.3002718 (PMC11257408; doi:10.1371/journal.pbio.3002718)

Figure 1E, α-APOBEC3A/B panel: Chemiluminescence

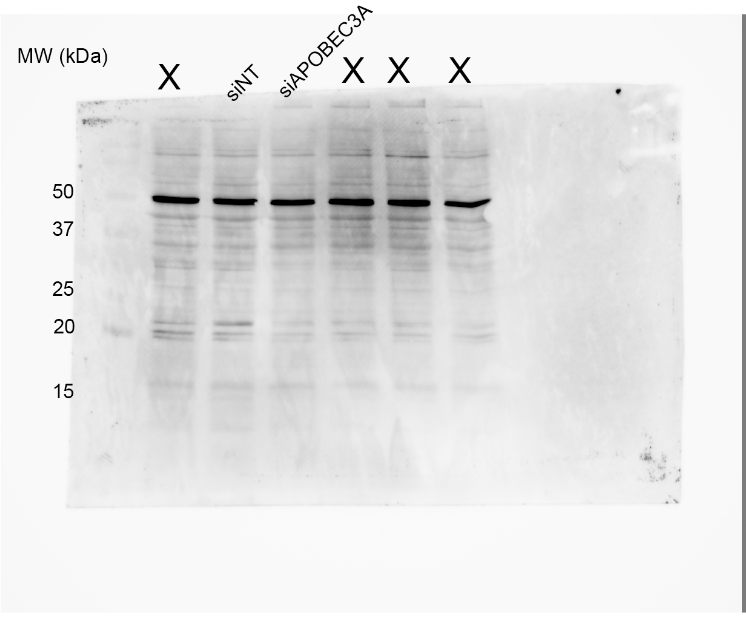

Figure 1E, α-APOBEC3A/B panel: Composite
Chemiluminescence and colormetric (for MW ladder visualization)

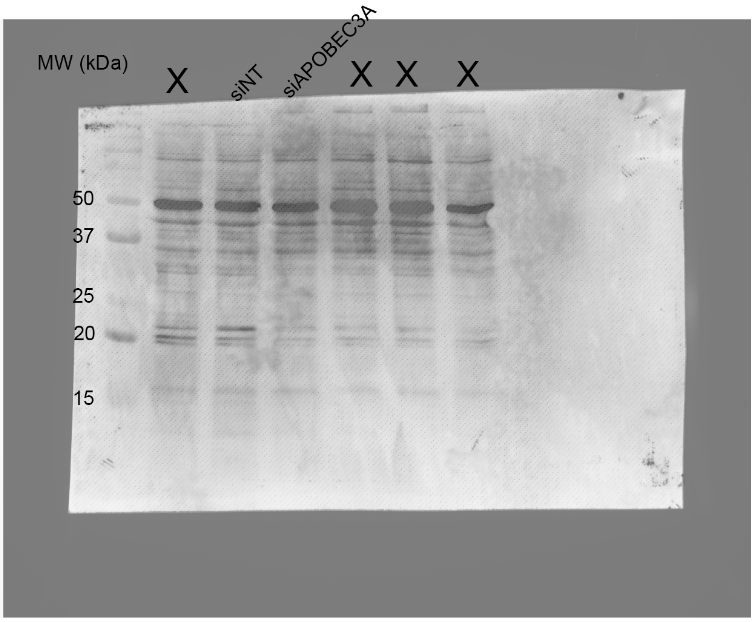

Figure 1E, α-β actin panel: Chemiluminescence

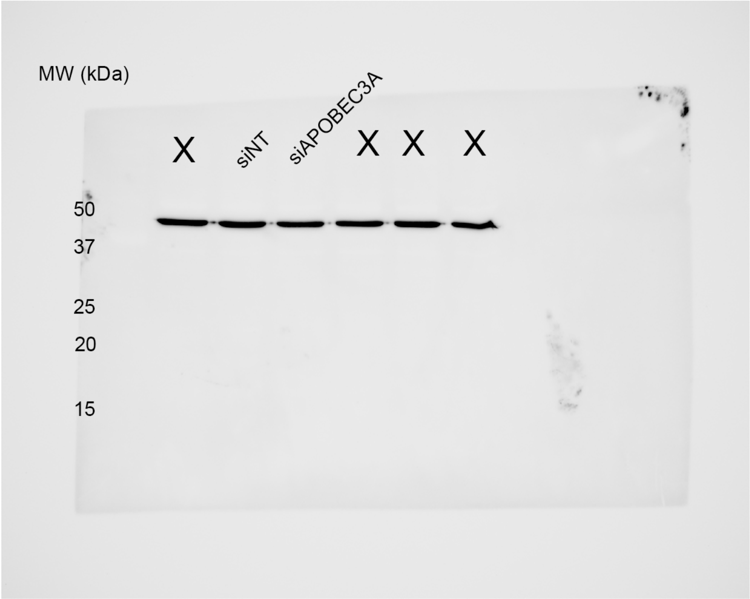

Composite α-APOBEC3A/B blot has MW markers more easily visibible for this blot (reprobed with α-β actin)

Figure 2B, α-TP53 panel: Chemiluminescence

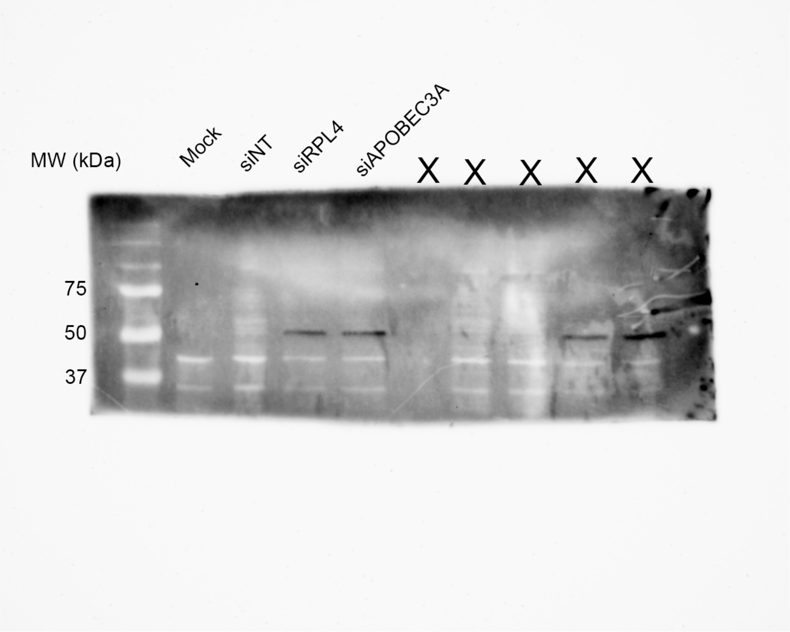

Figure 2B, α-CDKN1A (p21) panel: Chemiluminescence

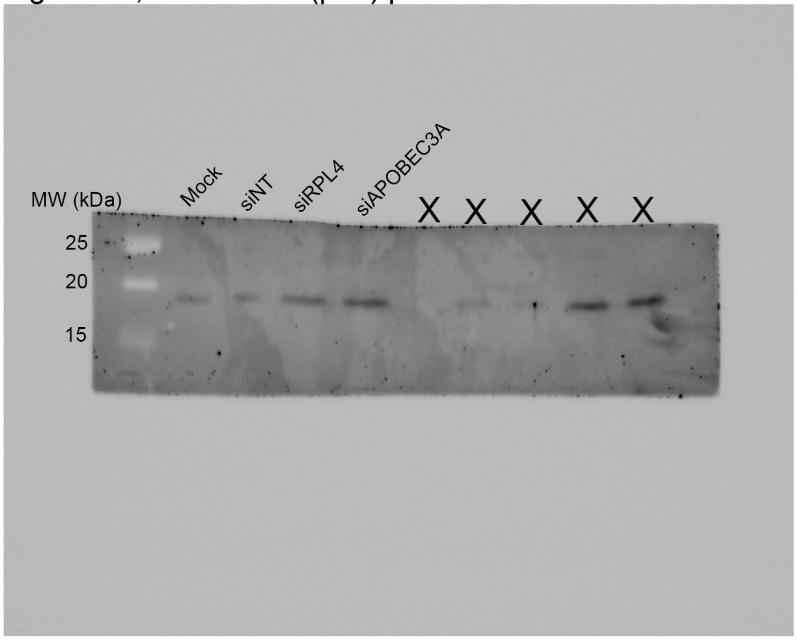

Figure 2B, α-β actin panel: Chemiluminescence

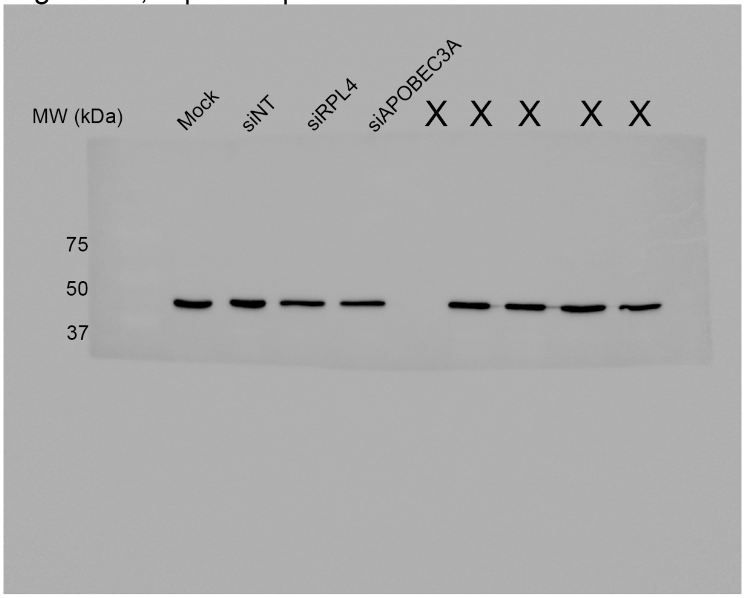

TP53 blot has MW markers more easily visibible for this blot (reprobed with α-β actin)

Figure 2C, α-puromycin panel: Chemiluminescence

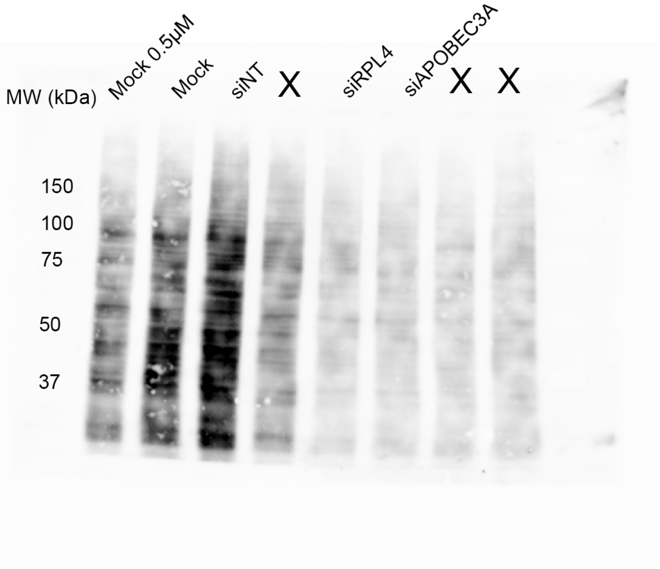

Figure 2C, α-puromycin panel: Composite
Chemiluminescence and colormetric (for MW ladder visualization)

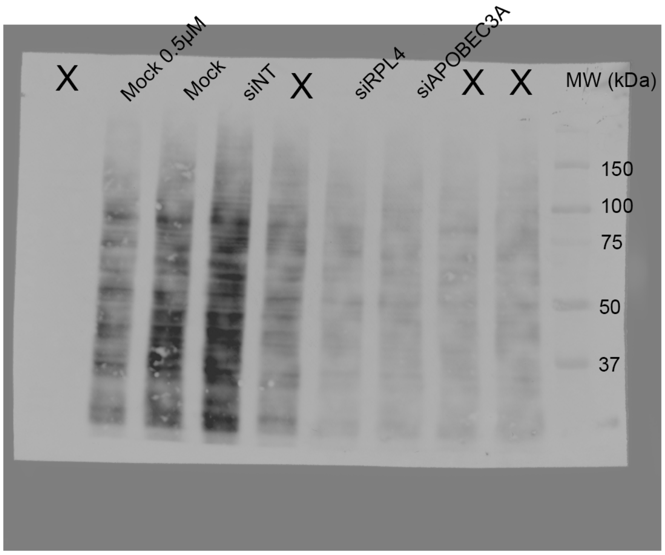

Figure 2C, α-β actin panel: Chemiluminescence

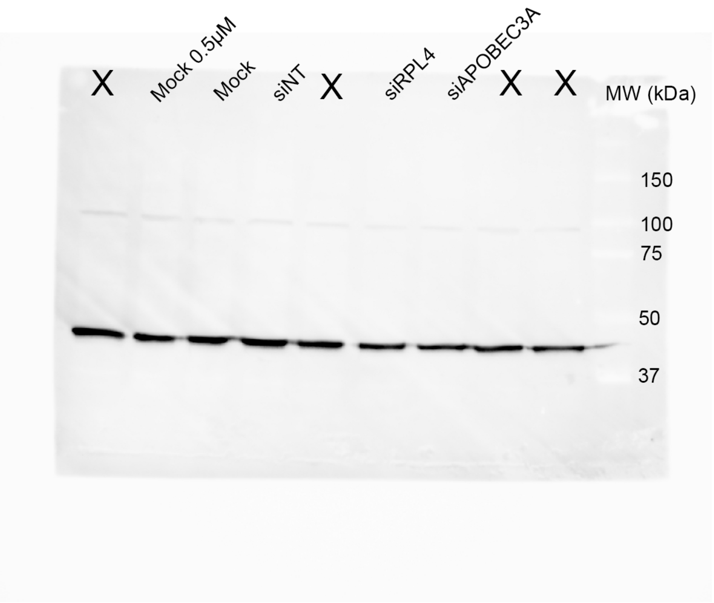

Composite α-puromycin blot has MW markers more easily visibilible for this blot (reprobed with α-β actin)

Figure 3F, Northern blot (ITS2 probe): Phosphorimage

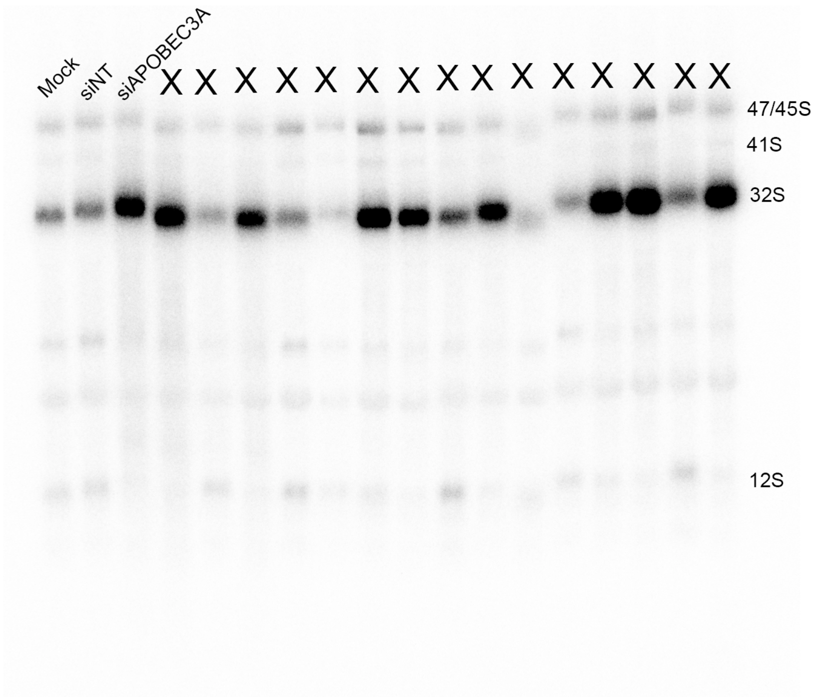

Figure 3F, Northern blot (ITS2 probe): Phosphorimage

Different exposures of same blot to allow for better visualization of to 47S/45S, 41S, and 32S above compared to 12S below

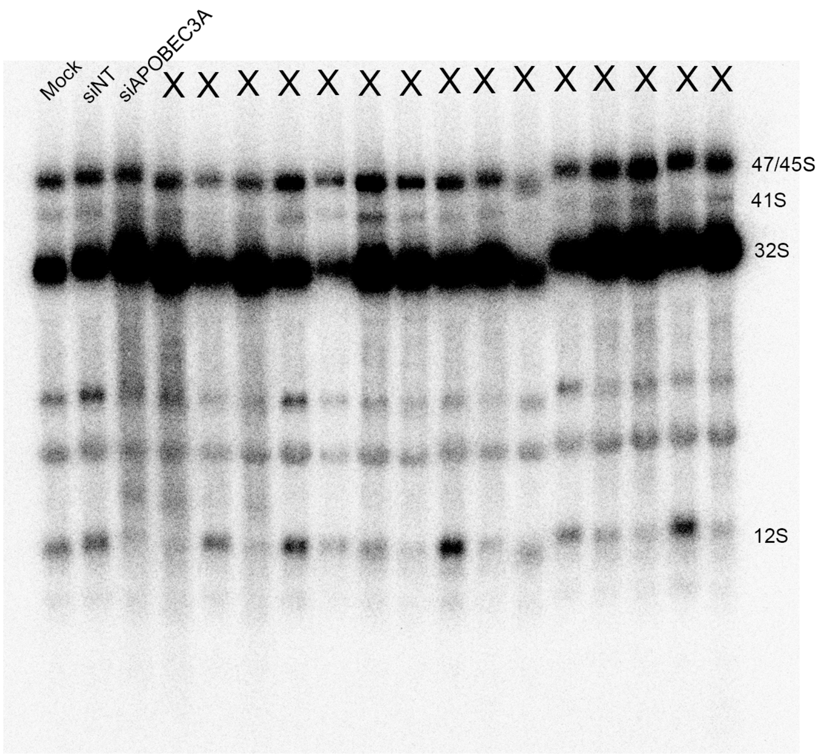

Figure 3F, Methylene blue stain: Colormetric

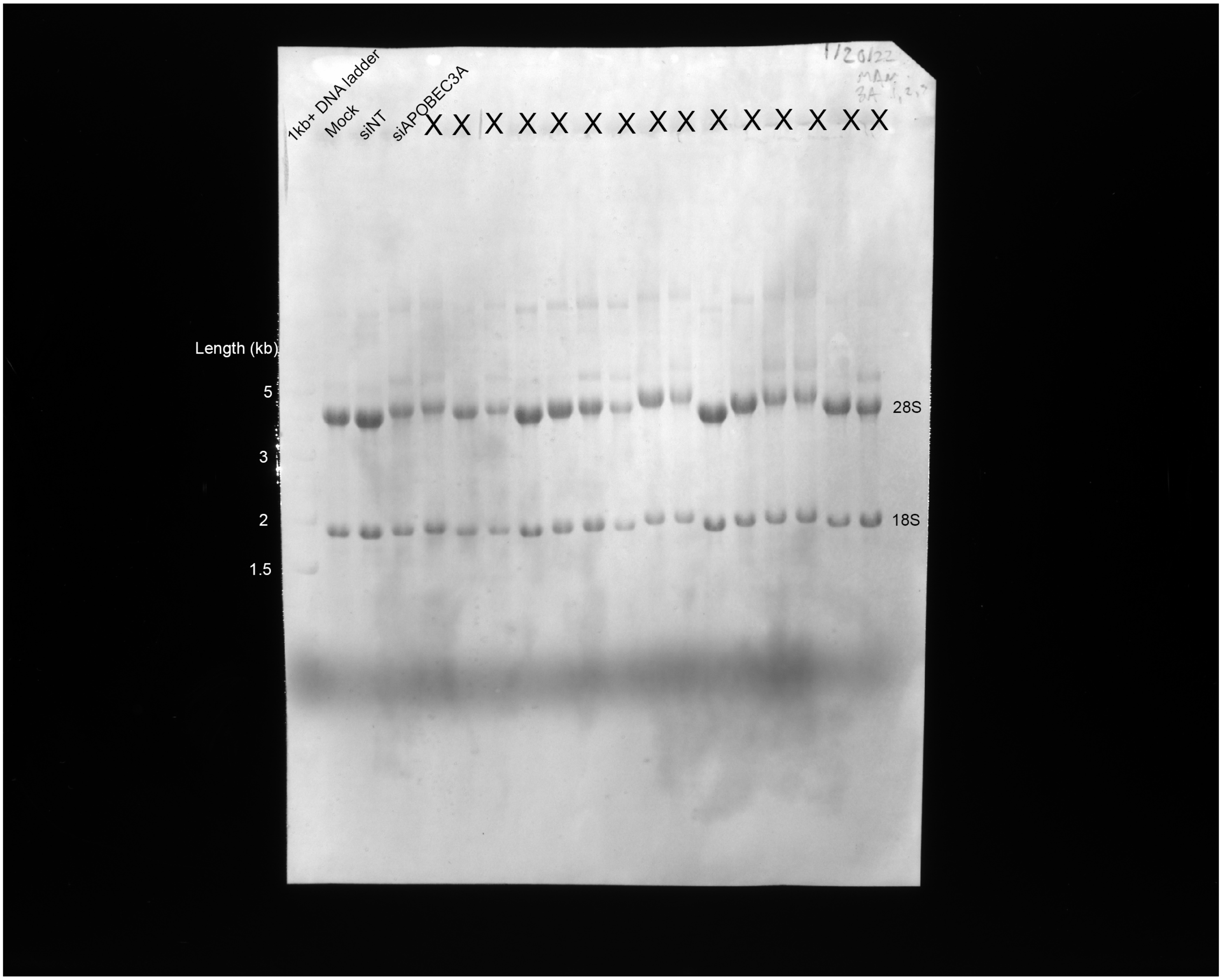

Figure 4C, α-RPA194 panel: Chemiluminescence

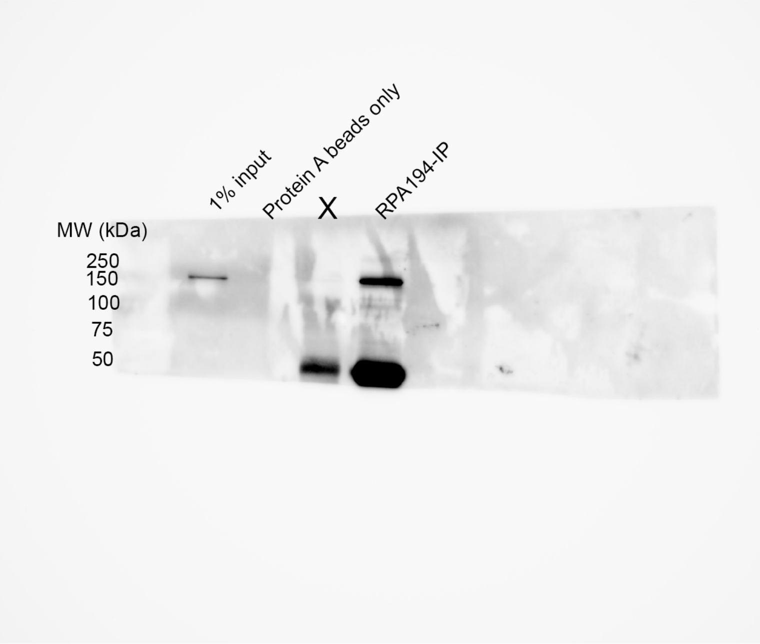

Figure 4C, α-RPA194 panel: Colormetric
(For MW ladder visualization)

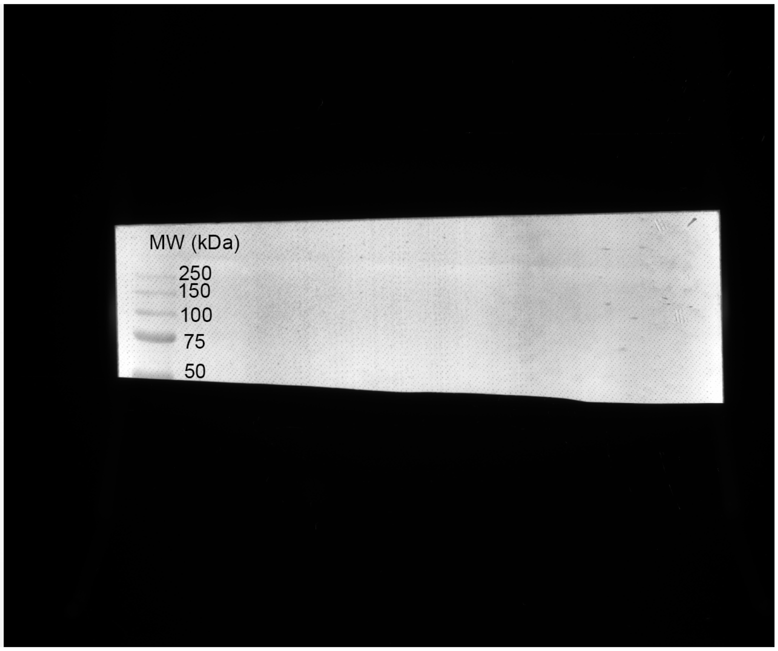

Figure 4C, α-FBL panel: Chemiluminescence

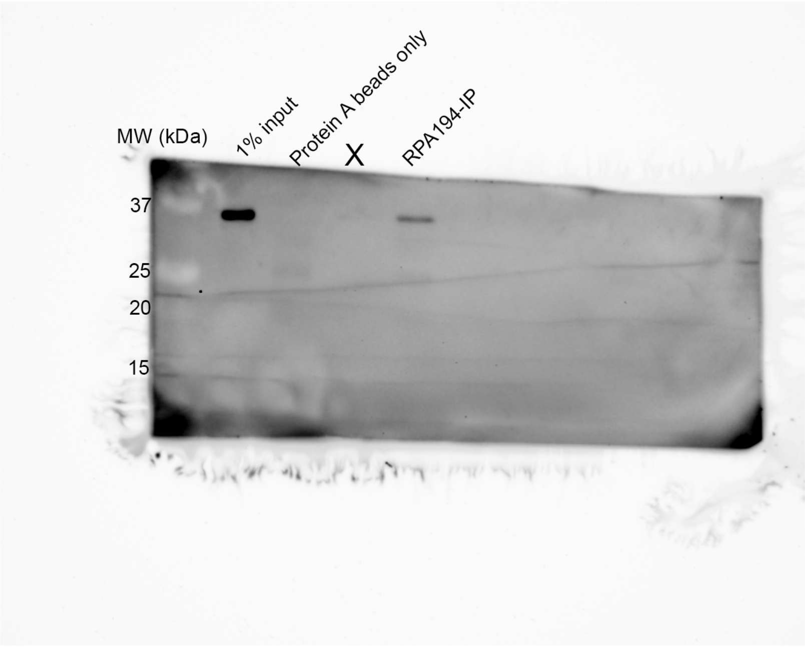

Composite α-FLAG blot has MW markers more easily visibible for this blot (reprobed with α-FBL)

Figure 4C, α-FLAG panel: Chemiluminescence

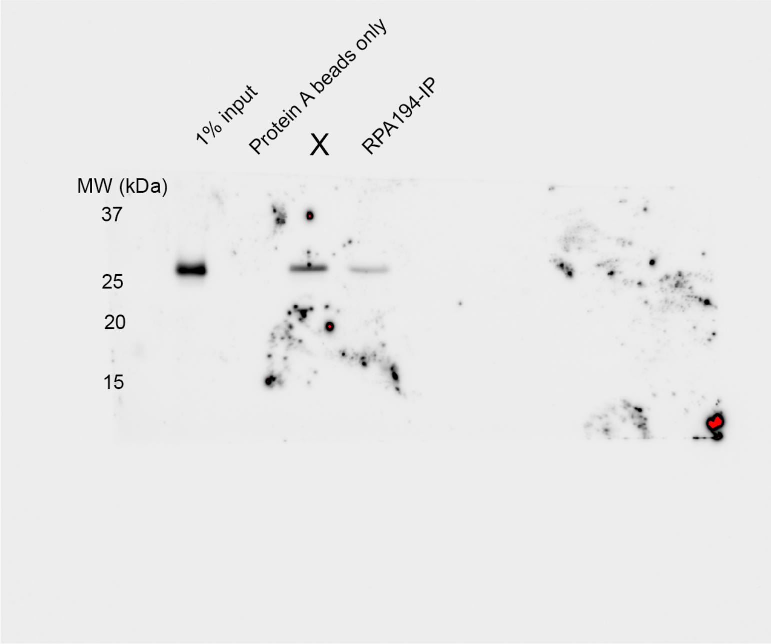

Figure 4C, α-FLAGpanel: Composite

Chemiluminescence and colormetric (for MW ladder visualization)

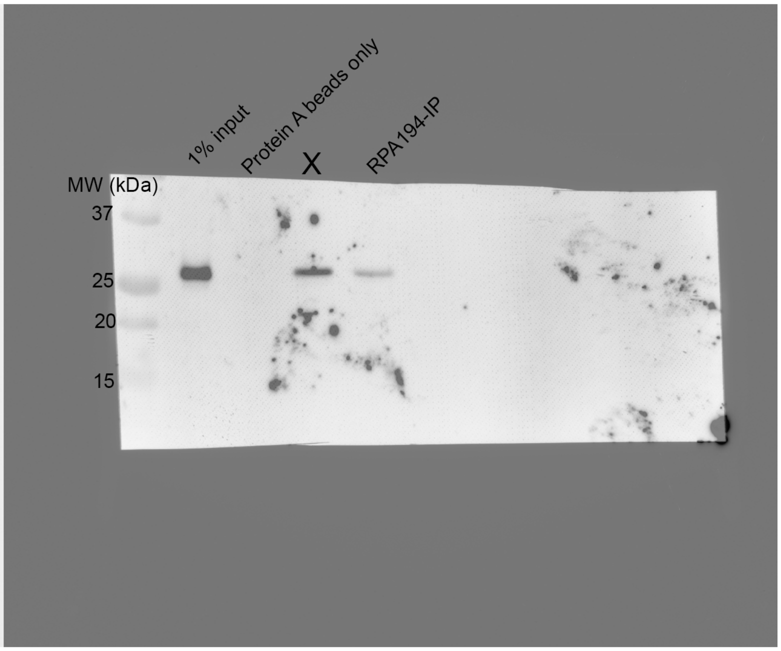

Figure 4D, α-RPA194 panel: Chemiluminescence

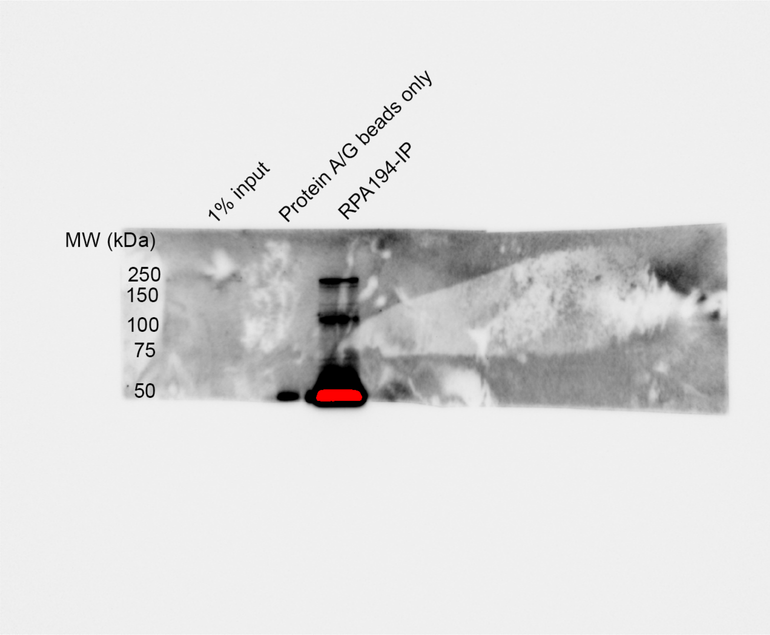

Figure 4D, α-RPA194 panel: Colormetric
(For MW ladder visualization)

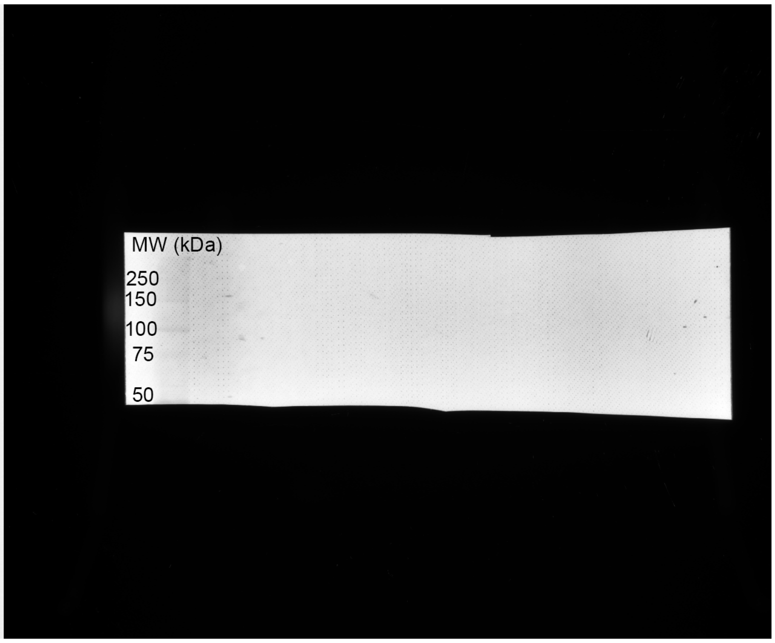

Figure 4D, α-KAP1 panel: Chemiluminescence

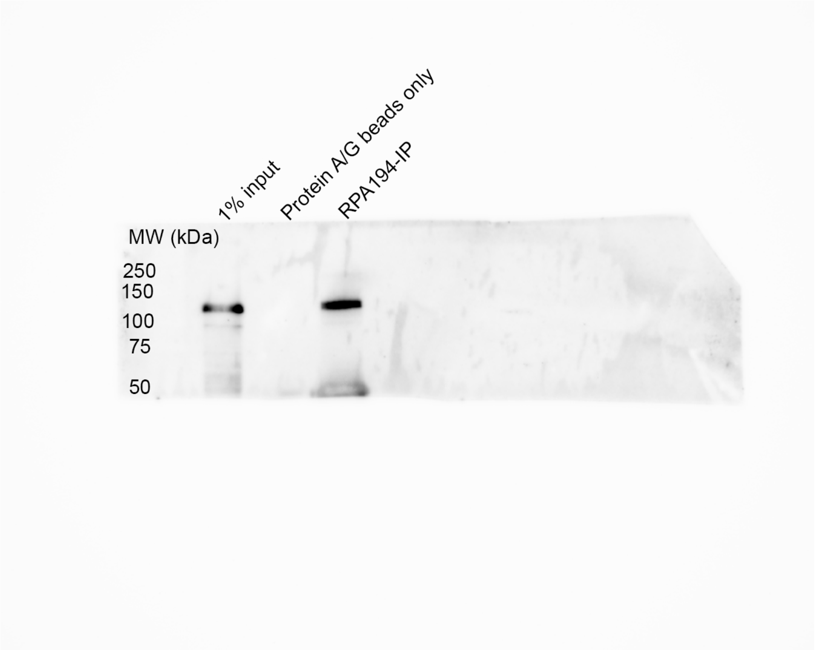

Colormetric α-RPA194 blot has MW markers more easily visibible for this blot (reprobed with α-KAP1, cut in top right corner to keep track of blot)

Figure 4D, α-PES1 panel: Chemiluminescence

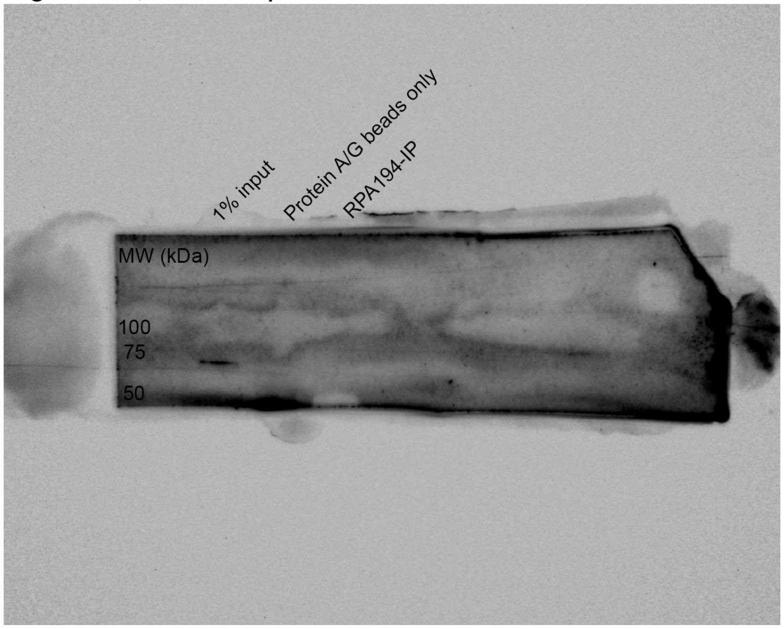

Colormetric α-RPA194 blot has MW markers more easily visibible for this blot (reprobed with α-PES1, cut in top right corner to keep track of blot)

Figure 4D, α-FLAG panel: Chemiluminescence

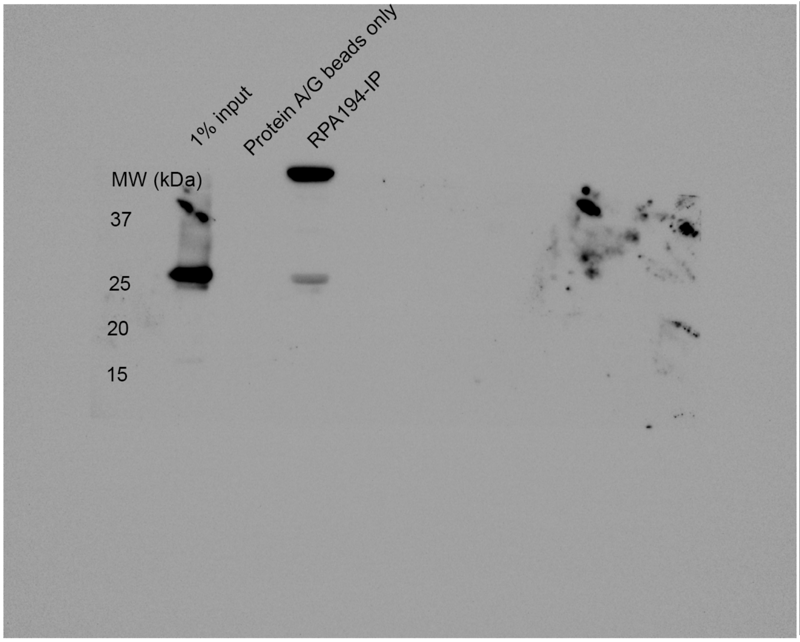

Figure 4D, α-FLAGpanel: Composite

Chemiluminescence and colormetric (for MW ladder visualization)

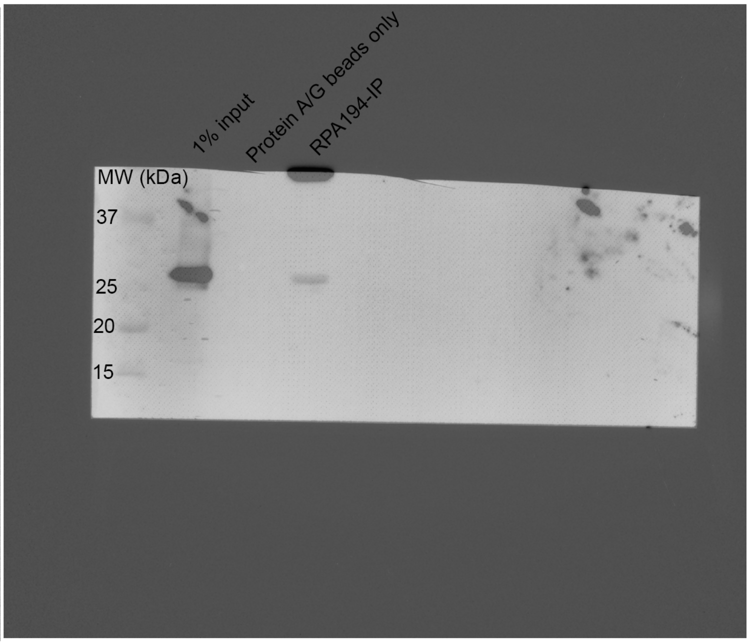

Figure 4E, α-RPA194 panel: Chemiluminescence

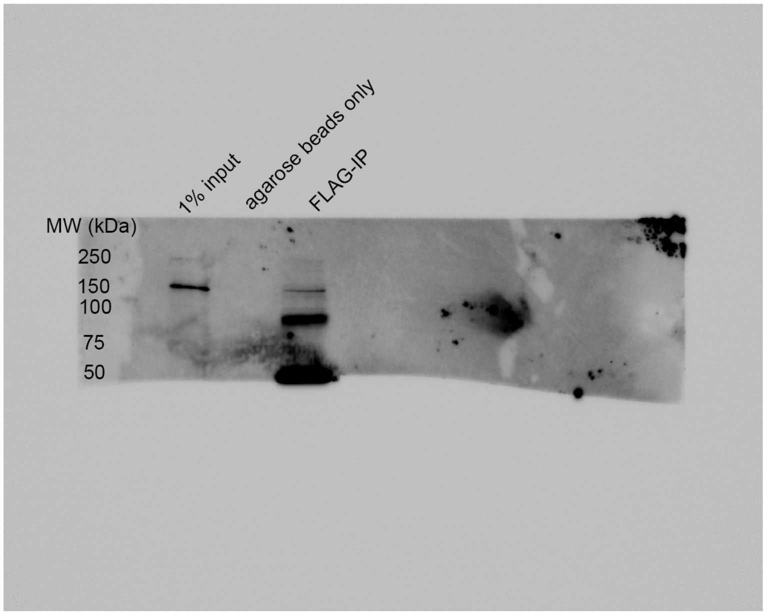

Figure 4E, α-RPA194 panel: Colormetric
(For MW ladder visualization)

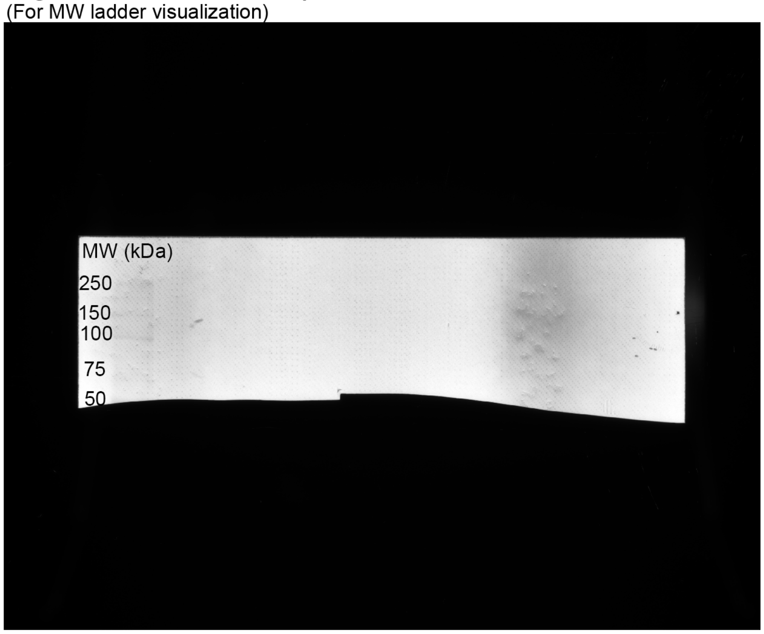

Figure 4E, α-KAP1 panel: Chemiluminescence

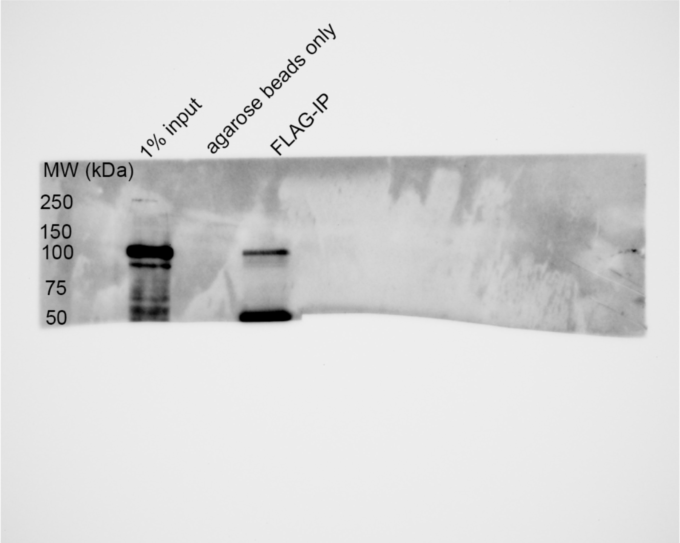

Colormetric α-RPA194 blot has MW markers more easily visibible for this blot (reprobed with α-PES1)

Figure 4E, α-PES1 panel: Chemiluminescence

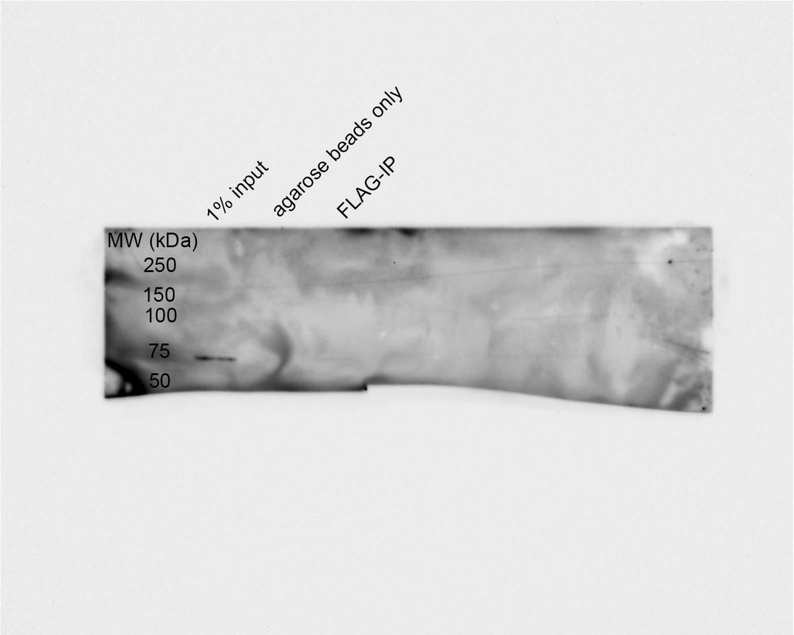

Colormetric α-RPA194 blot has MW markers more easily visibible for this blot (reprobed with α-PES1)

Figure 4E, α-FBL panel: Chemiluminescence

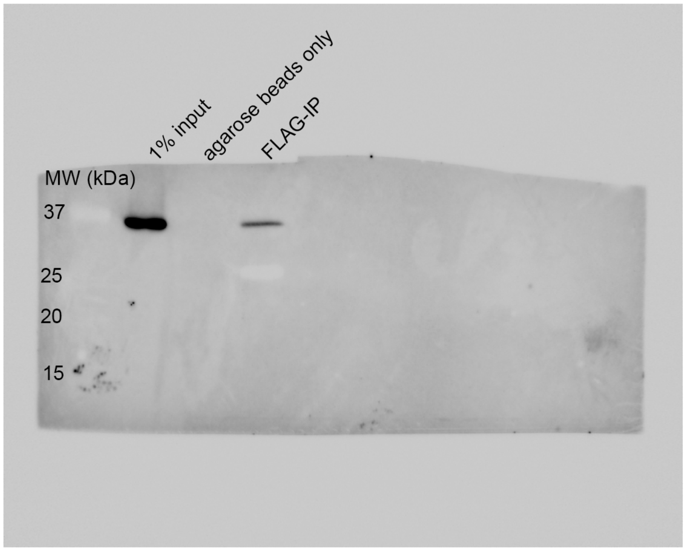

Colormetric α-FLAG blot has MW markers more easily visibible for this blot (reprobed with α-FBL)

Figure 4E, α-FLAG panel: Chemiluminescence

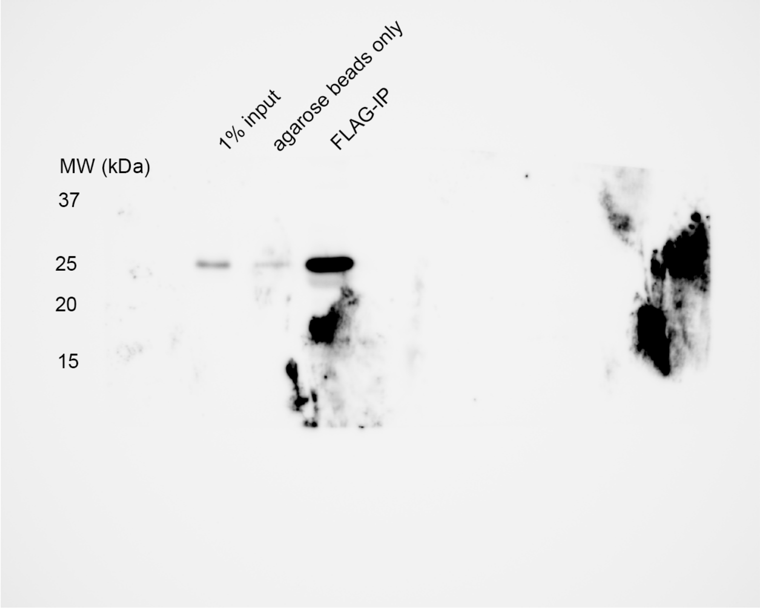

Figure 4E, α-FLAG panel: Colormetric
(For MW ladder visualization)

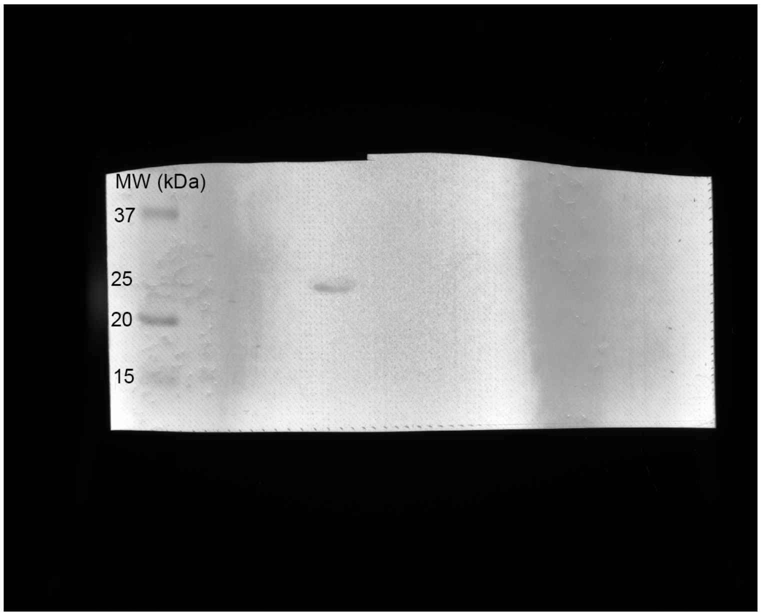

Figure 5C, α-puromycin panel: Chemiluminescence

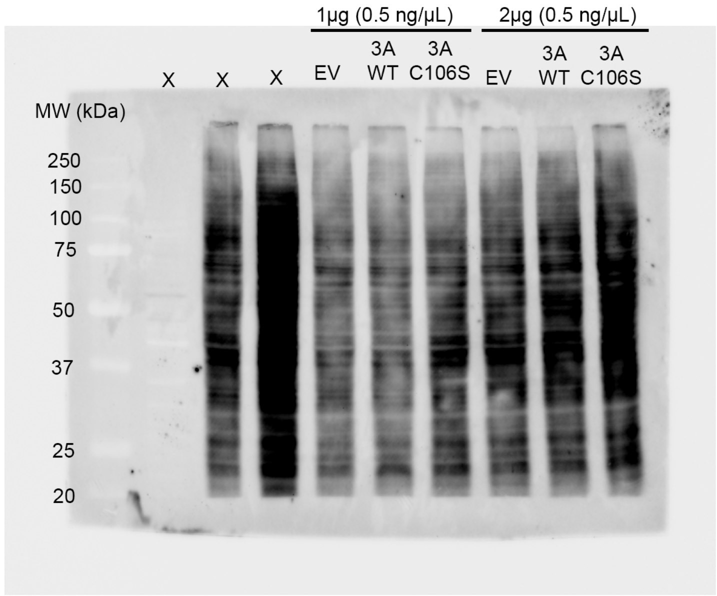

Figure 5C, α-puromycin panel: Colormetric

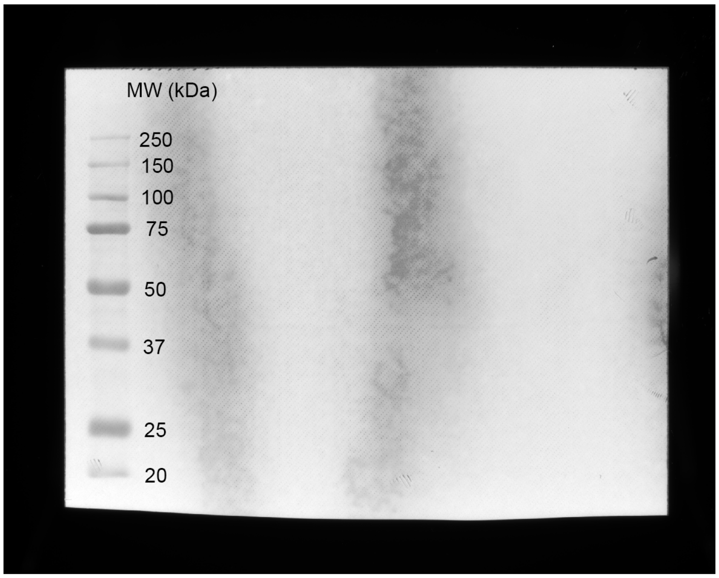

Figure 5C, α-FLAG panel: Chemiluminescence

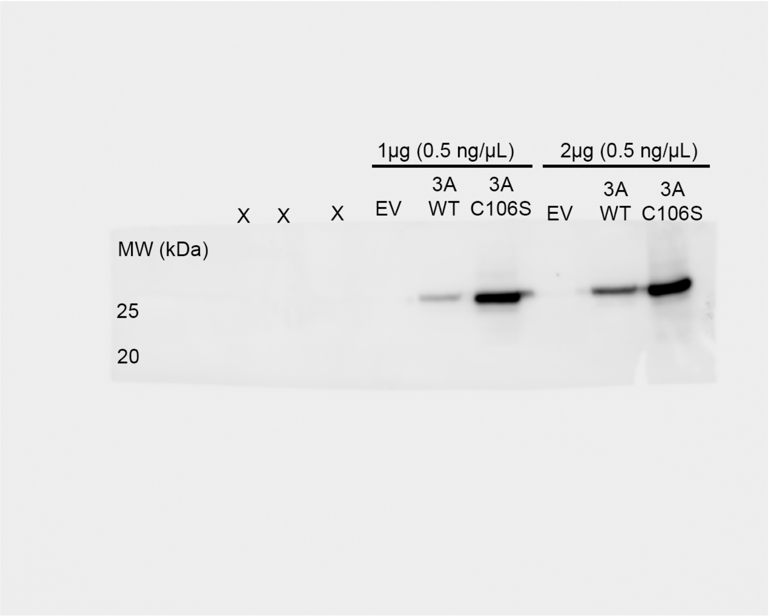

Figure 5C, α-FLAG panel: Colormetric

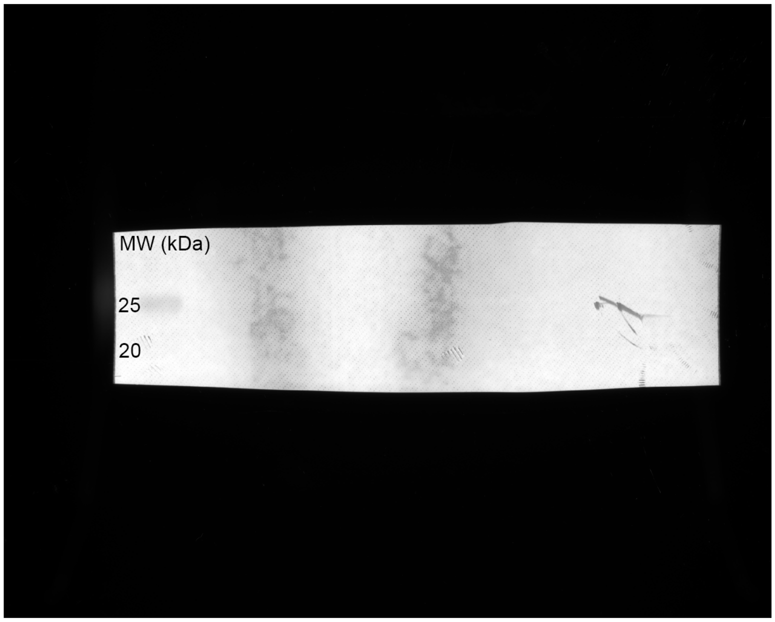

Figure 5C, α-β actin panel: Chemiluminescence

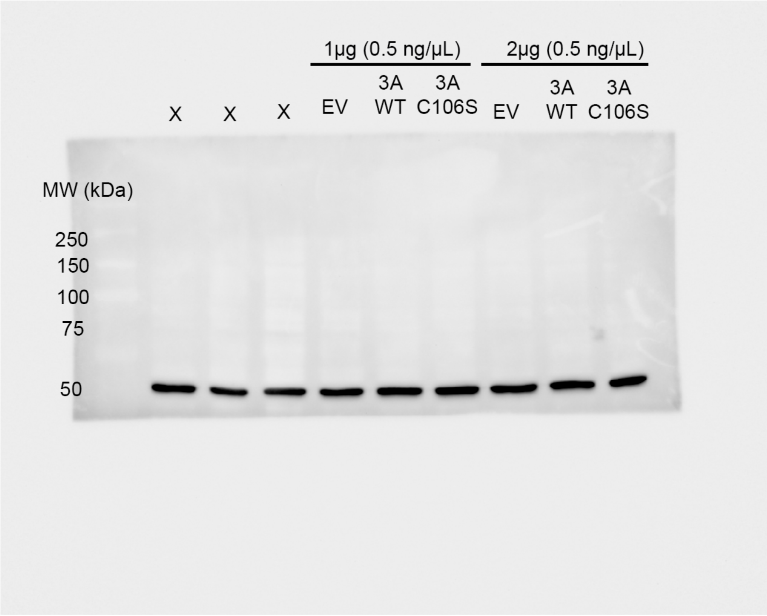

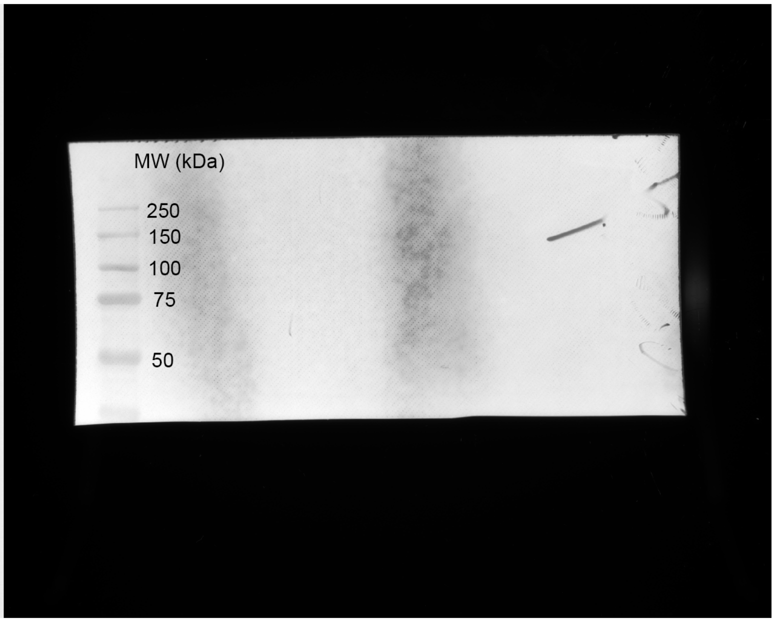

Figure S3A, α-APOBEC3A/B panel: Chemiluminescence

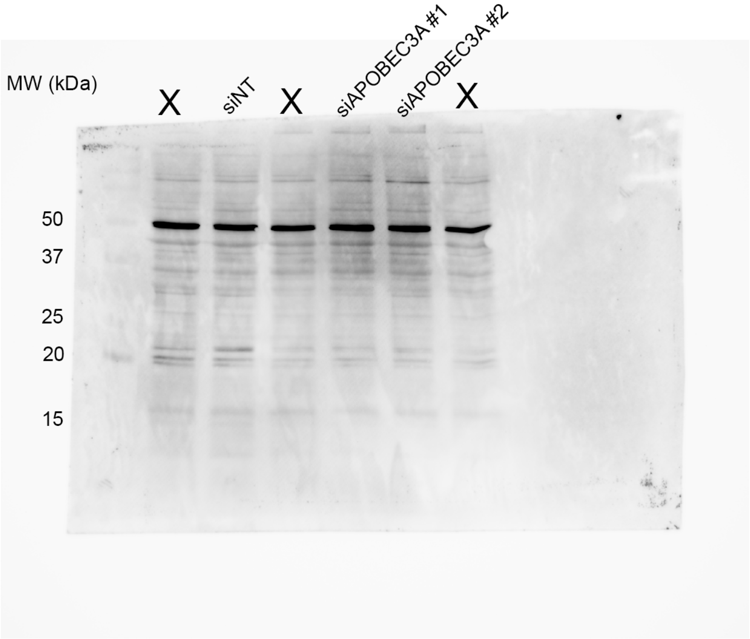

Figure S3A, α-APOBEC3A/B panel: Composite
Chemiluminescence and colormetric (for MW ladder visualization)

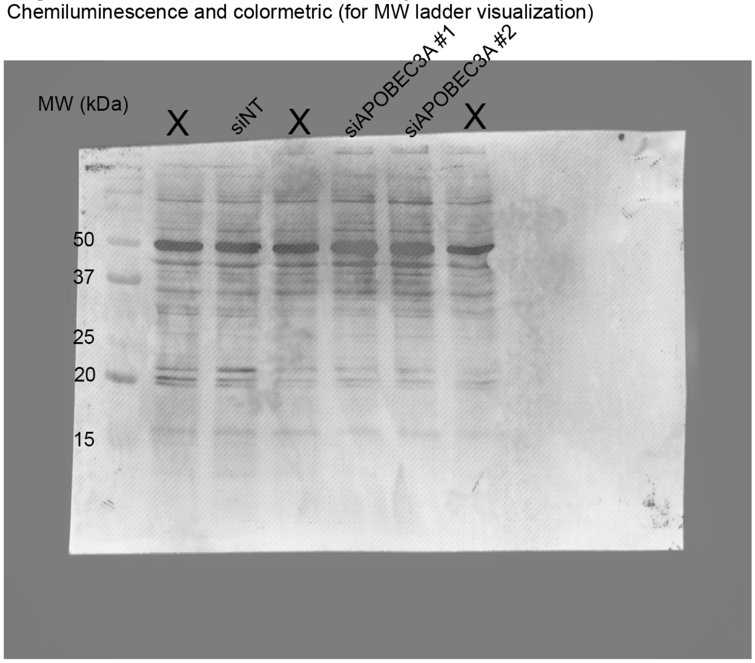

Figure S3A, α-β actin panel: Chemiluminescence

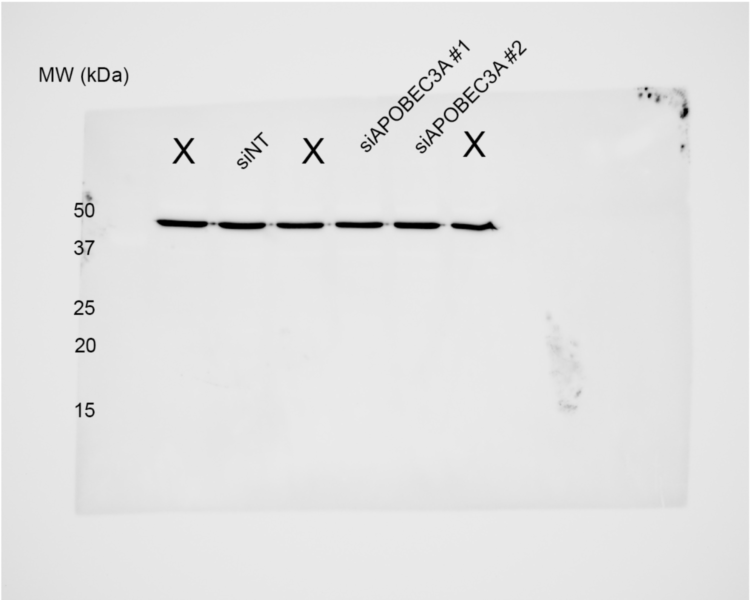

Composite α-APOBEC3A/B blot has MW markers more easily visibible for this blot (reprobed with α-β actin)

Note: This is also same blot image used for Figure 1E with different lanes analyzed here.

Figure S3B, α-puromycin panel: Chemiluminescence

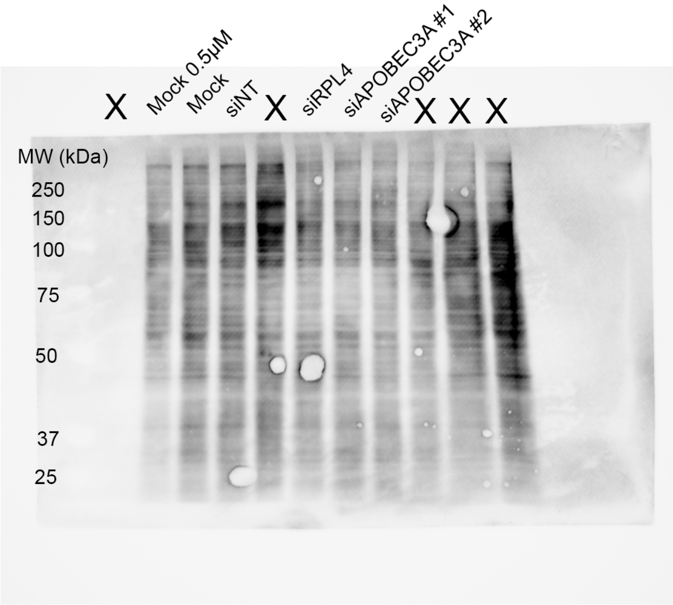

Figure S3B, α-puromycin panel: Composite
Chemiluminescence and colormetric (for MW ladder visualization)

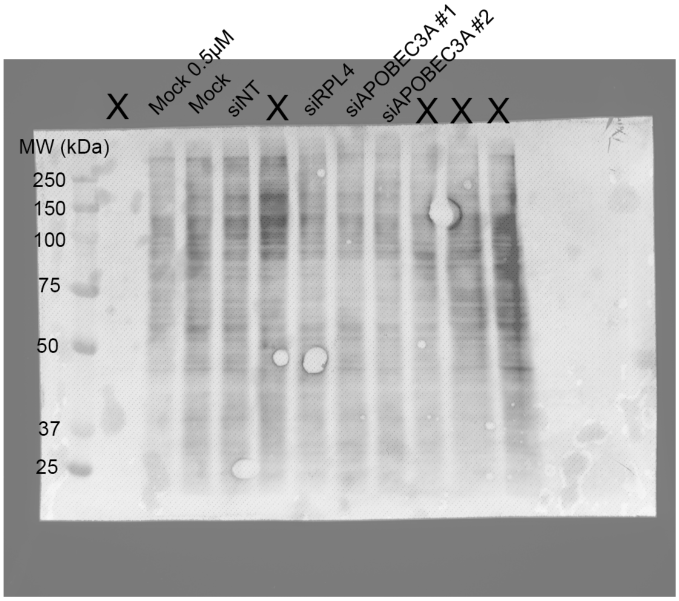

Figure S3B, α-β actin panel: Chemiluminescence

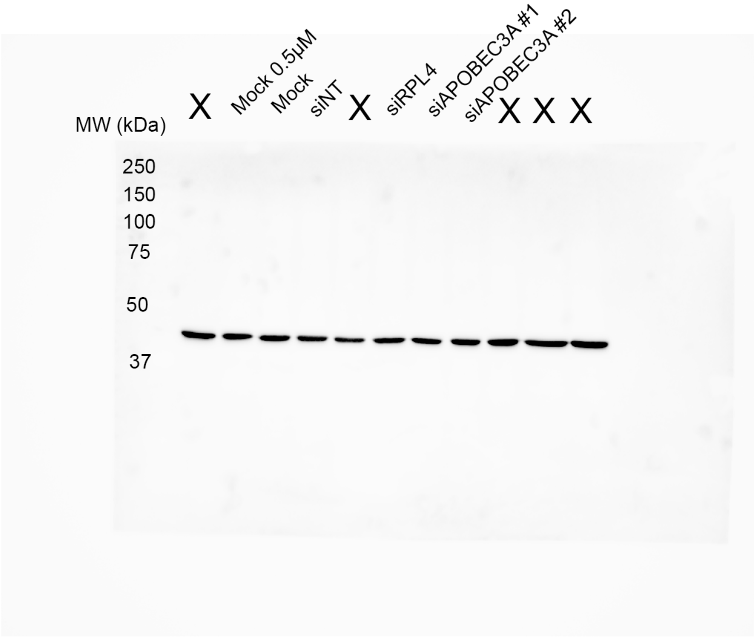

Figure S3B, α-β actin panel: Composite
Chemiluminescence and colormetric (for MW ladder visualization)

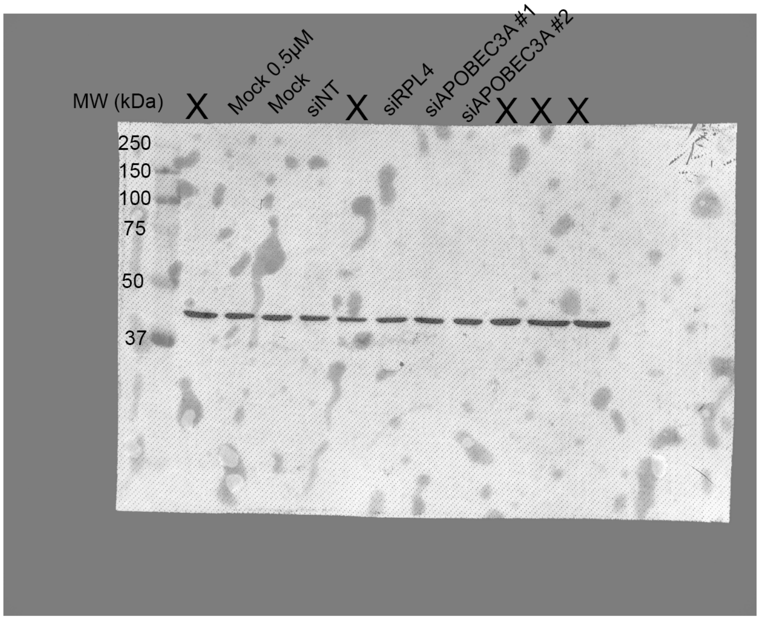

Figure S4A, α-APOBEC3A/B panel (3B): Chemiluminescence

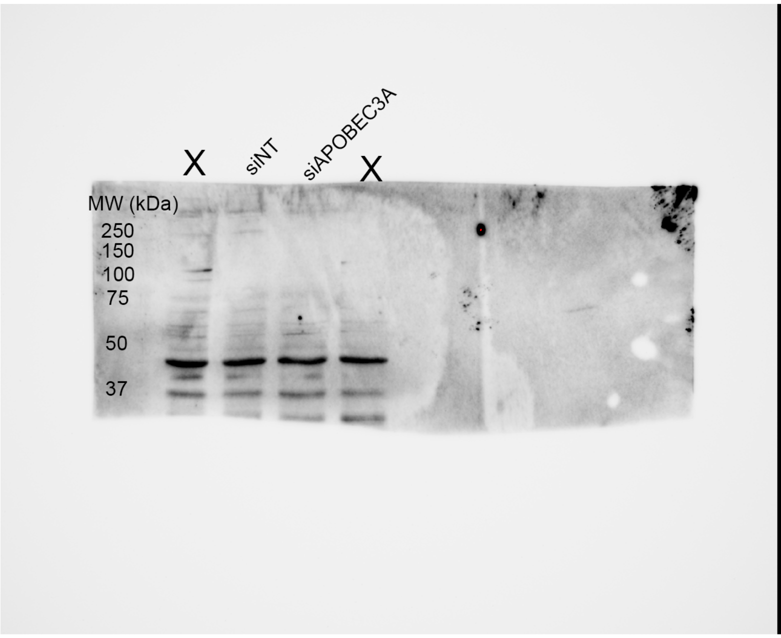

Figure S4A, α-APOBEC3A/B panel (3B): Colormetric
(for MW ladder visualization)

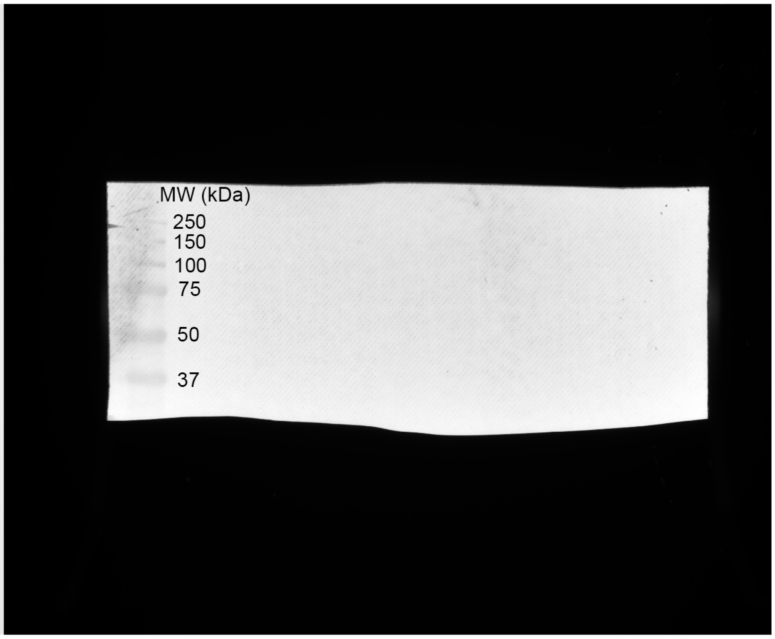

Figure S4A, α-APOBEC3A/B panel (3A): Chemiluminescence

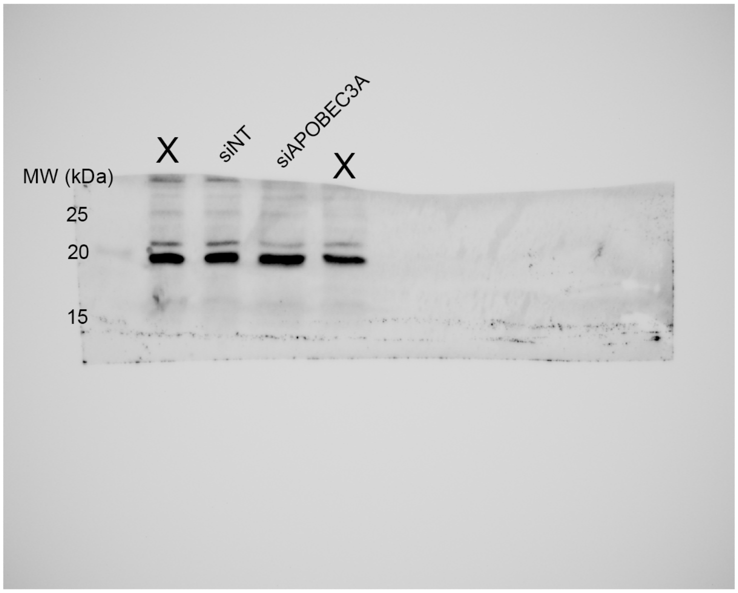

Figure S4A, α-APOBEC3A/B panel (3A): Colormetric
(for MW ladder visualization)

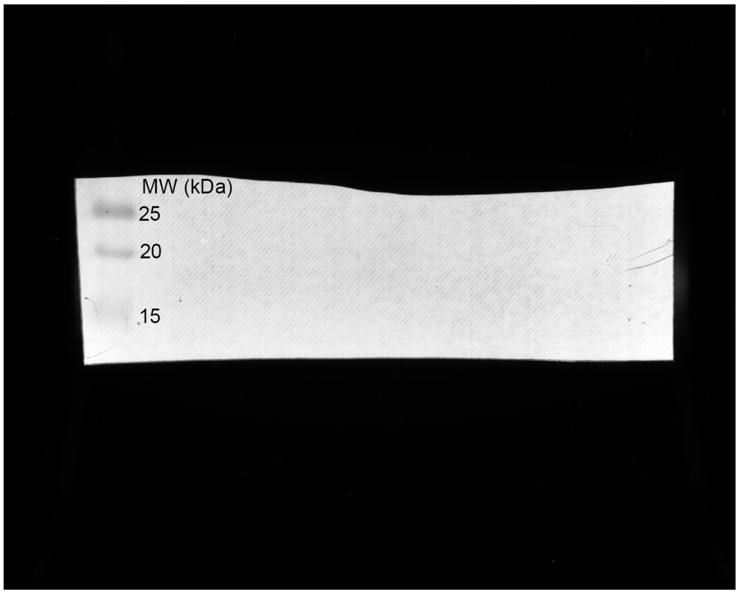

Figure S4A, α-β actin: Chemiluminescence

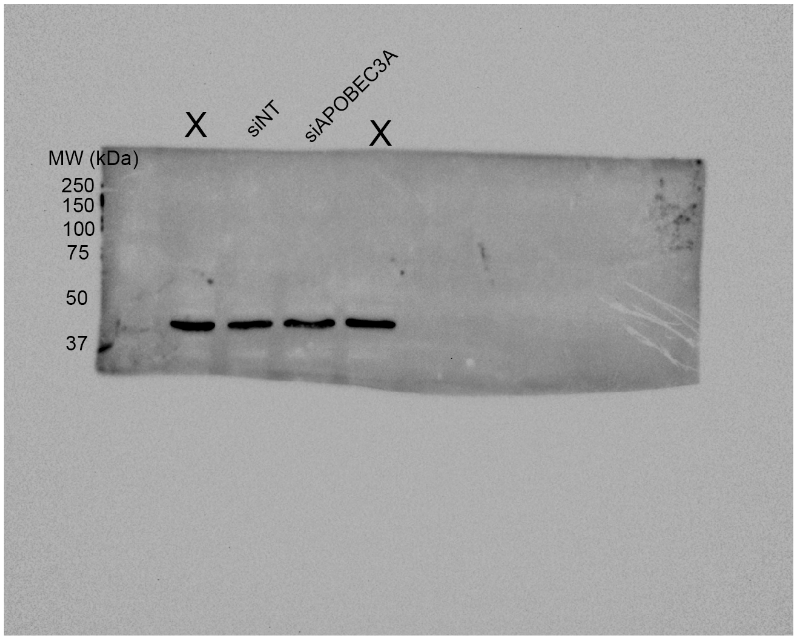

Colormetric α-APOBEC3A/B (3B) blot has MW markers more easily visibible for this blot (reprobed with α-β actin)

Figure S4B, α-puromycin panel: Chemiluminescence

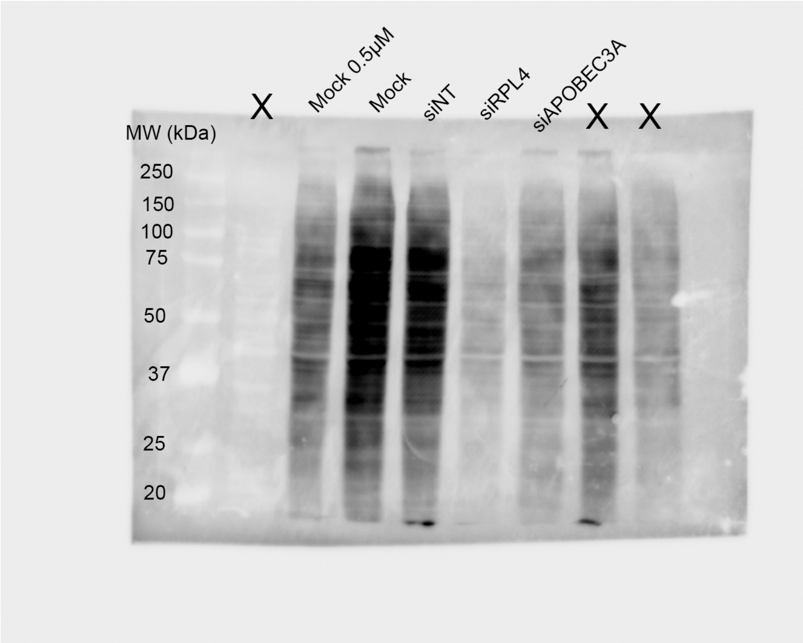

MW markers were easily visualized in chemiluminescence image, so did not acquire colometric image of this blot. Did acquire colometric image while reprobing blot for β actin below.

Figure S4B, α-β actin panel: Chemiluminescence

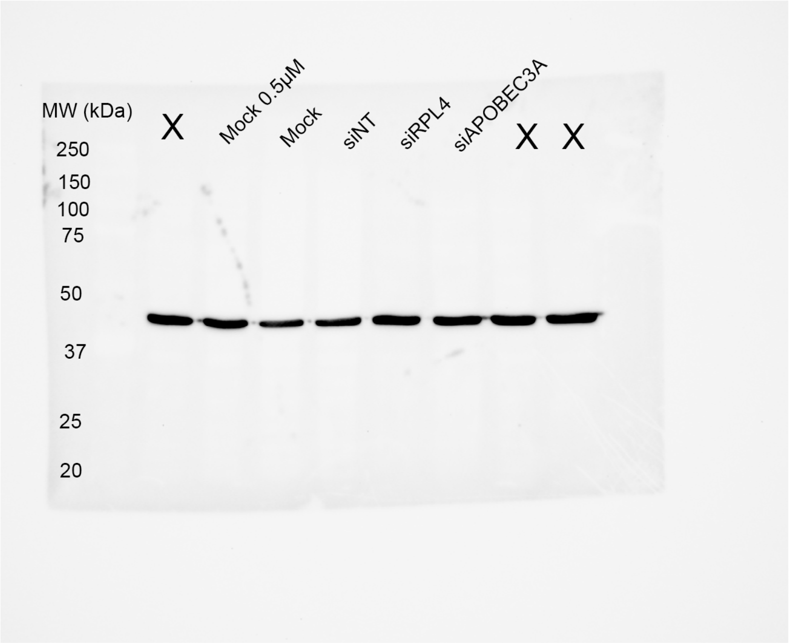

Figure S3B, α-β actin panel: Colormetric
(for MW ladder visualization)

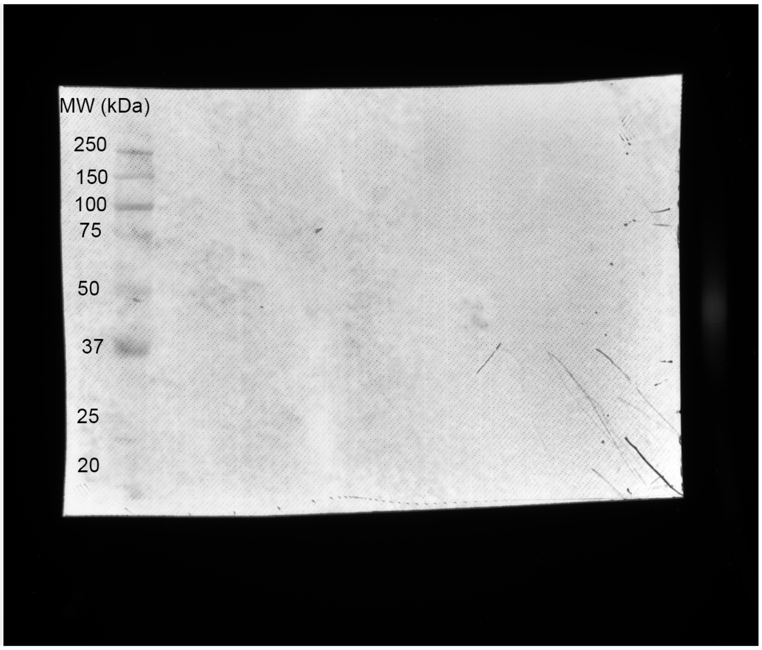

Figure S6, α-LAS1L panel: Chemiluminescence

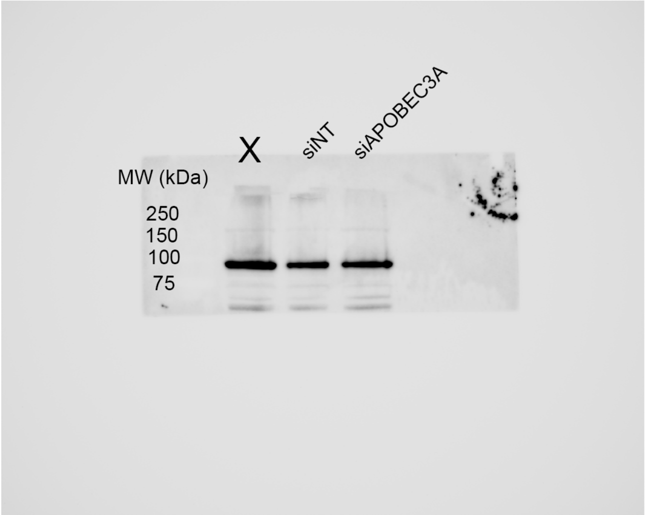

Figure S6, α-LAS1L panel: Colormetric
(for MW ladder visualization)

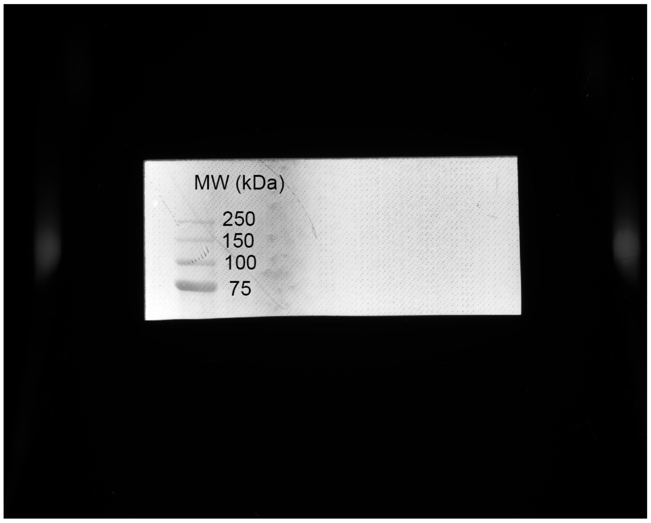

Figure S6, α-β actin panel: Chemiluminescence

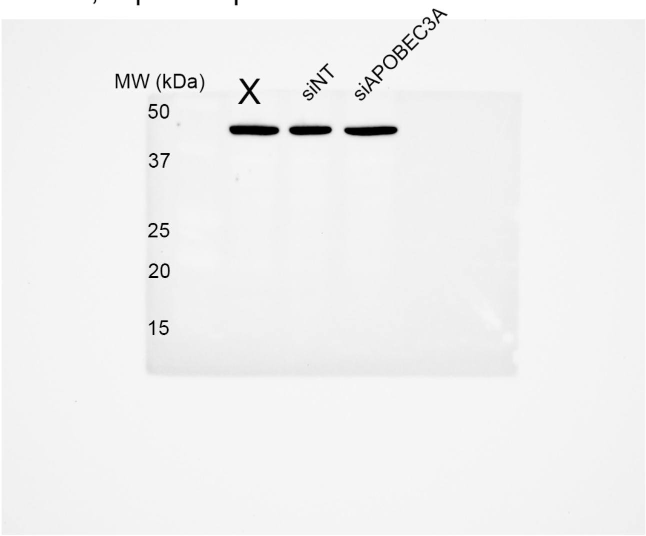

Figure S6, α-β actin panel: Colormetric
(for MW ladder visualization)

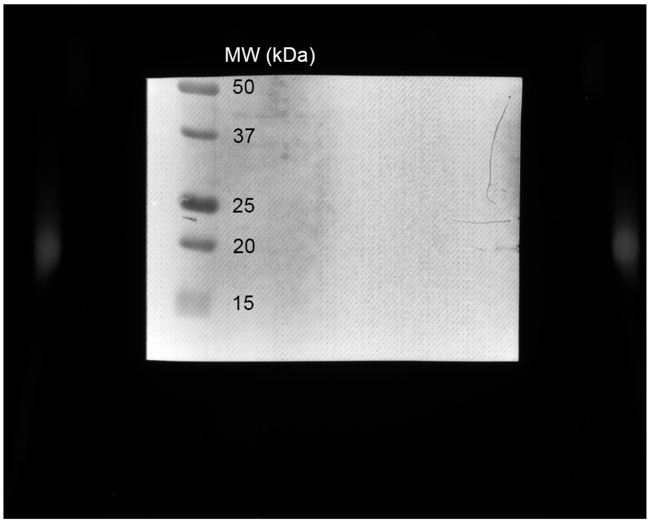

Figure S7A, α-FLAG panel: Chemiluminescence

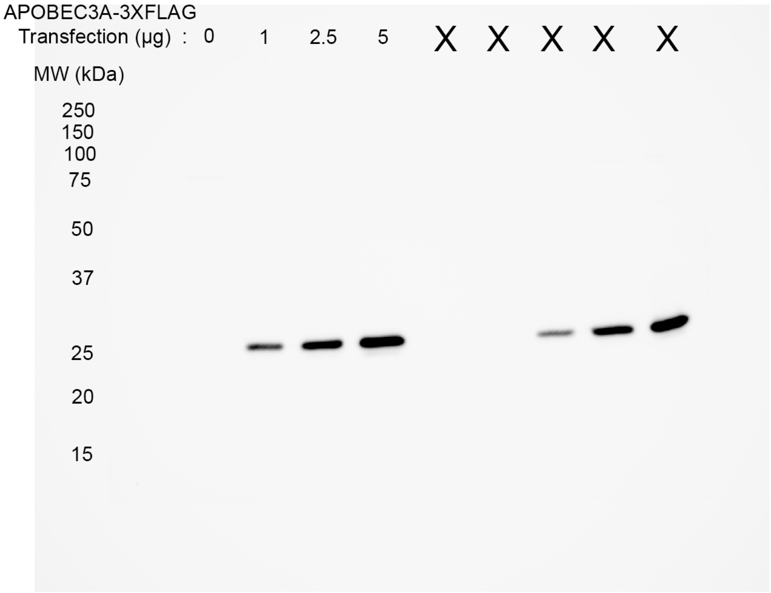

Figure S7A, α-FLAG panel: Colormetric
(for MW ladder visualization)

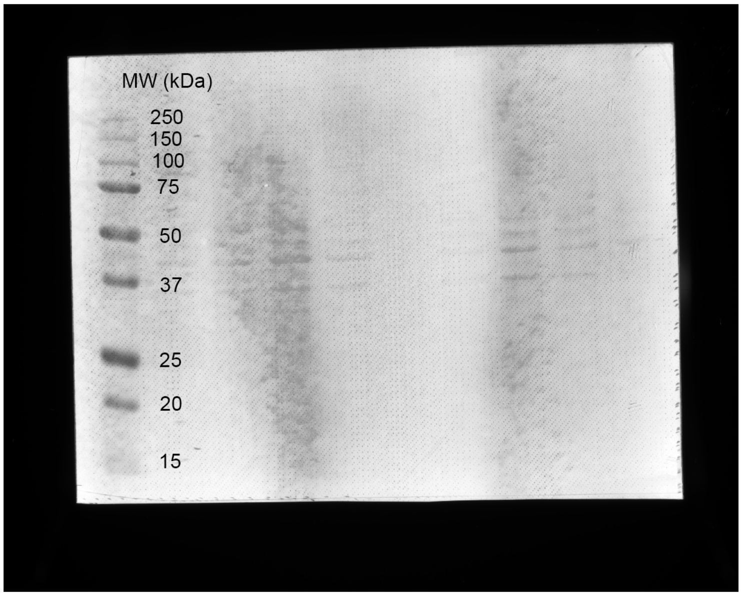

Figure S7A, B, total protein stain panel: Stain Free Blot
(Loading control and for MW ladder visualization)

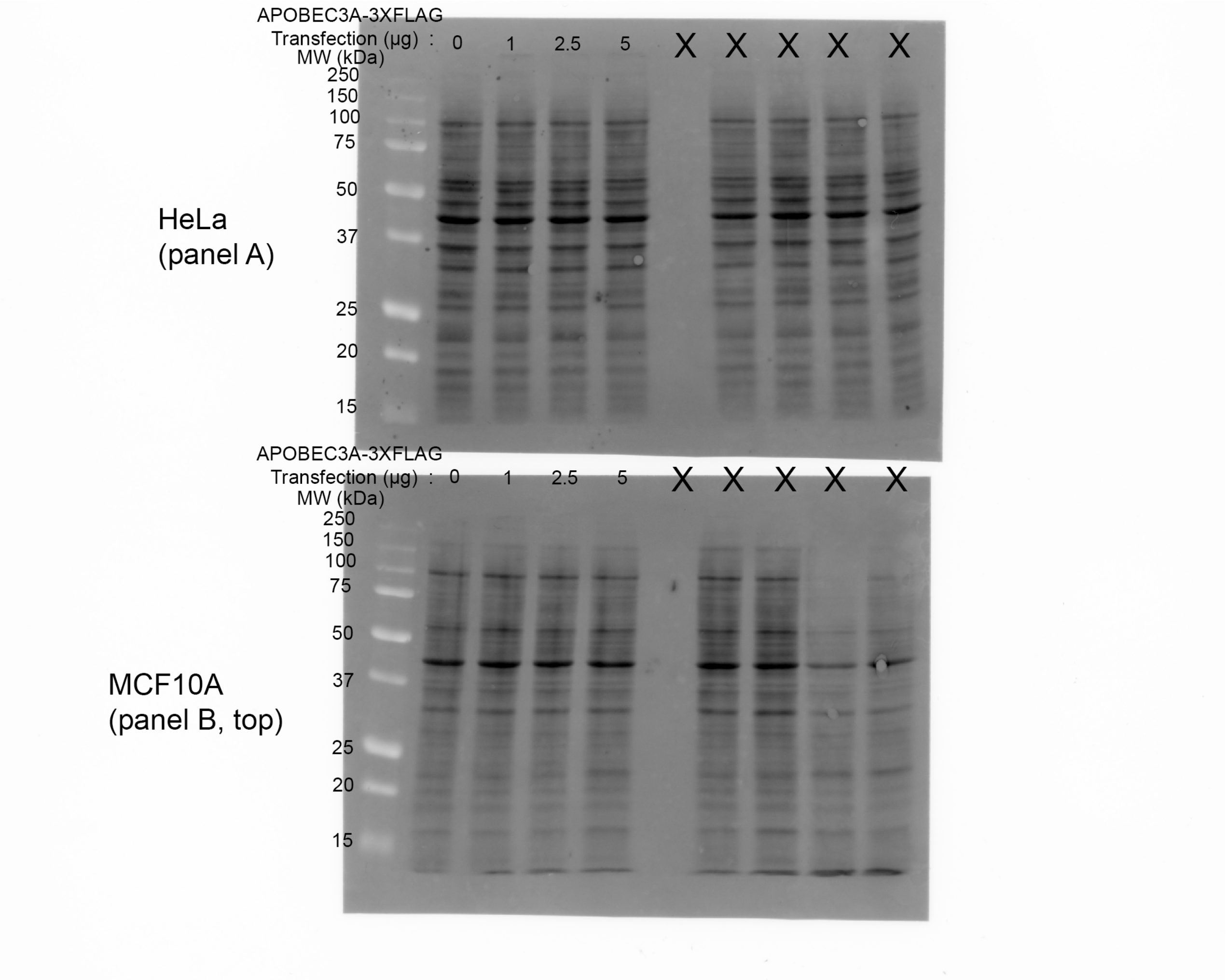

Figure S7B, α-FLAG panel (top): Chemiluminescence

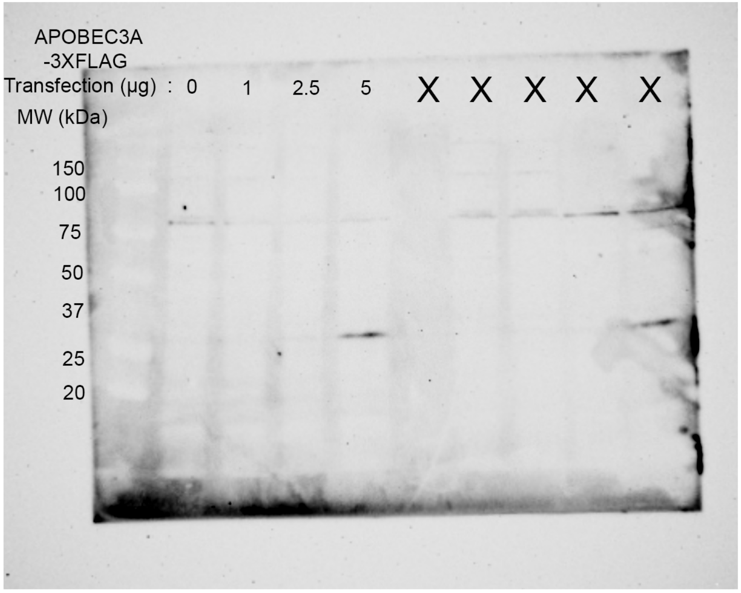

Figure S7B, α-FLAG panel (top): Chemiluminescence
(Darker exposure allowed for improved MW ladder visualization)

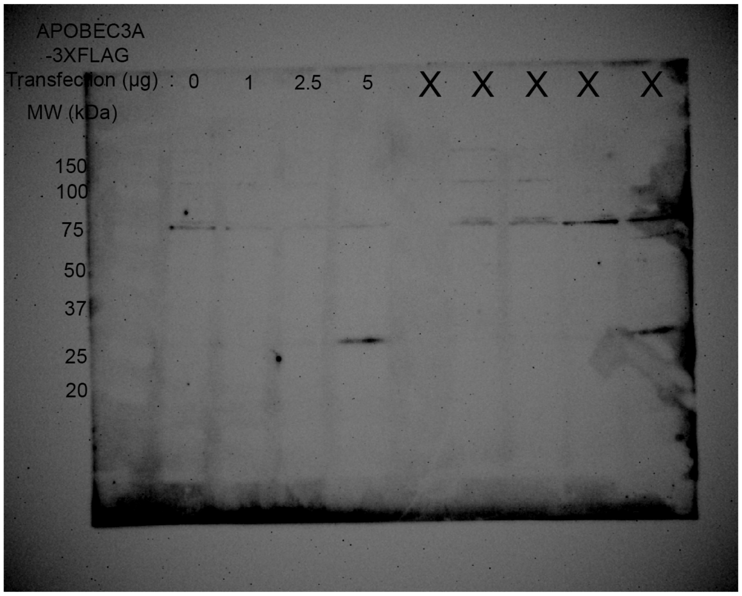

Figure S7B, α-FLAG panel (bottom): Chemiluminescence

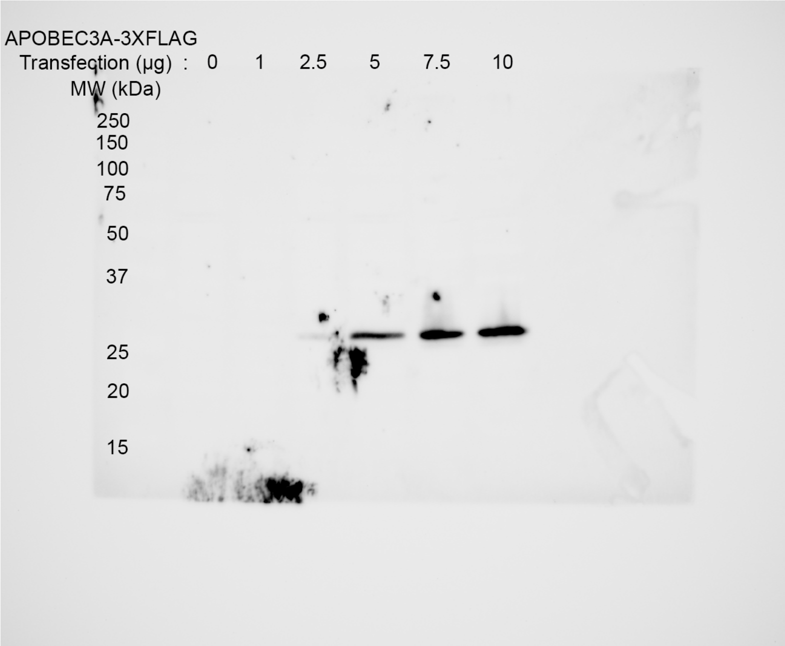

Figure S7B, α-FLAG panel (bottom): Colormetric
(for MW ladder visualization)

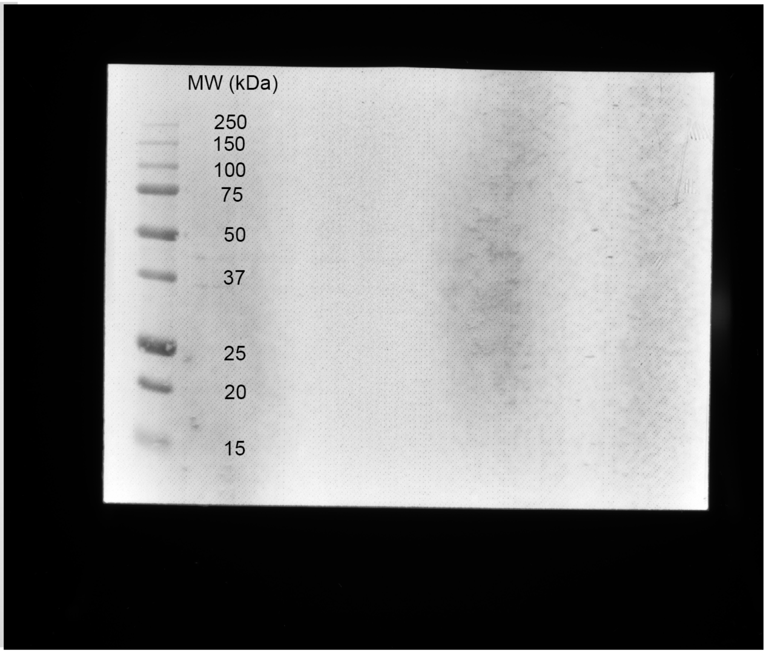

Figure S7B (bottom), total protein stain panel: Stain Free Blot
(Loading control and for MW ladder visualization)

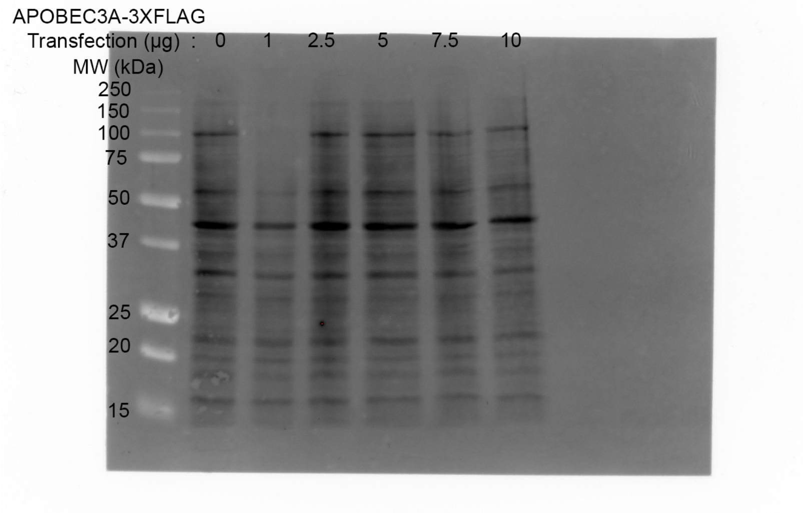

Figure S18, α-DDX17 panel: Chemiluminescence

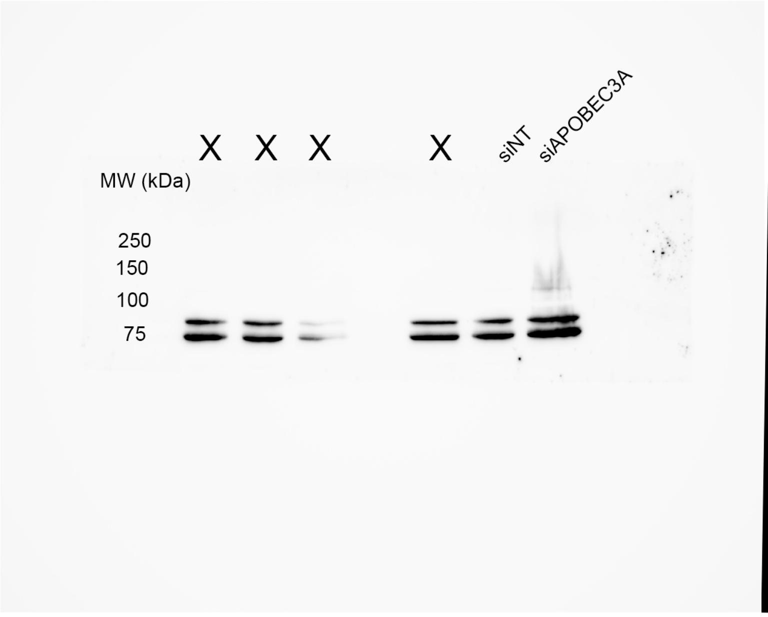

Figure S18, α-DDX17 panel: Colormetric
(for MW ladder visualization)

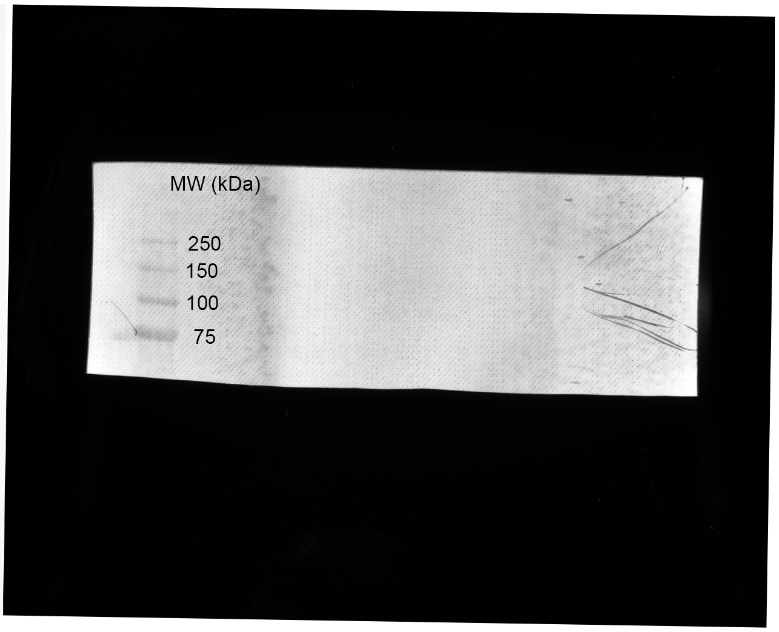

Figure S18, α-β actin panel: Chemiluminescence

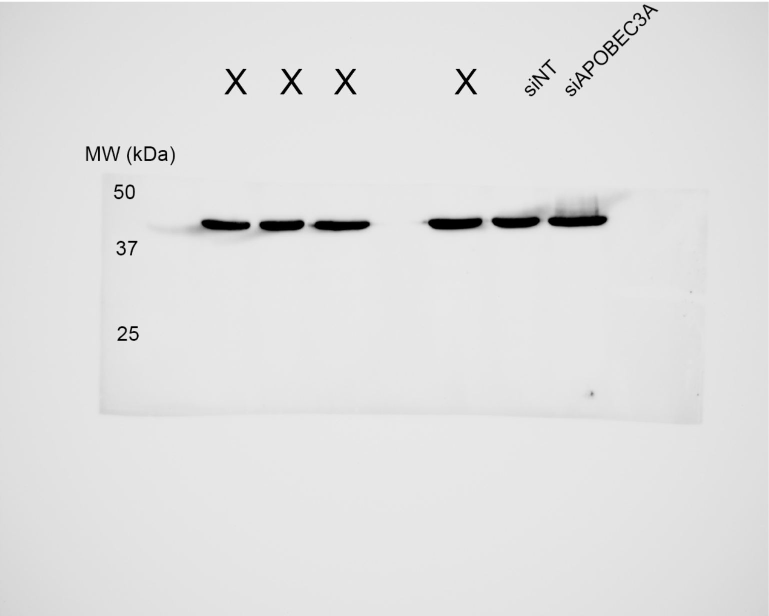

Figure S18, α-β actin panel: Colormetric
(for MW ladder visualization)

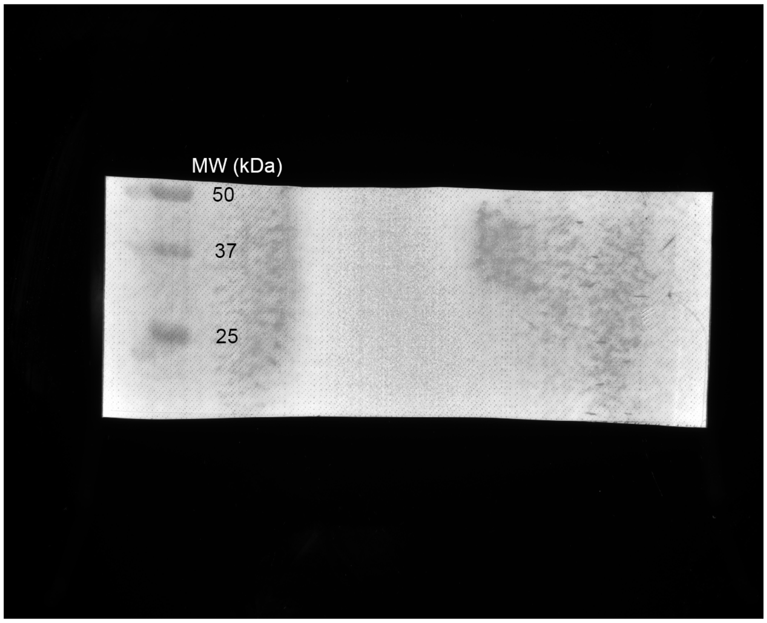

Supplement: S1 Raw Images — Original, uncropped, and minimally adjusted blot and gel images. (PDF) [file pbio.3002718.s021.pdf]
