# Supplementary material for: The cytidine deaminase APOBEC3A regulates nucleolar function to promote cell growth and ribosome biogenesis
Source: PLoS Biol. 2024 Jul 8;22(7):e3002718. doi: 10.1371/journal.pbio.3002718 (PMC11257408; doi:10.1371/journal.pbio.3002718)
Supplement: Attachment — Submitted filename: 12.30.23_PLOSBIOLOGY_ReviewerResponse_6.docx [file pbio.3002718.s022.docx]

We thank the reviewers for their time and consideration in offering constructive comments to aid us in improving our manuscript. In addition to responding to each reviewer’s specific comments, we have listed the major changes to our manuscript below. All updated/new text is indicated in red within a tracked changes version of the new manuscript document.

- **NEW** manuscript title based on new results and conclusions
- **MOVED** original Figure 3 to Supplemental. Now Figure S5.
- **NEW** Figure 4 with the APOBEC3A-FLAG immunofluorescence colocalization and co-immunoprecipitation results.
- **NEW** Figure 5 with the APOBEC3A wild-type and C106S catalytically dead mutated APOBEC3A overexpression cell growth and puromycin incorporation results.
- **DELETED** original Figure 7 (schematic based on our results) since did not adequately add towards explaining our findings.
- **NEW** Figure S6 (APOBEC3A depletion does not alter LAS1L protein levels)
- **NEW** Figure S7 (HeLa cells are more easily transfected with CMV-APOBEC3A-FLAG plasmid)
- **NEW** Figure S11 (Differential expression analysis of nuclear RNA-seq results comparing APOBEC3A depletion to non-targeting siNT negative control treatment)
- **NEW** Figure S12 (APOBEC3A depletion does not significantly alter predicted target DDX17 transcript or protein levels)
- **ADDED** 13 new references to better contextualized our results.
- **NEW** discussion section paragraph discussing the overexpression C106S catalytically dead mutated APOBEC3A results
- **UPDATED** interpretation of results to indicate it is likely APOBEC3A binding and not editing activity that is required for its role in making ribosomes. For example, changed “candidate APOBEC3A C-to-U edit sites” to “predicted APOBEC3A target sites” throughout the manuscript.
- **UPDATED** text throughout the manuscript (indicated in red)

REVIEWS:

Reviewer comments: Black text

Our response: Blue text

**Reviewer #1**: This manuscript follows up on previous work from the Baserga Lab identifying novel regulators of ribosome biogenesis (Farley-Barnes, Cell Rep 2018). In a previous high-throughput siRNA screen the authors identified APOBEC3A and APOBEC4 as hits based on the observation that their depletion leads to a one-nucleolus effect. Because APOBEC3A had stronger and more consistent results with the siRNA screen the authors chose to investigate its role in ribosome biogenesis further. The authors found that knockdown of APOBEC3A leads to changes in cell cycle progression, induction of the nucleolar stress response, and a decrease in global protein synthesis. Next the authors investigated if APOBEC3A plays a role in pre-rRNA transcription and ribosome assembly. Results from both a 5-EU incorporation assay and pre-rRNA transcription reporter assay were consistent with APOCEC3A playing a role in ribosome assembly downstream of POLI transcription. Quantification of RNA and northern blots from knockdown cells revealed that loss of APOBEC3A leads to 60S specific effects on pre-rRNA processing including a buildup of the 32S pre-rRNA and a decrease in the mature 28S rRNA.

The authors hypothesized that APOBEC3A could regulate ribosome assembly through either its C-to-U editing activity or its RNA binding activity. To explore this further the authors identified putative editing sites within the pre-rRNA and then performed RNA-seq experiments, which revealed putative editing sites within the pre-rRNA. The authors also identified putative editing sites in mRNAs encoding for nucleolar and cell cycle regulation genes. In the first four figures of the manuscript the authors present very convincing evidence that APOBEC3A is a regulator of the maturation of the large ribosomal subunit. However, they provide no concrete evidence for how APOBEC3A does this and if it is a direct or in-direct effect. More experimental evidence would be needed for the authors to propose that APOBEC3A edits pre-rRNA and/or nuclear pre-mRNA transcripts.

1. The authors could begin to tackle the direct vs indirect question through rescue experiments with catalytically inactive APOBEC3A.

Thank you for this important comment. We agree that completing more experiments towards deciphering if APOBEC3A plays a direct or indirect role in ribosome biogenesis, using a catalytically dead version of APOBEC3A, would provide necessary mechanistic insight. This conundrum is exemplified by two aspects of our original observations. Firstly, since we utilized a 72 h timepoint for APOBEC3A depletion, we observed defects in both the cell cycle and ribosome biogenesis, two tightly interconnected processes. Secondly, we observed predicted APOBEC3A target sites on both the pre-rRNA (direct) and pre-mRNAs encoding nucleolar proteins and cell cycle regulators (indirect). Since we focused on siRNA depletion experiments in our original submission, we decided to utilize an APOBEC3A overexpression approach to comparatively study APOBEC3A wild-type (3A WT) and a previously established catalytically dead mutated version (3A C106S) (Chen et al. 2006, Taura et al. 2019, Taura et al. 2022). These two constructs contain a C-terminal 3X-FLAG tag to assist with antibody recognition. Previous studies have demonstrated success analyzing APOBEC3A overexpression and tagging its C-terminal end (Sharma et al. 2015, Sharma et al. 2017, and Jalili et al. 2020). These experiments circumvent the issues tied to low levels of APOBEC3A expression and would be a more accurate model for APOBEC3A’s acute effects (24-48 hours), as opposed to long term genome mutagenic effects that occur in in cancer.

To answer if APOBEC3A is capable of functioning directly in ribosome biogenesis in the nucleolus, we expressed APOBEC3A WT in HeLa cells and performed immunofluorescence, co-staining with DAPI (nuclei) and FBL (fibrillarin; nucleoli). We observed moderate-strong colocalization of tagged APOBEC3A with the DAPI signal and moderate colocalization with FBL signal (**NEW FIGURE 4A, B**). Thus, a subset of tagged APOBEC3A localizes to the nucleolus where it can function directly in ribosome biogenesis. Next, using the same experimental setup, we performed co-immunoprecipitation experiments to test if tagged APOBEC3A associates with critical nucleolar proteins. By reciprocal co-immunoprecipitations, we show tagged APOBEC3A associates with RPA194 (the largest subunit of RNA polymerase I). Additionally, we detected tagged APOBEC3A association with FBL (fibrillarin; nucleolus) and KAP1 [a positive control that had previously been shown to associate with APOBEC3A (Taura et al. 2019) and RPA194 (Jang et al. 2018)], but not the pre-60S factor PES1 (**NEW FIGURE 4C, D, E**). These results indicate a subset of APOBEC3A is found within the nucleolus and interacts with RPA194 and FBL (see Reviewer 1, Comment 3 response for more information).

Next, we compared APOBEC3A WT versus catalytically dead mutated APOBEC3A C106S overexpression effects on cell growth and protein synthesis, the end function of competent ribosome production. Through a Cell Titer Glo 2.0 assay, we observed that both APOBEC3A WT and APOBEC3A C106S overexpression increased cell growth after 48 h which positively correlated with the amount of overexpression plasmid transfected (**NEW FIGURE 5B**). Similarly, APOBEC3A WT and APOBEC3A C106S mutant both increased protein synthesis as observed through a puromycin incorporation assay after 48 h only at higher amounts of overexpression (**NEW FIGURE 5C, D**). Since both WT and C106S mutant were able to increase cell proliferation and translation levels, it suggests APOBEC3A’s editing function is not required for its role in ribosome biogenesis. However, its presence in the nucleolus, where there are very high levels of pre-rRNA, indicates APOBEC3A likely binds the pre-rRNA as a direct function in ribosome biogenesis. Refer to Reviewer 3, Comment 7 response for more detailed information

Although our new experiments provided additional mechanistic insight into APOBEC3A’s direct role in the nucleolus, we still cannot rule out APOBEC3A also plays an indirect role through the binding/editing of pre-mRNAs that regulate ribosome production. Therefore, we have kept our discussion and our working model that our research indicates that APOBEC3A functions in making ribosomes in a direct manner, but it could also play an additive role indirectly too. This information provides valuable context for future APOBEC3A research that we are excited to follow-up on more in the future.

2. Given the strong association between APOBEC3A and cancer do the authors think that APOBEC3A's role in regulating ribosome assembly is specific to cancer cells (only MCF10A and HeLa cell lines were tested)?

Our data supports that APOBEC3A’s role in making ribosomes is not specific to only cancer cells. Firstly, the ATCC states that the MCF10A cell line we have used is from a woman with fibrocystic breast disease, which is not cancerous. The cell line, first described in Soule et al. (1990), has characteristics of normal breast epithelium and that it is non-tumorigenic in nude mice. We chose MCF10A cells due to their usefulness in screening for novel ribosome biogenesis regulators, which our lab has published on extensively in recent years (Farley-Barnes et al. 2018; Farley-Barnes et al. 2020; Ogawa et al. 2021; Bryant, McCool et al. 2022; McCool, Buhagiar et al. 2022; McCool et al. 2023).

Due to the strong connection between APOBEC3A and cancer, we included experiments using HeLa cells in our original manuscript submission. Furthermore, this connection has influenced us to use HeLa cells in our new additional experiments in our revised manuscript (**NEW FIGURES 4, 5, S7**). Our observations reveal that non-targeting siRNA treatment induces APOBEC3A expression (**Now FIGURE S13**) and that APOBEC3A overexpression increases translation and cell growth (**NEW FIGURE 5**). Our results perhaps suggest when APOBEC3A expression is increased (as in cancer), it can drive ribosome production and cell growth independent of its mutagenic capabilities; which has been the main focus on APOBEC3A in cancer research as exemplified by the articles published since our initial submission [Carpenter et al. 2023, McGuinness et al. 2023, Alder et al. 2023, Butt et al. 2023 (bioRxiv), and Sanchez et al. 2023 (bioRxiv), Isozaki et al. 2023, Naumann et al. 2023]

3. Apobec3A has established RNA binding activity. Is there any evidence to suggest it can associate with nascent pre-60S particles or other known regulators of ribosome assembly?

After extensively searching through the literature and available interactome databases [TheBioGrid (Oughtred et al. 2019) and HuMap2.0 (Drew et al. 2021)], we did not find any direct evidence that APOBEC3A associates with pre-60S particles or more generally with nucleolar proteins. However, we did find indirect evidence that suggests that APOBEC3A might interact with nucleolar proteins. Taura et al. (2019) detected association between APOBEC3A and KAP1 (also known as TRIM28). Interestingly, KAP1 is a promiscuous nuclear protein, but has several nucleolar proteins amongst its identified interactors, including the largest subunit of RNA polymerase I, RPA194 (Jang et al 2018). Therefore, we tested the extent to which APOBEC3A is present in the nucleolus and associates with pre-ribosome components. To answer this question, we expressed a C-terminally 3X-FLAG tagged APOBEC3A in HeLa cells and performed immunofluorescence staining and co-IPs with nucleolar proteins. Co-immunoprecipitations and immunofluorescence show that some of APOBEC3A is found in the nucleolus. Refer to Reviewer 1, Response 1 for results of these new experiments (**NEW FIGURES 4 ,5, S7**).

While we focused on testing for APOBEC3A association with critical regulators of ribosome biogenesis, such as RPA194 and fibrillarin, we also tested for one pre-60S specific interaction with PES1 which was negative (**NEW FIGURE 4E**). Additionally, we evaluated the extent to which APOBEC3A regulates pre-60S protein components, DDX17 and LAS1L, by looking for decreases in DDX17 or LAS1L protein levels after APOBEC3A depletion. However, we did not observe any decreases in either of their protein levels (**NEW FIGURES S6, S12**). This suggests APOBEC3A does not regulate DDX17 or LAS1L protein levels. A more comprehensive co-immunoprecipitation followed by mass-spec experiment would be required to identify a larger list of APOBEC3A interactors, which we hypothesize would include many other nucleolar proteins, including pre-60S factors.

4. The sequencing results presented in Figure 5 are confusing. I do not understand the graph presented in 5E. Are the significant editing sites those above the dashed line? Moreover, the edit frequencies observed are very low, making it difficult to understand how this could have a direct impact on ribosome assembly.

The significant edit sites are below the dashed line as we were looking for decreases in C to U editing after APOBEC3A depletion. The graph displays the editing frequency percent differences (for two independent biological replicates at various locations on the pre-rRNA) between siAPOBEC3A and siNT. If we are observing a APOBEC3A predicted target site then we would expect editing to decrease at this site when APOBEC3A is depleted compared to the non-targeting siRNA negative control.

We agree that our nuclear pre-rRNA sequencing C to U variant analysis after APOBEC3A depletion did not yield striking results both in terms of the editing frequencies and percent differences in frequency. However, these results align with our new results (**NEW FIGURE 5**) that the APOBEC3A C106S catalytically dead mutated version is also able to increase cell growth and global translation when it is overexpressed similarly to wild-type APOBEC3A. Thus, our results support the idea that APOBEC3A’s role in ribosome biogenesis is not dependent on its editing activity and is a potential reason why we only observed small changes in pre-rRNA C to U editing after APOBEC3A depletion. We have extensively updated the manuscript to reflect these findings that APOBEC3A’s catalytic activity is likely not required for its role making ribosomes.

5. Without direct evidence of C-to-U editing the authors should tone down some of their claims such as the following sentence (line 524) "Revealing for the first time the intriguing possibility that the pre-rRNA is edited …"

Thank you for pointing out the need to ensure our claims are fully supported by our results. Again, this and other suggestions led us to study a catalytically dead version of APOBEC3A in comparison to the wildtype. Since they yielded similar results in both increasing translation and cell growth after overexpression (**NEW FIGURE 5**), we have reworked our manuscript to reflect that APOBEC3A’s editing activity is likely not required for its role in making ribosomes. While our APOBEC3A C to U editing results are important to still include, we have taken out our claim that what we are observing is editing of the pre-rRNA.

Reviewer #2: Ribosome assembly is a complex pathway that is upregulated in cancer cells to support the increased demand for translation. However, ribosome assembly is energy intensive and the pathway is normally strictly regulated according to cellular needs and nutrient availability. A deep understanding of the ribosome assembly pathway in humans may identify potential targets for drug therapies against cancers. Previously, the Baserga lab screened for factors that altered nucleolar morphology to identify novel ribosome assembly factors. Among their previous hits were APOBEC3A and APOBEC4, cytidine deaminases previously not known to be involved in ribosome assembly. Here, the authors more closely examine the potential roles of these deaminases in ribosome assembly. The experimental data are clearly presented and the experiments appear to be well-executed. The authors provide preliminary results suggesting APOBEC3A impacts assembly of the large subunit. However, their characterization of how APOBEC3A affects ribosome assembly, whether through editing the rRNA, partaking directly in the assembly pathway or editing the mRNA of a ribosomal protein gene or assembly factor or editing another RNA species such as snoRNAs, is lacking. Overall, the analysis is quite preliminary and does not provide new mechanistic insights into ribosome assembly. Considerable additional experimental evidence is needed to substantiate the authors' primary claim, that APOBEC3A plays a crucial role in 28S assembly and to provide insight into its mechanism of action in ribosome assembly. Additionally, the writing needs careful editing for logical connection of ideas.

Major points:
1) The authors characterize the phenotype of cells after knockdown of APOBEC3A. The authors should show that an si-resistant APOBEC3A can rescue the knockdown. In addition, the authors should determine if a catalytic mutant of APOBEC3A can rescue to determine if it is the editing function of APOBEC3A that is required, or if the protein plays a chaperoning role as seen for many RNA modifying enzymes in ribosome assembly.

Thank you for suggesting these key experiments that other reviewers were also interested in. We have taken all these similar reviewer comments together and addressed them in our revised manuscript with inclusion of several new experiments.

Refer to Reviewer 1, Comment 1 and 3 Response

Additionally, we opted for overexpression experiments in HeLa cells to test if increased levels of APOBEC3A wildtype or catalytically dead mutant could promote increases in cell growth and ribosome biogenesis. By performing overexpression experiments, we were able to go beyond testing if reintroducing APOBEC3A could rescue ribosome biogenesis to only normal levels. We were able to observe if acute high levels of APOBEC3A could increase ribosome function and cell proliferation above normal levels. In doing this, we improved our manuscript’s relevance to APOBEC3A’s role in ribosome production in connection to cancer.

Some previous work studying APOBEC3A’s function in cell culture either used solely knockout or siRNA/shRNA depletion (Cortez et al. 2019, Alqassim et al. 2021, Petljak et al. 2022) or solely overexpression (Sharma et al. 2017). However, other previous work performed both knockdown/knockout and overexpression experiments as we did here (Sharma et al. 2015, Niavarani et al. 2015, Taura et al. 2019, Taura et al. 2022). We believe that our inclusion of both types of experiments is a new strength of our study.

2) The two primary pieces of evidence that APOBEC3A has a role in ribosome assembly are: a) knockdown of APOBEC3A leads to elevated levels of 32S pre-rRNA intermediate and b) to reduced levels of 28S. This is a good starting point for additional analysis. For example, the authors should determine if APOBEC3A interacts with ribosome assembly factors. This could be done by co-IP or Bio-ID and mass spec. If such factors or interactions can be identified, then the authors should ask if these interactions are important for ribosome assembly.

Thank you for this suggestion to further our understanding of APOBEC3A in ribosome production.

Refer to Reviewer 1, Comment 1 and 3 Response

We are excited to report for the first time that not only APOBEC3A functions in ribosome biogenesis but a tagged APOBEC3A is present in the nucleolus and associates with nucleolar proteins RPA194 and FBL. Unfortunately, to directly test if these interactions are required for ribosome assembly would require a massive amount of work beyond the scope of this manuscript. First, we would have to identify interacting residues using cross-linking mass spec in-cellulo or in-vitro experiments. Then, we would have to mutate these residues and ensure they do not alter normal protein function/folding before testing them in our functional ribosome biogenesis assays. However, we do hope to answer these types of questions in the future to define APOBEC3A’s role more precisely in the nucleolus.

3) Fig 2. In panel C, the authors show that knockdown of APOBEC3A leads to reduced puromycin incorporation, a surrogate for translation. However, a similar result would result from any of numerous cell stress conditions leading to accumulation of monosomes. The authors should show polysome profiles of these cells to establish that these cells contain polysomes.

For our measurements of protein synthesis using puromycin incorporation, we are careful to include both positive and negative controls to ensure the most interpretable results. We control for siRNA transfection using an additional negative mock control, which contains no siRNA yet produced similar results to siNT treatment. Furthermore, our Mock 0.5μM control shows the sensitivity and quantitative nature of our results. We also included a positive control, siRPL4, which we have established as a good positive control for depletion of factors involved in large subunit ribosome biogenesis (Bryant et al. 2023).

Secondly, we performed several assays that are upstream of the puromycin incorporation assay to show that a subset of APOBEC3A resides in the nucleolus (**NEW FIGURE 4A, B**), APOBEC3A associates with crucial ribosome biogenesis factors (**NEW FIGURE 4C, D, E**), it is required for pre-60S processing of the 32S pre-rRNA intermediate, and ultimately leads to reduced mature 28S rRNA levels (**Now Figure 3**). These results point towards APOBEC3A’s role upstream in ribosome biogenesis that leads to the decrease in global translation observed upon its depletion. We cannot rule out that APOBEC3A has other more direct effects on translation itself, which is beyond the scope of our manuscript. However, since we observed a similar reduction in puromycin signal with the positive control, siRPL4, which is also required for 32S pre-rRNA processing (Jongmans et al 2018), we think it is the lack of mature ribosomes being formed that is the main cause for a reduction in translation.

Lastly, we have now included a puromycin incorporation assay upon overexpression of APOBEC3A (**NEW FIGURE 5C, D**). If we were observing general cellular stresses by use of siRNAs, then we would not expect overexpression of APOBEC3A to have the inverse effect as its depletion. These new results further substantiate APOBEC3A’s role in making ribosomes and thus global translation.

4) Fig 3 suggests that APOBEC3A is not involved in transcription. This figure is more appropriate for supplemental material.

Thank you for this suggestion. To better emphasize our exciting positive results, we have moved this figure, which is mostly negative data, to the supplemental figures (**Now Figure S5**).

5) Fig 5 deals with the identification of edits in rRNA. The authors conclude in panel A that the spacer regions are enriched for edit sites. However, another interpretation appears to be that 18S and 28S rRNAs experience less editing than surrounding sequence. This should be addressed.

Figure 5A shows all the potential APOBEC3A target sites on the pre-rRNA based on the presence of the UC sequence motif over what is expected at random. Here we only analyzed the human pre-rRNA consensus sequence. It is not a presentation of any results we obtained when testing APOBEC3A as in the other figure panels, but a bioinformatic analysis. As the reviewer correctly points out, we sought to identify if there are any portions of the pre-rRNA that we would expect APOBEC3A to act on based on the presence of APOBEC3A UC sequence motifs. The only APOBEC3A predicted target sites that we identified are shown in panel 5D and labeled on the pre-rRNA schematic above the graph in panel 5A with green or grey asterisks.

We have updated our results text to more accurately describe our findings that the UC dinucleotide sequence motif is enriched in the internal transcribed spacer sequences (ITSs) and unenriched within the 18S and 28S rRNA sequences.

We now state the following: “Based on examining UC sequence motif occurrence at 100 nucleotide intervals across the transcript and its enrichment over its expected occurrence at random (~6.25 per 100 nucleotides), we observed that this motif was more likely to occur within the ITS sequences and less likely to occur within mature rRNA sequences on the 47S pre-rRNA compared to what is expected for a random sequence (Fig 6A). More specifically, there were sites of UC motif enrichment just 5’ of cut sites 1 in the 5’ETS, cut site 2a in ITS1, and cut site 4 in ITS2, and an overall decrease below what would be predicted across the 18S rRNA and at some locations on the 28S rRNA.”

From our analysis, we interpret these results to mean that the spacer sequences have an enrichment of UC sequence motifs. It led us to hypothesize that APOBEC3A would have target sequences within these regions, and we ultimately did identify a couple of predicted target sites within these regions (**Now Figure 6D, E**).

6) Although the authors have identified apparent edits in rRNA, their frequency is exceptionally low. For those in 28S that appear to be APOBEC3A-dependent, at most, only 1.1% of the RNA is edited. This cannot account for the substantial loss of 28S. The accumulation of 32S implies a defect in cleavage within ITS2 and the only APOBEC3A-dependent site in ITS2 is edited at less than 0.5%, again, not explaining the loss of 28S. Furthermore, the change in editing frequency upon APOBEC3A knockdown is very modest, less than 10%. Collectively, these results do not establish that rRNA editing by APOBEC3A impacts ribosome assembly. Rather, they suggest that rRNA editing is not important for assembly.

We agree this level of editing is likely not required for the universal process of ribosome biogenesis in all human cell types and contexts. These results and helpful reviewer suggestions motivated us to ask if APOBEC3A’s catalytic activity is required for its role in ribosome production. Therefore, we performed APOBEC3A overexpression experiments in HeLa cells with wild-type and a C106S catalytically dead mutated version (**NEW FIGURE 5**). Aligning with our sequencing results that APOBEC3A’s role in ribosome biogenesis is independent of its editing function, both overexpressing APOBEC3A wild-type and C106S increased global translation and cell growth compared to an empty vector negative control (**NEW FIGURE 5**). We have adjusted the manuscript significantly to highlight that our results point towards APOBEC3A’s catalytic activity not being required for its role in making ribosomes and that it is more likely a RNA binding/chaperoning function.

7) In Figure 6, the authors turn to the possibility that APOBEC3A editing of pre-mRNA or mRNA can explain its role in ribosome assembly. Here, the authors identify a multitude of candidate genes and call out DDX17 as an intriguing candidate because it has a known role in ribosome assembly. However, the authors do not do any experiments to test the function of APOBEC3A editing this transcript or any others in their candidate list. Again, this is a good starting point for additional directed analysis which needs to be done.

*DDX17* was the most intriguing (pre)-mRNA that contained a predicted APOBEC3A target site within its 3’UTR. We were excited to follow up more on APOBEC3A’s potential to regulate *DDX17* and thus its function in pre-60S biogenesis. To this end, we performed qRT-PCR to measure DDX17 mRNA levels and western blotting to measure its protein levels of both isoforms (p72 and p82) after APOBEC3A siRNA depletion. We hypothesized that APOBEC3A editing/binding of *DDX17* could influence its stability on its mRNA, steady-state protein levels present, and/or alter the relative levels between its two alternative translation start site isoforms (Jalal et al. 2007).

We observed a trending, but not significant decrease (p=0.09) in *DDX17* mRNA levels by qRT-PCR in MCF10A cells after APOBEC3A siRNA depletion (**NEW FIGURE S12 B**). This indicates APOBEC3A might play a role in regulating *DDX17* mRNA steady-state levels, but likely not to a large enough extent to reduce pre-large ribosomal subunit processing to the levels we observed in our experiments. Further emphasizing this point, APOBEC3A depletion only slightly increased or did not change DDX17 (p82 and p72) protein levels and did not alter the ratio between the two translation start site isoforms (**NEW FIGURE S12 C**). While we appreciate the reviewers shared interest in learning more about APOBEC3A’s specific regulation on *DDX17* and other (pre)-mRNAs that it has predicted target sites within, the amount of work required to understand APOBEC3A’s regulation of all or some of those transcripts is beyond the scope of the work we present here.

8) In various places, the logic presented seems confused. One example: L35-38: The authors try to connect the mutagenic role of deaminases in cancer with their potential role in ribosome assembly. This writing confuses the issues. Mutagenesis by deaminases is not driving ribosome assembly and ribosome assembly is needed for but does not drive cancer. The role that the authors propose for deaminases in ribosome assembly is unrelated to and conceptually distinct from their role in cancer.

We agree that there are two distinct roles for APOBEC3A in relation to cancer. Firstly, as we discuss in our introduction, APOBEC3A has a well-established mutagenic role in oncogenesis. At first we were unsure if this role would be distinct from its role in ribosome biogenesis, however our new data studying overexpression of the catalytically dead C106S mutated version (**NEW FIGURE 5**) suggests APOBEC3A’s editing role and thus mutagenic role is not required for its role in ribosome biogenesis. Furthermore, APOBEC3A’s presence in the nucleolus (**NEW FIGURE 4A, B**), and association with nucleolar proteins (**NEW FIGURE 4C, D, E**) suggests APOBEC3A is directly involved in ribosome production within the nucleolus. We have significantly overhauled the manuscript to better describe our results and emphasize this point.

Refer to Reviewer 1, Comment 2 Response

Minor points
1) The logogram in Fig 6E should show RNA sequence (eg, U not T).

Thank you for pointing out this potential point of confusion. We have updated this figure to represent the RNA sequence since that is what was directly tested. However, we would like to point out that our work does not disentangle APOBEC3A’s DNA vs RNA editing function since these detected edits could have occurred at the DNA or RNA level.

2) L243: This is not correctly stated: "Since the nucleolar stress response is caused by a reduction in mature ribosome levels…" Nucleolar stress leads to reduced levels of mature ribosomes but I am not aware of evidence that reducing the levels of mature ribosomes leads to nucleolar stress. (And this is not the conclusion of the citations.)

We politely disagree. We argue that the recent paper by Hannan et al. (Cell Reports 2022) provides strong evidence that depletion of many nucleolar ribosome biogenesis factors that are essential for making ribosomes causes the nucleolar stress response (TP53 stabilization). We have added this reference here. Part of the confusion may be coming from the fact that scientists use the term “nucleolar stress response” to refer to different things. We are using it here to refer to TP53 stabilization, and have defined it that way. Thank you for asking for the clarification.

3) L530: It is an overstatement to write: "we defined a role for APOBEC3A in ribosome biogenesis in the nucleus…"

We agree that this was an overstatement in our original submission. We hope that our new experiments looking at APOBEC3A’s localization by immunofluorescence (**NEW FIGURE 4A, B**) and identifying physical association with key nucleolar proteins (**NEW FIGURE 4C, D, E**) points towards APOBEC3A functioning in the nucleus and more specifically the nucleolus regarding ribosome production. We have reworked the text to reflect our both initial and new findings with this in mind.

In an effort to be clearer and more concise we have deleted the phrase “in the nucleus” here.

4) L532: The authors claim: "We discovered that the human cytidine deaminases, APOBEC3A and APOBEC4, are candidate ribosome biogenesis factors through validation of our previous genome-wide siRNA screen." This holds for APOBEC3A but not APOBEC4.

While our results presented in Figure 1 were validations of our lab’s previous genome-wide siRNA screen (Farley-Barnes et al. 2018), we understand that much more validation was completed for APOBEC3A throughout the rest of the manuscript. We have updated multiple parts of the text to properly reflect our findings and focus on APOBEC3A.

We have changed the text referenced in this comment to now state: “We discovered that the human cytidine deaminase, APOBEC3A is a strong candidate ribosome biogenesis factors through validation of our previous genome-wide siRNA screen”

Additionally, we previously stated in the introduction: “We validate APOBEC3A and APOBEC4 as two novel ribosome biogenesis factors.” We now state in the introduction: “We validate the previous screening results for APOBEC3A and APOBEC4, but ultimately focus on APOBEC3A due to its more compelling validation results.”

5) L106: "decrease in cancer survival" should read "…patient survival"

Thank you for pointing out this error. We have updated the text to read “patient survival” now.

Reviewer #3: In their manuscript entitled "The cytidine deaminase APOBEC3A is required for large ribosomal subunit biogenesis", the authors report the interesting discovery that RNA/DNA deamination and ribosome assembly are linked processes. This finding may be of relevance as both APOBEC3A and ribosome biogenesis are implicated in cancer development. Based on a previous screen from their lab, the authors report that knockdown of APOBEC3A decreases the number of nucleoli in a cell to a similar extend as knockdown of a known ribosome assembly factor while simultaneously cell viability is decreased. At the same time, progression through the cell cycle is perturbed which is likely a result of the nucleolar stress response pathway as p53 and CDKN1A levels are increased. Defects in ribosome biogenesis are further supported by the observation that global protein synthesis is decreased. Upon investigating how APOBEC3A knockdown influences ribosome biogenesis, the authors uncovered that 28S rRNA formation is perturbed and that a 32S pre-rRNA intermediate accumulates whereas the subsequent 12S precursor of 5.8S rRNA is decreased further corroborating the role of APOBEC3A in ribosome biogenesis. By isolating and sequencing nucleolar RNA in wildtype and APOBEC3A knockdown cells, the authors report potential C-to-U editing sites in pre-rRNA and in pre-mRNA suggesting that the RNA editing function of APOBEC3A plays a role in ribosome assembly.
Overall, the authors provide solid evidence that the knockdown of APOBEC3A affects ribosome biogenesis in human cells which is an unexpected and very interesting discovery. In particular, it is noteworthy that knockdown of APBOEC3A causes a change in nucleolar number and affects pre-rRNA processing. As a result of these ribosome biogenesis defects, it is fitting that the nucleolar stress response is triggered resulting in cell cycle perturbations, and it is equally fitting that global protein synthesis is decreased.

The critical question triggered by these observations is whether APOBEC3A is directly involved in ribosome assembly and if so, what its mechanistic role is. While the authors offer some carefully phrased suggestions that APOBEC3A's RNA editing function could play a direct role, these data require further validation and do not provide a mechanistic link at the moment. Thus, the current version of the manuscript reports an interesting involvement of APOBEC3A in ribosome biogenesis, but it does not address whether and how APOBEC3A directly impacts ribosome assembly.

The following major questions and suggestions are aimed at assessing whether APOBEC3A is directly involved in ribosome biongenesis and whether the editing of pre-rRNA can be validated. It is essential for the authors to address both aims in order to strengthen the manuscript for publication. This could be achieved by addressing my major questions and suggestions below or potentially through other approaches:

1. In order to directly link the action of APOBEC3A to ribosome biogenesis, it would be helpful if the authors could address at least some of the following questions:
o Is APOBEC3A located in the nucleolus? And if yes, is it re-distributed away from the nucleolus when ribosome biogenesis is perturbed?

See Reviewer 1, Comment 1 and 3 Response

Previous studies have suggested APOBEC3A to be pan-cellular [Reviewed in Smith et al. (2012), Lackey et al. (2013), Revathidevi et al. (2021)]. However, these results have not been without their caveats. The first difficulty is due to APOBEC3A possessing about 90% protein sequence identity to its family member, APOBEC3B, which is a gene duplicate of APOBEC3A (Viera and Soares 2013). Additionally, there are several other family members with high homology, including APOBEC3G. Therefore, it is difficult to produce a specific antibody and only probe for APOBEC3A in subcellular localization studies. For example, an entire study was published devoted to development of a APOBEC3B monoclonal antibody, yet this antibody still had cross-reactivity with APOBEC3A and 3G (Brown et al. 2019). Another difficulty is that APOBEC3A is very lowly expressed in most cell types, making it hard to reliably detect (Swanton et al. 2015, Jalili et al. 2020).

Because of these difficulties, we chose to use an overexpressed C-terminal 3X-FLAG tagged version of APOBEC3A for localization studies in HeLa cells. We performed immunofluorescence using an anti-FLAG antibody and co-stained with DAPI (nuclei) and anti-fibrillarin (nucleoli). Our results show that APOBEC3A is pan-cellular but enriched in the nucleus (colocalization with DAPI staining) and is also found in the nucleolus (colocalization with fibrillarin staining) (**NEW FIGURE 4A, B**). This result points towards APOBEC3A having the ability to directly function in ribosome biogenesis within the nucleolus.

o Does APOBEC3A interact with other ribosome assembly factors (e.g. in immunoprecipitations)?

Thank you for this suggestion, we have performed these experiments.

See to Reviewer 1, Comment 1 and 3 Response

o Does APOBEC3A interact directly with pre-rRNA? This could for example be assessed through crosslinking studies followed by APOBEC3A immunoprecipitation and RNA sequencing.

We did not directly address this question, nor has it been addressed in previous APOBEC3A research. We were happy to make progress by observing that a subset of APOBEC3A-FLAG localizes to the nucleolus and associates with nucleolar proteins (**NEW FIGURE 4**) and we view this as a potential experiment for future studies.

2. To directly link the editing function of APOBEC3A to ribosome biogenesis, it would be useful to replace APOBEC3A wt with an RNA-editing inactive APOBEC3A variant to separate catalytic effects from RNA-binding effects on ribosome biogenesis.

Thank you for the suggestion, we have answered this question by performing experiments overexpressing wild-type or a catalytically dead mutated version of APOBEC3A and comparing.

Refer to Reviewer 1, Comment 1 and 3 Response

3. Can the authors rule out that APOBEC3A has edited the rDNA rather than the pre-rRNA? Presumably, this cannot be addressed through rDNA sequencing due to the large number of rDNA genes, but is it possible to probe for edited rDNA by Southern blotting?

We cannot rule out that APOBEC3A has edited the rDNA, however, our results points towards this likely not being the case. First, our new results are after acute depletion of APOBEC3A for only 3 days, while DNA sequencing experiments searching for APOBEC3A mutational signatures are either in patients or completed over several numbers of passages in cell culture (Petljak et al. 2019, Petljak et al. 2022).

4. To validate the reported C-to-U editing events, the authors must identify a suitable positive control, e.g. a known site in RNA that is edited by APOBEC3A, and quantify its editing level. This would help to assess whether a 50% reduction in editing upon APOBEC3A knockdown is expected (or whether a stronger decrease should be observed).

We agree, a positive control would have been great to have in this experiment, but we are not certain what that would be for the rRNA. Previously detected APOBEC3A candidate edit sites on RNAs come from polyA enriched RNA sequencing experiments on mature mRNAs or more directed experiments on specific mRNAs, while our experiment on nuclear total RNA significantly has much fewer reads than mature mRNAs (**Now Figure 7B**). Therefore, most of the reads we detected align to the pre-rRNA (~54%) and about half of the edit sites detected (~46%) are present in intronic regions of pre-mRNAs. We argue this offers novelty and a new type of experiment rather than a repeat of what has been completed previously, overall enhancing its appeal/importance to a broader community.

We do feel it is important to note that we attempted to compare to previously published datasets of APOBEC3A RNA edit sites and did not detect any overlapping sites with ours (Alqassim et al. 2021, Jalili et al. 2020, Sharma et al. 2016).

Further validation would be possible with our new overexpression experimental setup; however based on our results with the catalytically dead version of APOBEC3A it appears its editing activity is not required for its role in making ribosomes. Although we detected several (pre)-mRNAs that encode nucleolar proteins with APOBEC3A predicted target sites (**Now Figure 7F, Data S6**), it is likely an auxiliary effect compared to a direct role within the nucleolus. This fact, combined with the difficulty validating a large number of edit sites, led us to believe this is beyond the scope of our current work presented here.

5. The reported editing frequencies in pre-LSU rRNAs are very low ("modest"). How can the signal be distinguished from background? What is the expected error frequency to detect an apparent C-to-U change? It is conceivable that both misreading during sequencing as well as errors of the RNA-Polymerase contribute to the observed C-to-U changes. Can these contributions be quantified and distinguished?

The pre-rRNA consensus sequence aligned to over 50% of the total nuclear RNA sequencing reads. This produced a high read depth across the entire pre-rRNA (47S pre-rRNA average coverage depth = 410,163, 5S rRNA average coverage depth = 136,177), even including spacer sequences which are cleaved out during maturation. Therefore, we achieved enough sensitivity to reliably detect very low frequency edits down to less than 0.01%.

We were careful to include a non-targeting siRNA (siNT) as a negative control to use as a comparison to protect us from observing potential artifacts like polymerase errors or sequencing misreads. We only considered an edit to be a predicted APOBEC3A site when it was reduced in both APOBEC3A siRNA depletion conditions in both replicates, further reducing the likelihood that our observations are from experimental noise or other issues.

6. The authors seem to imply that editing of pre-rRNA by APOBEC3A may impair further processing of the 32S pre-rRNA. Can this hypothesis be directly tested? For example, is it possible to transfect "edited" pre-rRNA containing C-to-U mutations or to induce the expression of such an pre-rRNA variant and to following its processing?

Experiments like this have been done in the laboratories of Markus Bohnsack and Sebastian Klinge. However, while they have yielded important and interesting results, they do not recapitulate all the steps that occur during the making of ribosomes, so we have not included them here.

Instead, our results showing that the catalytically dead version of APOBEC3A is able to increase translation and cell growth similarly to the wild-type after overexpression (**NEW FIGURE 5**) indicates that it is likely APOBEC3A’s binding and not editing ability that are required for its role in making ribosomes.

7. The authors report the interesting finding that APOBEC3A is overexpressed in certain cancers and that this overexpression (modestly) correlates with survival. Based on this finding, it would be very interesting to understand the effects of APOBEC3A overexpression on ribosome biogenesis and cell cycle progression.

Thank you for your curiosity and asking us to attempt these experiments. From them we have gained tremendous insight into APOBEC3A’s function in ribosome biogenesis and cell growth in a cancer cell line. First, we optimized transfection conditions of a CMV promoter-controlled C-terminally 3X-FLAG tagged APOBEC3A containing plasmid in MCF10A (non-cancerous) and HeLa cells (cancerous). A much lower amount of plasmid was needed to reach readily detectable levels of APOBEC3A-FLAG protein in HeLa cells compared to MCF10A cells (**NEW FIGURE S7**). Consequently, we utilized HeLa cells for overexpression experiments with the added bonus of its relevance as a cancer cell line. We also included an APOBEC3A C106S mutant as a comparison which has been shown before to work as a catalytically dead mutated version (Taura et al. 2022, Taura et al. 2019, Mitra et al. 2014, Chen et al. 2006) and an empty vector (EV-FLAG) as a negative control.

We overexpressed APOBEC3A wild-type and C106S mutant for 48 hours in HeLa cells to different extents by increasing amounts of plasmid added. Then we measured cell growth over that time period by a CellTiter-Glo® 2.0 assay for cell growth compared to a negative EV-FLAG control. With clarity, both wild-type and C106S mutant versions of APOBEC3A increased cell growth compared to EV-FLAG. Furthermore, this increase was dependent on the amount of plasmid transfected, with higher amounts of plasmid used resulting in a greater increase in cell growth (**NEW FIGURE 5B**). More precisely, we next tested APOBEC3A’s overexpression’s effect on translation levels after 48 hours. We used the same concentration of overexpression plasmid that also showed significant increases in cell growth for this assay. Consistent with both APOBEC3A wild-type and C106S mutant overexpression increasing cell growth, we observed an increase in global protein synthesis by puromycin incorporation after overexpression (**NEW FIGURE 5C**). Like the growth assay, this increase in translation was observed to increase with increasing amount of transfected plasmid (**NEW FIGURE 5D**). Our results show the APOBEC3A wildtype or C106S mutant overexpression produces the inverse effect as APOBEC3A depletion did where cell viability and protein synthesis were decreased. These results are initial findings, and from our understanding the first of their kind, showing that APOBEC3A overexpression can drive increases in the final output of ribosome biogenesis of translation and ultimately cell growth independently of its catalytic activity.

8. If APOBEC3A indeed directly affects ribosome biogenesis by editing pre-rRNA, then it will have to compete with snoRNAs and pre-rRNA folding and assembly during the very early stages of ribosome biogenesis. It's likely not easy to test this competition, but perhaps editing frequencies increase when the snoRNA machinery is reduced (e.g. by decreased expression of Fibrillarin or Dyskerin).

It is true that many ribosomal proteins, assembly factors, and snoRNPs bind the pre-rRNA during its biogenesis in the nucleolus. It is also very clear that they do not all bind at the same time, as ribosome biogenesis is a dynamic process. Furthermore, depletion of one essential, abundant factor like fibrillarin or dyskerin can affect many downstream events. We prefer to reserve this experiment for the future.

Minor suggestions:
- Line 132: define the "one-nucleolus percent effect" before introducing it.

Thank you for pointing out this lack of clarity for these initial results. We have included a sentence now explaining how the “one-nucleolus percent effect” is quantified:

“We quantify this as a “one-nucleolus percent effect” where the average number of nucleoli in a given treatment are compared to the negative control non-targeting siRNA (siNT) which is set to 0% and the positive control (siNOL11) which is set to 100%.”

- Line 308: "47/45S pre-rRNA levels" is misleading as it can be interpreted as a ratio 45S and 47S when it is really their combined detection.

Thank you for pointing out this misleading reference to our qRT-PCR results. We have switched this text to “47S+45S pre-RNA levels”.

- Fig. 4F: why is the RNA (18S, 28S) migrating slower in the siRNA lanes?

We apologize for this confusion. Our gels were mysteriously migrating at slightly different rates across the lanes for an extended period of time. This is not a consistent pattern that can be attributed to anything happen biologically within the cells we harvest total RNA from. We have shown here the best replicate that we have.

- Fig. 6E: what is meant with "coding (intron)" versus "noncoding (intron)"? Also, comparing 6B and 6D, it seems that introns are less enriched in the edited locations compared to the total nuclear RNA being sequenced.

“Coding (intron)” is within an intron of a pre-mRNA transcript, while “noncoding (intron)” is within an intron on a non-coding RNA. More long-noncoding RNAs and identification of exons and introns within these transcripts have come to light in recent years (Reviewed in Khan et al. 2021).

Our reads aligned to about 62% fully intronic regions, while the predicted target sites were found in introns at ~46% of the time. We would expect the read alignment to be consistent with the locations of the APOBEC3A predicted target sites. However, it is unclear if this ~16% reduction is meaningful, especially considering our stringent cutoff only resulting in a small number of 264 high-confidence target sites. Additionally, there were slight differences in analyses undertaken, where the read alignment quantification was completed by quantifying the align reads to the Ensembl Transcripts release 100 and the APOBEC3A predicted target sites were analyzed using Ensembl Variant Effect Predictor (McLaren et al. 2016).

We have made sure to include this info now in our methods sections to help with clarity.

- Fig 6E: is the change in the weblogo comparing the potential and the candidate edit sites significant? The increase in T before C seems very small given the small data set.

We have now tested for significance for enrichment of the UC sequence motif in our detected APOBEC3A predicted edit/target sites. We used a Fisher’s exact test and did not observe any significant difference between the number of UC sequence motif’s we observed compared to what would be expected at random. All 264 sites (expected = 66, observed = 60, p =0.6) and top 16 sites (expected = 4, observed = 6, p = 0.7). We have refrained from including this analysis in our manuscript due to the difficulties analyzing this small of a data set. However, we still include the sequence logos to reflect that we did not observe the expected UC sequence motif in our edit sites. We discuss possible reasons for this in our manuscript including that we analyzed nuclear RNAs only, which greatly enriched for intronic regions that have never been analyzed for APOBEC3A targets previously.
