# Supplementary material for: The cytidine deaminase APOBEC3A regulates nucleolar function to promote cell growth and ribosome biogenesis
Source: PLoS Biol. 2024 Jul 8;22(7):e3002718. doi: 10.1371/journal.pbio.3002718 (PMC11257408; doi:10.1371/journal.pbio.3002718)
Supplement: Attachment — Submitted filename: 4.28.24_3AReviewerResponse_FORSUBMISSION.docx [file pbio.3002718.s023.docx]

Response to reviewers

We thank the reviewers for their time and consideration in offering constructive comments to aid us in improving our manuscript. In addition to responding to each reviewer’s specific comments, we have listed the major changes to our manuscript here:

- **DELETED** Figure 4D.
- **DELETED** Old Figure S13.
- **MOVED** Table 1 to Supplemental (**NOW Data S6**).
- **MOVED** Figures 6 and 7 to Supplemental (**NOW Figures S8, S10, S13, S14, S16, and S17**).
- **NEW** Discussion section paragraph based on immunofluorescence and co-immunoprecipitation results.
- **REVISED** Discussion Section. Moved order of paragraphs for more logical presentation. Added sentences and some rewording to discuss our results more aptly. Deleted less important paragraphs on RNA sequencing and variant analysis technical details to focus on most pertinent points focusing more on APOBEC3A’s function in ribosome biogenesis.
- **ADDED** 5 new references to better contextualize the results.

Changes to the text of our manuscript are displayed by blue text in the tracked changes version of our updated manuscript.

Reviewer #1: During the revisions the authors have provided several additional experiments aimed at deciphering the role of APOBEC3A in ribosome assembly.  Using an overexpression system the authors demonstrate that overexpression of both WT and catalytic deficient APOBEC3A increases cell growth and protein synthesis, suggesting that the editing activity of APOBEC3A is not required for ribosome assembly.  Immunofluorescence staining and co-IP analysis further establish that APOBEC3A is present in the nucleolus and associates with several nucleolar proteins. While the authors have some data to support a modest role of APOBEC3A in editing pre-rRNA, the additional experiments strongly support a non-editing role for APOBEC3A in ribosome assembly.  Overall this manuscript is well written and provides compelling evidence that APOBEC3A plays an important role in promoting ribosome assembly.

Minor Comment:
In Fig. 5C it would appear that the C105S mutation expresses at a much higher rate than WT APOBEC3A, suggesting that this mutation may increase protein stability. Have similar observations been made with catalytic mutants of other APOBEC proteins?

To our knowledge, this observation has not been made with other catalytically dead versions of APOBEC3A or any other APOBEC family members. Based on our results, it is unclear why the catalytically inactive mutated version of APOBEC3A has a higher protein abundance compared to wild-type (Fig 5E). We observe a trending increase in puromycin incorporation with overexpression of the catalytically dead version compared to wild type but it is unclear if this is due to the increased levels or to some other mechanism. We think it is an interesting observation to include but refrain from speculating further at this point. More extensive investigation would be required to decipher the precise mechanism.

Reviewer #2: In this revision the authors have added significant new material to address concerns raised from the previous submission. They have also revised the text to address prior issues. While the authors have clearly established that APOBEC3A is required for efficient ribosome biogenesis, a major point that I and the other two reviewers had raised regarded the question of whether or not the catalytic activity of APOBEC3a was required for this function. This question begins to get at the mechanism by which APOBEC3A acts. The straightforward way to address this question suggested by all three reviewers was to complement the knockdown of APOBEC3A with a catalytic mutant. In this revision, the authors have chosen to overexpress WT and catalytic mutant APOBEC3A and they show that overexpression of either promotes cell growth and translation. This is a rather different matter which raises other issues while not addressing the question at hand. I am puzzled by why the authors did not do the obvious experiment suggested by all three reviewers. As such, this does not adequately address my previous concerns. Many other minor issues have now been addressed in this revision.

Major points
1. The authors clearly showed that knocking down APOBEC3A resulted in reduced ribosome assembly. They showed this by several means, altered number of nucleoli, altered rRNA processing and reduced levels of mature 28S rRNA, all of which led to reduced levels of translation. An important mechanistic question was whether or not these effects were due to the catalytic activity of APOBEC3A, implying that editing an RNA target causes these changes. This would be a really interesting finding. This question could easily be addressed to a first approximation by determining if siRNA-resistant WT and catalytic defective APOBEC3A could rescue the knockdown, assayed by counting number of nucleoli and measuring 28S/18S levels by Bioanalyzer, as they did previously. Oddly, the authors chose rather to overexpress APOBEC3A. Despite the rationale they give for using overexpression, it is not necessarily expected that if knocking down APOBEC3A reduces ribosome levels then overexpressing APOBEC3A should increase levels. Ribosome assembly and its cellular control is highly complex and it is extremely unlikely that overexpression of a single biogenesis factor, especially one not intimately involved regulating rDNA transcription, will upregulate the pathway. More likely, overexpression of APOBEC3A modulates cell growth through other pathways and conclusions based on overexpression of APOBEC3A do not necessarily address the mechanistic question of whether APOBEC3A impacts ribosome biogenesis through editing a target substrate.

Thank you for the observation that we could more directly test if APOBEC3A’s editing activity is required for its role in ribosome production. We agree that inclusion of siRNA resistant rescue experiments using a catalytically dead mutated version of APOBEC3A would strengthen our claim that APOBEC3A’s catalytic activity is not required for it to regulate ribosome biogenesis. Unfortunately, currently, we have been unable to successfully produce stable cell lines with inducible expression of siRNA resistant versions of APOBEC3A WT or catalytically dead APOBEC3A C106S. In addition to reasons laid out in our previous reviewer response, this was one reason for using an overexpression system to further investigate APOBEC3A’s function in ribosome production and the nucleolus. Since the submission of the revised manuscript, we have solely been working on setting up the rescue experiments. Given our several failed attempts and current deadline for revision, we hope that the manuscript will be acceptable without these experiments. We have extensively reworked our claims to better reflect the extent of our current results. Below we outline our attempted strategy for the rescue experiments, the strengths of our successful results within the manuscript, and the refined interpretations used to describe these results keeping this singular limitation in mind.

We attempted both transient expression and stable cell line generation of HeLa cells containing inducible siRNA resistant version of APOBEC3A WT or catalytically dead C106S mutated version to test for rescue of the phenotypes [puromycin (protein synthesis) and Bioanalyzer (28S/18S rRNA ratio)]. First, due to ease of experimental setup, we cloned APOBEC3A WT or C106S catalytically dead mutated versions that are resistant to siAPOBEC3A #1 (Table S2) or empty vector into the same expression plasmid used for the described transfection experiments in our manuscript (Figures 4, 5). We chose this siRNA to rescue against because it produced the most robust results in our deconvolution screening (Figure 1D). We then performed identical transfections to our overexpression experiments in HeLa cells; however, this time siRNAs (either a negative control non-targeting siRNA or siAPOBEC3A #1) were co-transfected 6 hours prior to plasmid transfection (per ThermoFisher RNAiMax and Lipofectamine 3000 manufacturer’s recommendations). Although we were able to detect APOBEC3A protein expression 48 hours post-transfection of the siRNA resistant versions (WT and C106S) even in the presence of siAPOBEC3A #1, we did not observe reproducible rescue of the puromycin incorporation defect that we observed originally in HeLa cells (Figure S4). This is likely due to the lack of transfection efficiency of this kind of setup. Supporting this, in our immunofluorescence experiments only about 40% of the cells were positive for FLAG staining under the same conditions transfecting APOBEC3A-3XFLAG WT. Therefore, we quickly moved towards solely working on producing stable cell lines that express inducible siRNA resistant versions of APOBEC3A WT and C106S.

To stably incorporate an inducible siRNA resistant version of APOBEC3A (WT or C106S), we chose to utilize the T‑REx™‑HeLa (Flp-In) system. We used this setup and cell line for multiple reasons. Firstly, because it allowed for inducible expression of APOBEC3A which is important because of APOBEC3A WT’s ability to cause genomic mutations, which would accumulate as cells were passaged. Furthermore, this would allow us to produce stable cell lines where, after selection, all of the cells would be expressing the same siRNA resistant APOBEC3A (WT or C106S). Finally, this system has been successfully used in our laboratory previously (Bryant et al. 2024). In that example, we used T‑REx™-293 cells but since HEK-293T cells do not express APOBEC3A (Jalili et al. 2020), we purchased the equivalently engineered HeLa cell line for $2500.

In our attempt to produce stable T‑REx™‑HeLa cells for our rescue experiments, we cloned siRNA resistant versions of APOBEC3A (WT or C106S) or empty vector (same as above) into the pcDNA5/TO plasmid. After DNA sequence verification, we cotransfected pcDNA5/TO plasmid (containing APOBEC3A WT, C106S, or empty vector) and pOG44 (a plasmid containing a recombinase for genomic integration of the construct at the Flp-In site) into the T‑REx™‑HeLa cells. Two days post transfection we split the cells to the right seeding density for selection (~20%) and the next day we added the Hygromycin B selection antibiotic. Unfortunately, in two attempts, all the cells died after a few days of selection, indicating the construct was likely not incorporated into the genome. Due to this, we have not been able to carry out the rescue experiments since we have been unable to produce the necessary stable cell lines. Thus, we cannot more definitively test if APOBEC3A’s catalytic activity is required for its role in making ribosomes.

In light of the failed rescue experiments and to better describe our present results, we have reframed our overexpression results text to only state that APOBEC3A WT and catalytically dead mutated APOBEC3A C106S overexpression increase cell growth and protein synthesis. We no longer claim that this is a direct increase of ribosome production. As the reviews have pointed out, our experiments did not directly test this. While it is possible that these results are from indirect effects outside the ribosome biogenesis pathway, when considering our colocalization and coimmunoprecipitation results using a tagged version of APOBEC3A, Occam’s razor suggests that effects on ribosome biogenesis would be the simplest explanation. We showed that a subset of APOBEC3A resides in the nucleolus and interacts with key ribosome biogenesis factors RPA194 and FBL, with these interactions providing a potential mechanism by which APOBEC3A promotes ribosome production in an overexpression context. It is unclear to us how APOBEC3A overexpression would drive protein synthesis outside the ribosome biogenesis pathway---- this would be the most direct and simplest explanation considering all of our results.

In addition to the overexpression experiments, some of our other current results also support a non-catalytic role for APOBEC3A in ribosome production. Our observed “APOBEC3A predicted target sites” on the pre-rRNA demonstrated very low frequency edits, the opposite of what we would expect if it were using its catalytic activity. Perhaps APOBEC3A is just binding at these sites, as opposed to performing high efficiency C-to-U editing, which is what is crucial for pre-LSU processing. Furthermore, these predicted target sites were mostly on the pre-LSU rRNA, which correlates with APOBEC3A’s requirement for pre-LSU processing and maturation. Even with this in mind, we have still restrained our conclusions in such a way so as to suggest that they point towards APOBEC3A’s catalytic activity not being required for its role in making ribosomes in our Discussion.

As we pointed out in our previous version of the manuscript and in this revised version, APOBEC3A’s catalytic activity has been shown to not be required for other functions, including repression of HIV-1 reactivation and regulation of interferon-stimulated response elements (Taura et al. 2019, Taura et al. 2022). These other results give credence to APOBEC3A’s binding ability alone being integral to cellular processes. Our results suggest that ribosome biogenesis should be added to this list as well.

Furthermore, we have extensively reworked our Discussion section to better focus on our results implicating APOBEC3A’s role in ribosome production as opposed to focusing on its editing function or lack thereof in this context. Please refer to Reviewer 3 Major Suggestion 1 for more information on our updated discussion section.

We hope that the changes best reflect the limitations of our results and put them in context of what is currently known about APOBEC3A. Given APOBEC3A rise as a target for cancer therapeutics in very recent years, we believe it is imperative to disseminate the knowledge we gathered on APOBEC3A to the broader scientific community. We understand the drawback of not being able to include the rescue experiment but believe (and hope you will agree) that the strengths of our work make up for not being able to include it. We are excited that our publication will help to guide future APOBEC3A research, not only in the context of ribosome biogenesis, but in the broader field of cancer biology.

2. Regarding, rRNA editing, as noted in the earlier draft, the edit frequency for sites in 28S or ITS2 that were altered upon APOBEC3A depletion are all 1% or less with percent difference upon depletion 10% or less. It is difficult to imagine how these very modest changes can account for the reported impact on ribosome assembly. Considering the new conclusion that APOBEC3A's editing function is not important for its role in ribosome biogenesis, the entire section on rRNA editing now appears to be rendered moot. Perhaps this section should now be supplemental.

We agree that the low editing frequency and modest reduction after depletion is in accord with APOBEC3A’s catalytic activity not being required for its role in making ribosomes. As described in response to comment #1, although not tested directly in our experiments, these and all our results suggest APOBEC3A binding to the pre-rRNA and/or ribosome biogenesis factors is required for its function if it is directly processing and maturing the large ribosomal subunit.

We more explicitly and correctly emphasize the relevance of these critical experiments in the associated Results and Discussion. We now more directly suggest in the Discussion that these very low editing frequency sites, which correlate in location to our observed processing defect after APOBEC3A siRNA depletion, could be attributed to functional APOBEC3A binding at these locations but not functional editing. This is further substantiated by 3 of the 5 predicted target locations of low frequency APOBEC3A editing contained a UC sequence motif and were reproducible over two replicates and two different APOBEC3A siRNA depletion conditions.

Furthermore, as per the suggestion above, we have moved these results (**OLD Figures 6 and 7**) to the supplemental (**NOW Figures S8, S10, S13, S14, S16, and S17**).

Minor points
3. In Fig 1A, siNOL11 shows a percent effect >100 but is described as being set to 100%. This needs explanation.

Figure 1A is data from our laboratory’s original nucleolar number screen (Farley-Barnes et al. 2018). This screen utilized siUTP4 as the positive control which was set at 100% effect. siNOL11 was used for our subsequent validation rescreening with an updated siRNA technology (Fig 1C, D) and was set at 100% effect for those experiments. We have pointed this out more explicitly in the text for increased clarity regarding this. We now state “We quantify this as a “one-nucleolus percent effect” where the average number of nucleoli in a given treatment is compared to the negative control non-targeting siRNA (siNT) which is set to 0% and the positive control [siUTP4 in previously published screen (5) or siNOL11 in updated rescreen) which is set to 100%.”

4. Table 1 should be moved to the supplemental material as there is no indication that any of these targets has a role in modulating ribosome biogenesis through APOBEC3A function.

Thank you for pointing this out. We agree that to better emphasize our more impactful results, we have moved this table to the supplemental (**NOW S6 Data**).

5. Regarding editing of the DDX17 mRNA, as the authors note, a decrease of 4% is modest at best. Considering that follow up work could not show significance of this edit, the DDX17 work does not significantly contribute to the manuscript and should be deleted.

We feel is it important to include this negative result. It provides valuable information and context to the potential meaning of the predicted edits we have identified. Additionally, it likely rules out an indirect mechanism by which APOBEC3A could function in pre-60S processing. The more context and relevant results that we can provide, the further we can push the field’s current understanding of APOBEC3A’s function and help guide future research. In our opinion, hiding the result by not including it in our manuscript we feel would be disingenuous to the broader scientific community.

Reviewer #3: The authors have substantially revised their manuscript which is now entitled "The cytidine deaminase APOBEC3A promotes human ribosome biogenesis". The manuscript now includes several additional experiments in response to reviewers' comment. Importantly, the authors showed that overexpression of wt APOBEC3A creates the inverse phenotype to depleting it, namely an increase in growth and increased protein synthesis. Importantly, the same phenotype is observed when overexpressing catalytically inactive APOBEC3A suggesting that C-to-U editing is not required for this phenotype and likely not for the effects of APOBEC3CA on ribosome biogenesis. Moreover, the authors strengthened the evidence linking APOBEC3A to ribosome synthesis by showing that a subset of the protein is located in the nucleolus and that it interacts with certain ribosome biogenesis factors. Taken together, these data now convincingly demonstrate that APOBEC3A plays a role in ribosome biogenesis
that is independent of its catalytic activity and thus its mutagenic activity. The authors have accordingly revised the text and in particular the discussion.

Overall, the manuscript's quality is significantly improved, and I have only one major and a few minor suggestions.

Major suggestion:
1. The weakest point of the manuscript is the low level of editing detected by the authors. I appreciate that they have now removed the claim that editing is directly linked to ribosome biogenesis based on the new data with a catalytically inactive APOBEC3A variant. However, their remains an imbalance in the discussion where the authors FIRST discuss on 1.5 pages the editing activity which is misleading to the reader. Only in the remaining one page do the authors briefly discuss APOBEC3A's non-catalytic role in ribosome biogenesis which is the main, and most exciting finding of their study. I strongly recommend reversing the order in the discussion and expand on the possible mechanisms how APOBEC3A could play a role in ribosome biogenesis. For example, the authors do not comment on the significance of APOBEC3A's interactions with ribosome biogenesis factors and their functions.

Thank you for pointing this out, thus allowing us to revise and create a better discussion section of our manuscript. We have extensively changed and improved our Discussion. Below we list specific improvements to our Discussion Section:

1. Deleted two paragraphs about our nuclear RNA sequencing C to U variant analysis experiment including one focusing on our sequencing depth and the other on intron read enrichment. These paragraphs were not directly relevant to APOBEC3A’s function in ribosome production.
2. Deleted Figure S13 which was referenced within the deleted portion as it was not additive to discussion of APOBEC3A’s role in ribosome biogenesis.
3. Added new paragraph for discussion of our APOBEC3A-FLAG colocalization and co-immunoprecipitation results. We emphasize how APOBEC3A’s association with FBL and RPA194, provide insights into a potential mechanism for APOBEC3A’s direct involvement in ribosome biogenesis within the nucleolus.
4. Rephrased editing to targeting sites, emphasizing binding or association with the pre-rRNA as the likely APOBEC3A’s mechanism.
5. Reordered to put our colocalization and co-immunoprecipitation results discussion first, then overexpression experiments, and finally discussion of if APOBEC3A is acting directly or indirectly in ribosome production and if its through association with the rDNA or pre-rRNA.
6. We focused more on how our results suggest, particularity our overexpression experiments and observation of low editing frequencies, that APOBEC3A’s catalytic activity is not required for its role in ribosome biogenesis. Since our results did not directly test for this, we felt it best to include this point in the discussion section, as opposed to our results as before, to help guide future APOBEC3A research.
7. We added a sentence (described below in response to Reviewer 3 Minor Suggestion 7) to indicate how cytidine deaminase inhibitors would be unlikely to alter APOBEC3A’s role in ribosome biogenesis based on our results.

Minor suggestions:
1. L278-284: the authors need to re-phrase the sentences and correct the logic. Currently, they stated that "factors only required (for?) pre-rRNA transcription or maturation result in lower percent inhibition values ~ 50-80%". This makes sense and the authors report 50.8% nucleolar rRNA biogenesis percent inhibition. However, the next sentence contradicts these statements as the authors conclude "it is likely that APOBEC3A is not required for pre-rRNA transcription".

Thank you for realizing this error in our text. We have now correctly stated that “while factors only required pre-rRNA maturation result in lower percent inhibition values ~50-80%”. Thus, APOBEC3A falls within the range of factors required for pre-rRNA maturation but not pre-rRNA transcription. This results text is now consistent with the results that we are comparing to when we developed this nucleolar rRNA biogenesis assay (Bryant et al. 2022) (ref. 70).

2. L382: what is meant with "IF"? I assume the authors refer to immunoprecipitation.

L382 is the start of the paragraph where we are describing our immunofluorescence (IF) experiments to test for APOBEC3A colocalization with a nuclear marker (DAPI) and nucleolar maker (anti-fibrillarin). We define abbreviations for both IF and immunoprecipitation (IP) in the previous paragraph. We have double checked to ensure there are no mix ups between the two abbreviations that we used.

3. Fig. 5C and D show the same data with the only difference being the reference point for normalization. This is misleading, and Fig 5D should be removed.

This is correct. We did not mean to be misleading but wanted to emphasize that increasing APOBEC3A (WT and C106S mutated versions) increased protein synthesis, while increasing the amount of empty vector did not. To be more succinct and clearer, we have deleted Figure 5 panel D but kept the description of our results in the text.

4. Fig. 7E: in the results (L598f), the authors claim that "there was a slight enrichment of a preceding U present". This statement is not justified by the logo shown in Fig. 7E, and the fact that it is based on only 16 top sequences. The authors must remove this claim.

We appreciate this comment and it has allowed us to be more careful with our claims in regard to our sequencing data. As noted in our previous reviewer response, we performed a Fisher’s exact test and did not observe a significant enrichment in the number of UC sequence motifs at the candidate target sites compared to what would be expected at random. Therefore, we have restated this part of the sentence “a preceding U was the most prevalent nucleotide, however in this same dataset, this was not a significant enrichment compared to what would be expected at random (Fisher’s Exact test, p = 0.7)”. Now the claim is removed, but we still describe our limited data here to the best of our ability.

5. L693: The sentence should clarify that "it" means "APOBEC3A" and that "addition of siRNAs" means "addition of control siRNAs (siNT)".

Thank you highlighting these instances where we lacked clarity. We have now fixed the indicated text with these exact recommendations.

6. L702: the authors should clarify that targeted deep sequencing will only uncover DNA editing for mRNAs, but not for rRNA due to the large number of rDNA genes.

It is true there are a large copy number of rDNA genes in human cells with estimates ranging from 200-600 rDNA gene copies per haploid genome, and they display variation in sequence (Parks et al. 2018, Hall et al. 2021, Gibbons et al. 2015). This would cause difficulties in analyzing the rDNA for edit sites attributed to APOBEC3A as opposed to endogenous variation between rDNA loci especially when considering they have only been recently been fully sequenced and partially assembled in a single haploid cell line (Nurk et al. 2022). Due to the unconfirmed nature and difficulties sequencing the of the rDNA currently, we now refrain from including this as a potential future experiment in our discussion section. We have significantly reworked this paragraph to discuss whether APOBEC3A is acting on the rDNA or pre-rRNA in terms of our results and what is currently known in the field. Instead of proposing a future experiment, we now describe future directions much more generally in a conclusion sentence to this paragraph, “Our study serves as a first analysis of APOBEC3A in ribosome biogenesis; it is our hope that future studies will reveal more mechanistic detail regarding APOBEC3A’s function within the nucleolus.”

7. L708: when discussing cytidine deaminase inhibitors as potential future cancer therapeutics, the authors must also discuss that such inhibitors would likely not alter APOBEC3A's effect on ribosome biogenesis which is independent of the catalytic activity.

This is an important point that we missed. We have now added a sentence describing that our results potentially indicate that these inhibitors would only impact APOBEC3A’s editing (catalytic) activity and not its catalytically independent role in ribosome biogenesis that we have studied here. We now state: “Although if APOBEC3A’s editing activity is not required for its role in ribosome biogenesis, these inhibitors would be unlikely to alter that aspect of APOBEC3A’s cancer promoting ability.”
